# Supplementary material for: Synthesis of Mixed Dinucleotides by Mechanochemistry
Source: Molecules. 2022 May 18;27(10):3229. doi: 10.3390/molecules27103229 (PMC9147584; doi:10.3390/molecules27103229)
Supplement: Supplementary file 1 [file molecules-27-03229-s001.zip › molecules-1707212-supplementary.pdf]

Supplementary material

Synthesis of mixed dinucleotides by mechanochemistry

Faisal Hayat [1] , Mikhail V. Makarov [1], Luxene Belfleur [1] and Marie E. Migaud [1],*

1 Mitchell Cancer Institute, Department of Pharmacology, College of Medicine, University of South Alabama, 1660 Springhill Avenue, Mobile, AL 36604, USA; fhayat@southalabama.edu, lbelfleur@southalabama.edu, mimak78@gmail.com;

* Correspondence: mmigaud@southalabama.edu;

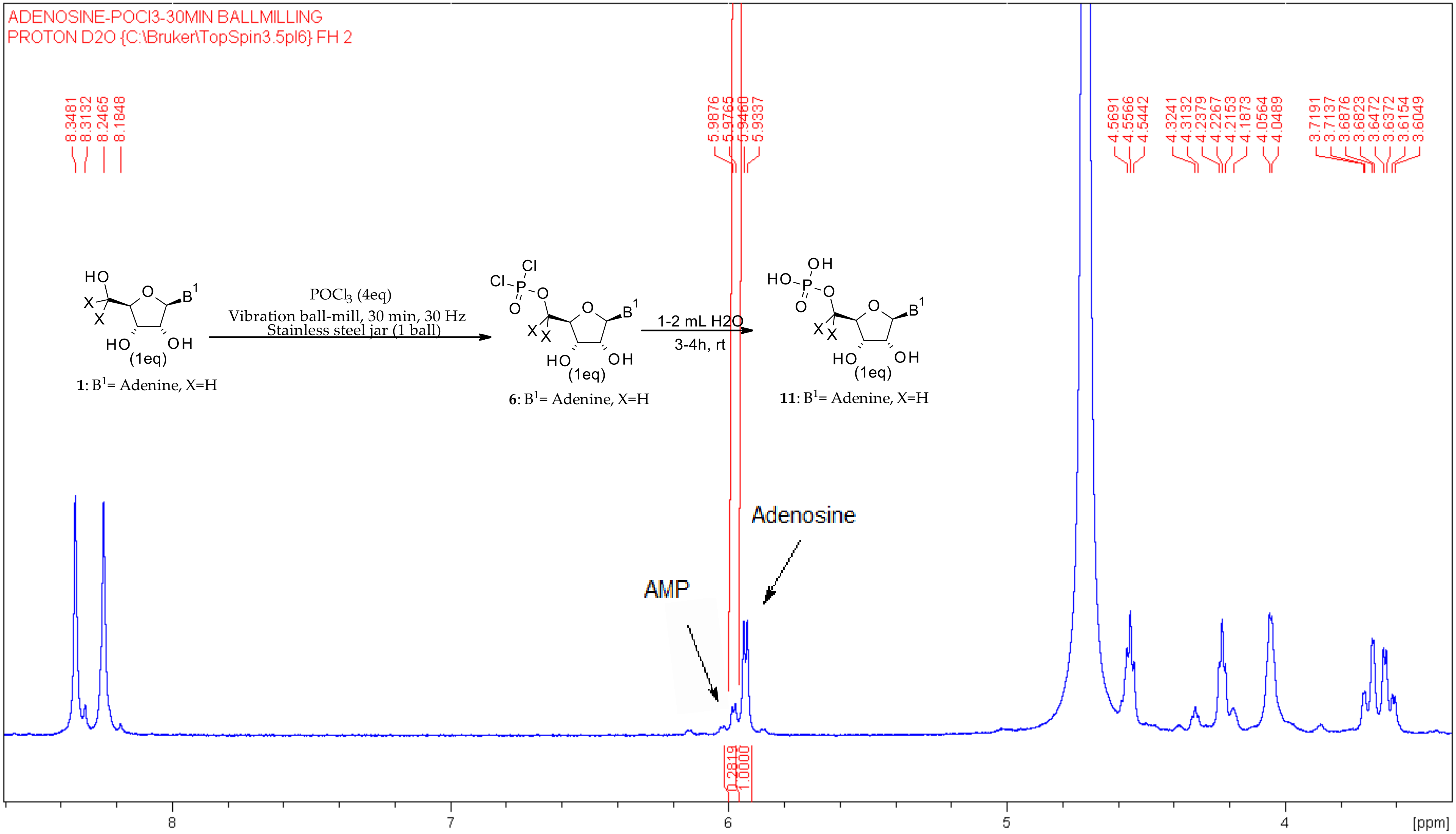

Compound 11: ^{1}H-NMR of crude product after 30 minutes of ball-milling (D_2O)

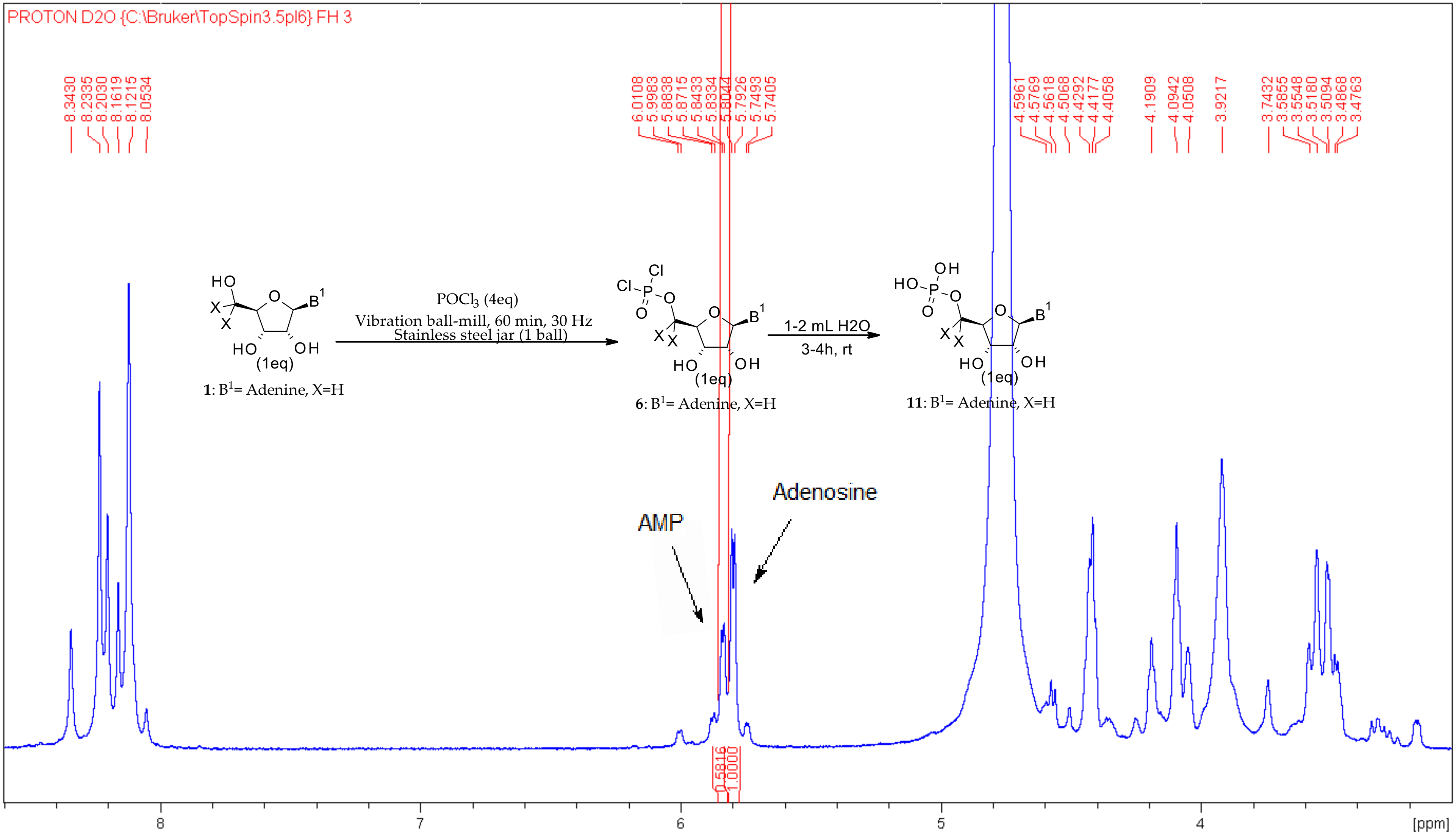

Compound 11: ^{1}H-NMR of crude product after 1 hour of ball-milling (D_2O)

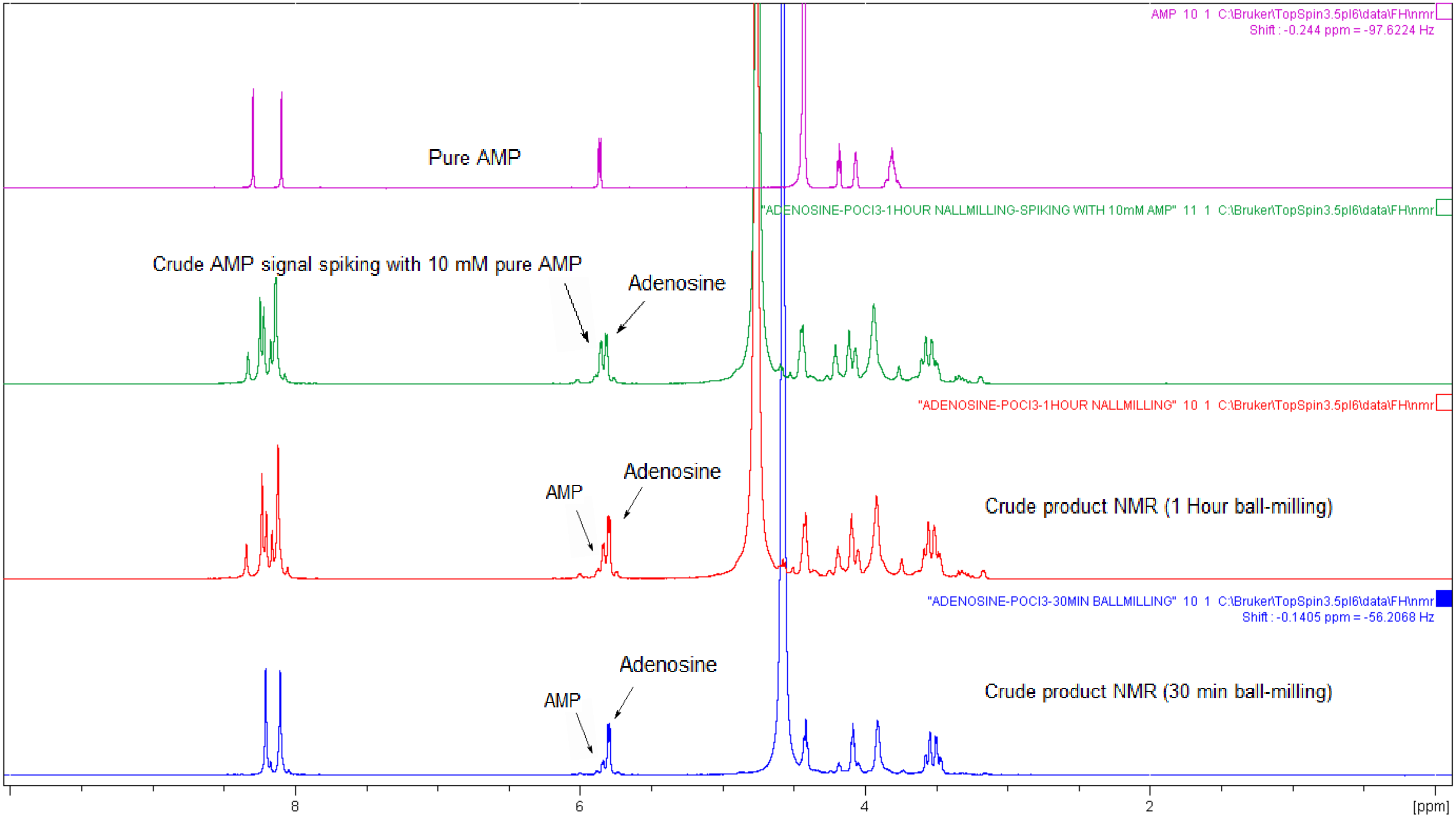

Compound 11: AMP Signals from a one-hour ball-milling crude product spiked with 10 mM pure AMP (D_2O)

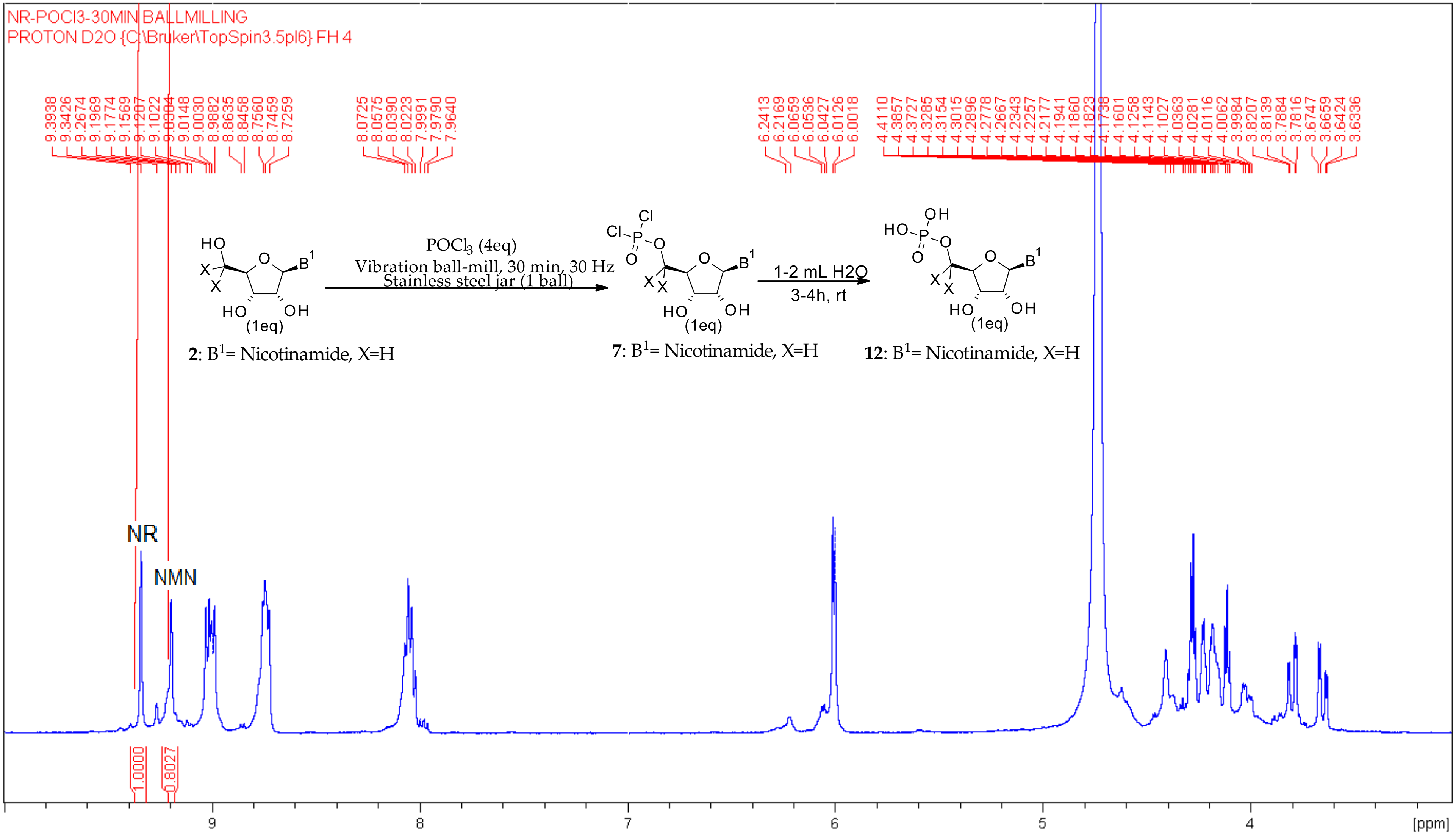

Compound 12: ^{1}H-NMR of crude product after 30 minutes of ball-milling (D_2O)

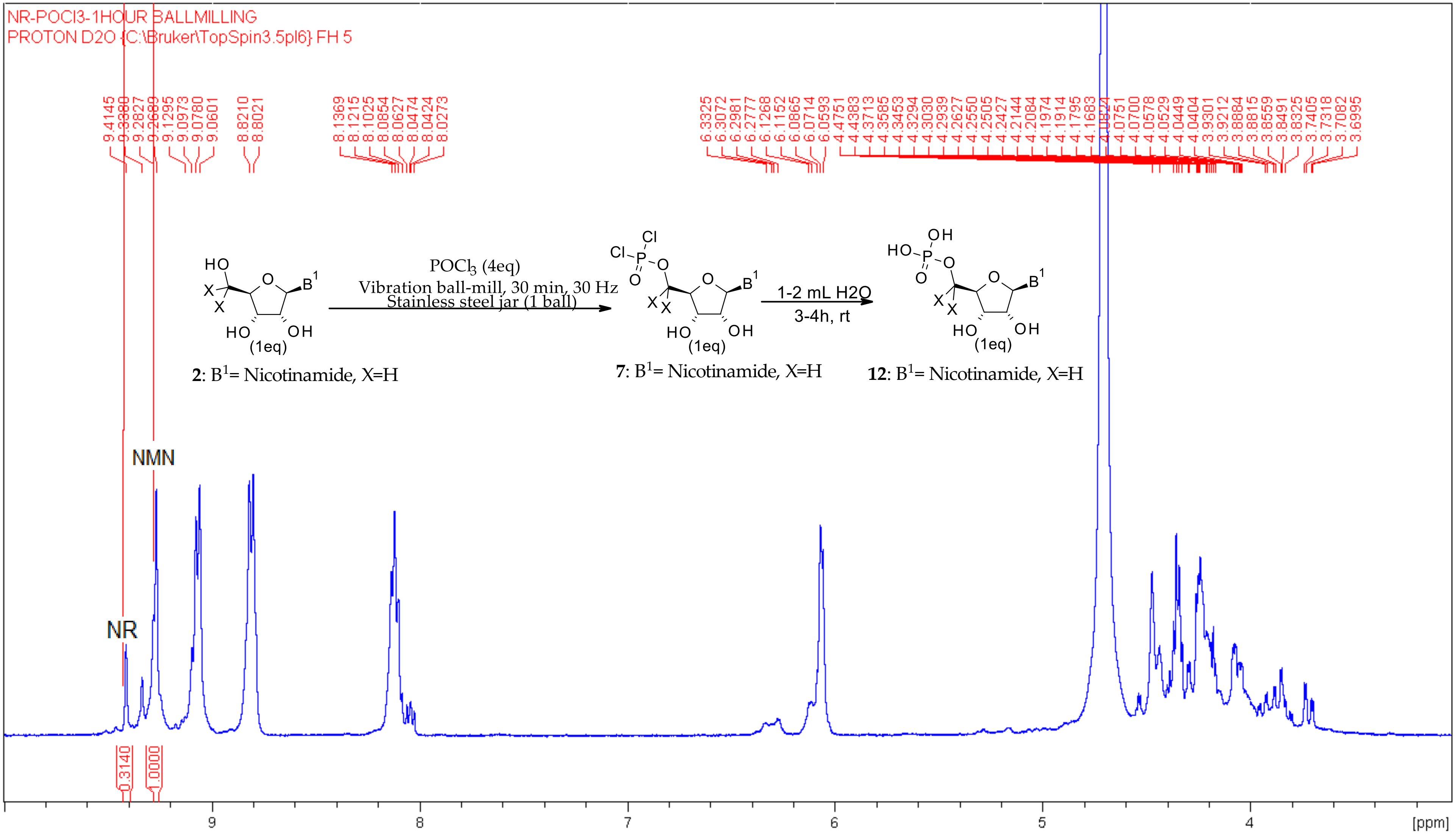

Compound 12: ^{1}H-NMR of crude product after 1 hour of ball-milling (D_2O)

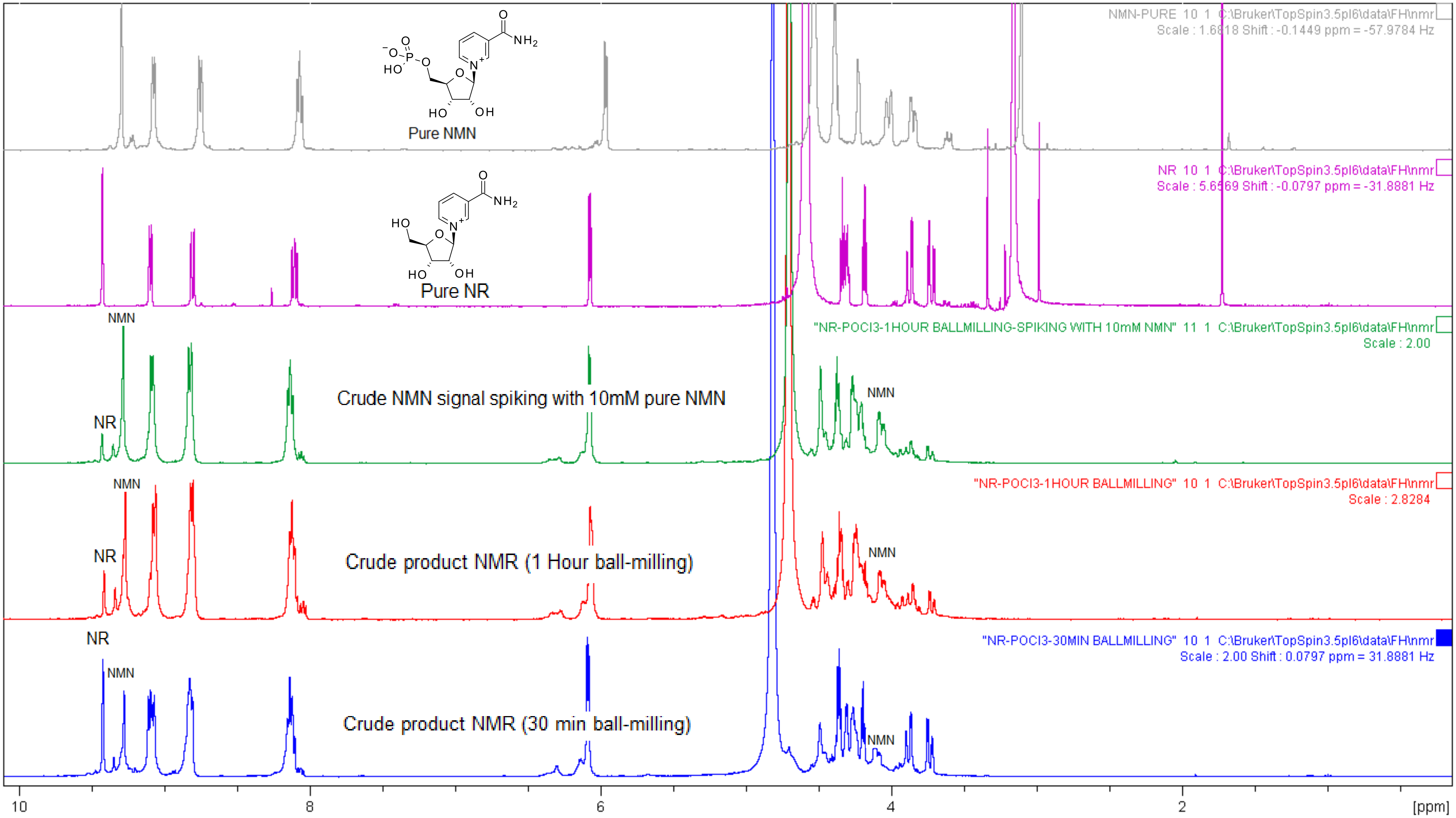

Compound 12: NMN Signals from a one-hour ball-milling crude product spiked with 10 mM pure NMN (D_2O)

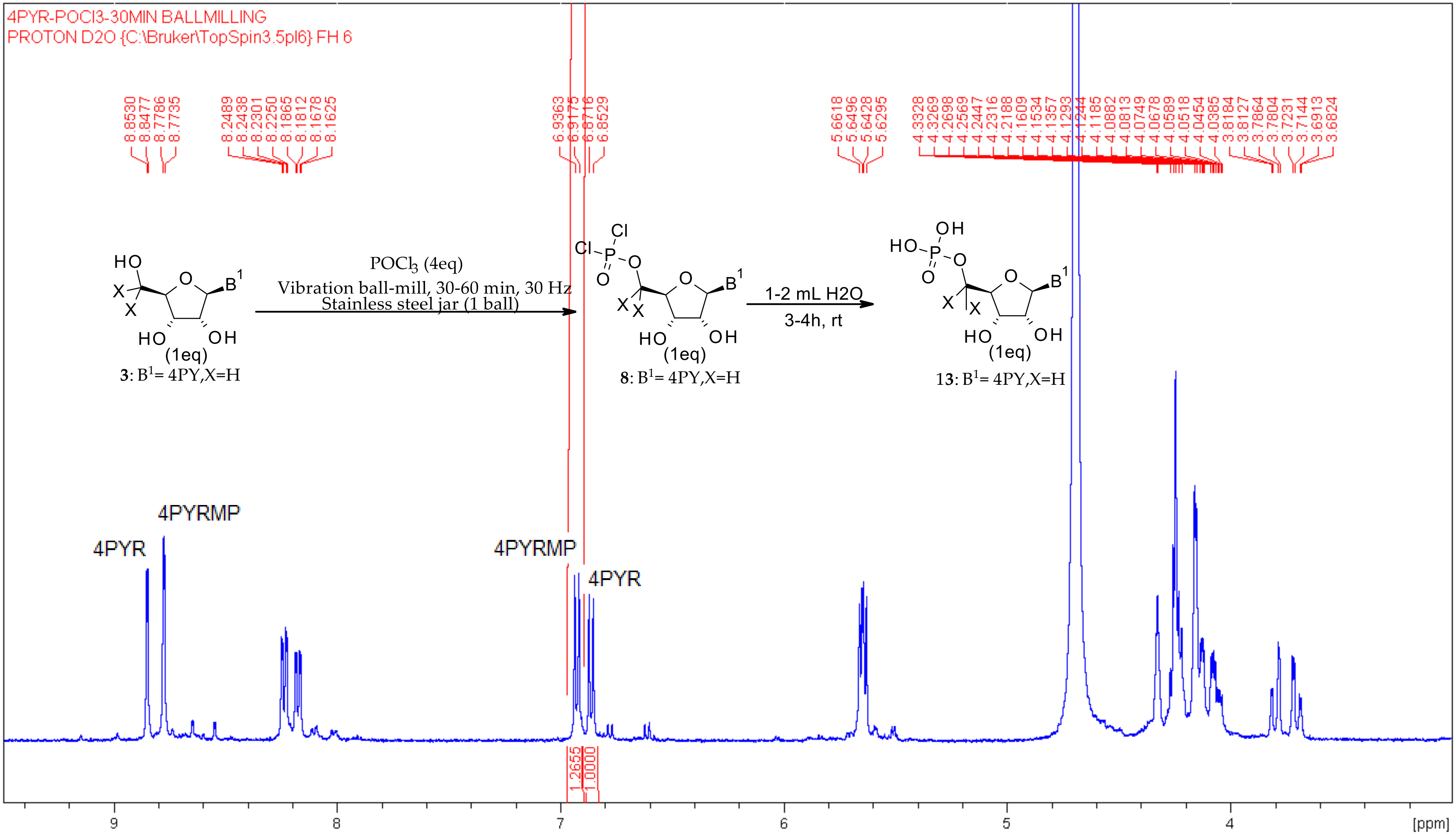

Compound 13: ^{1}H-NMR of crude product after 30 minutes of ball-milling (D_2O)

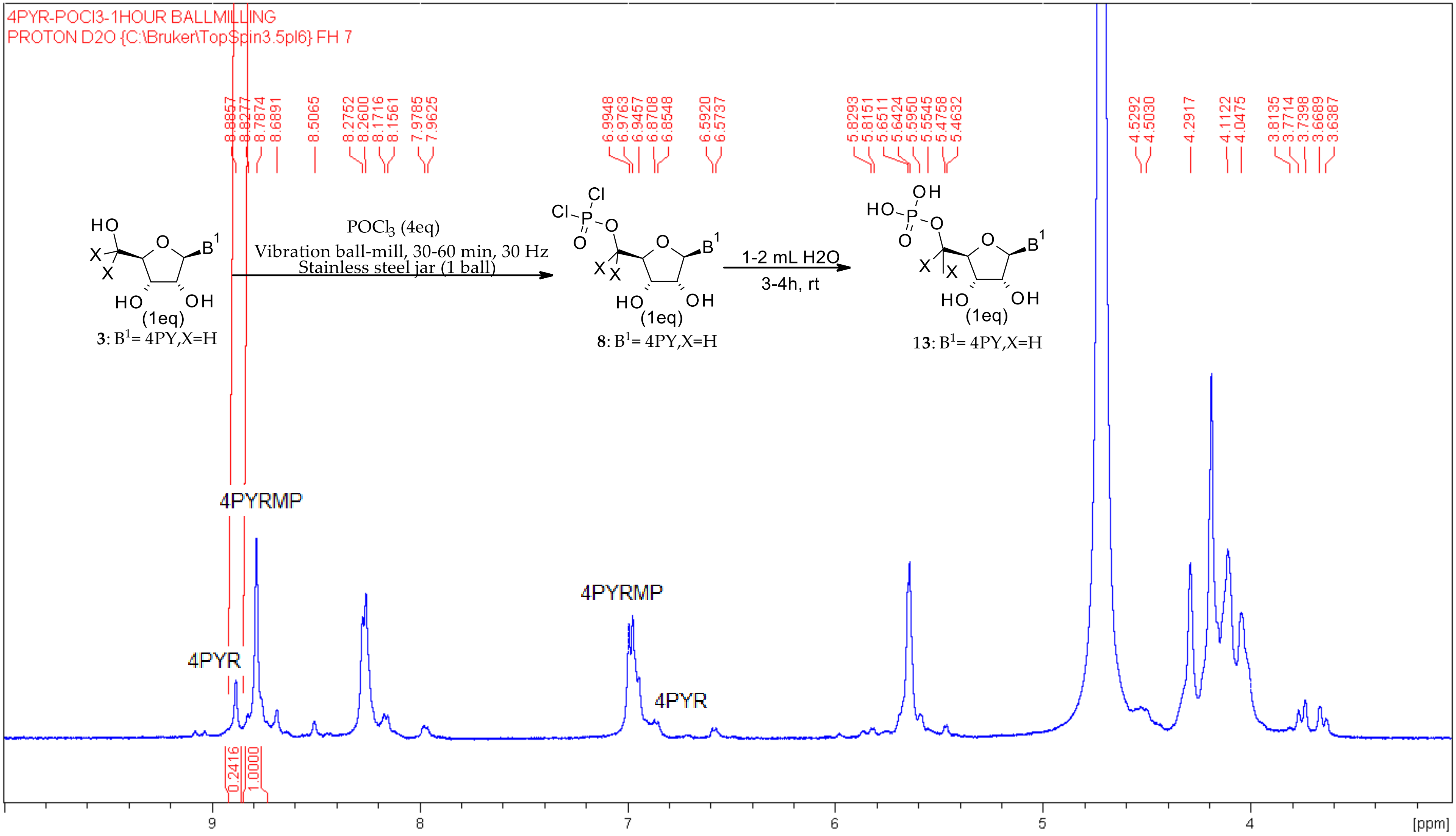

Compound 13: ^{1}H-NMR of crude product after 1 hour of ball-milling (D_2O)

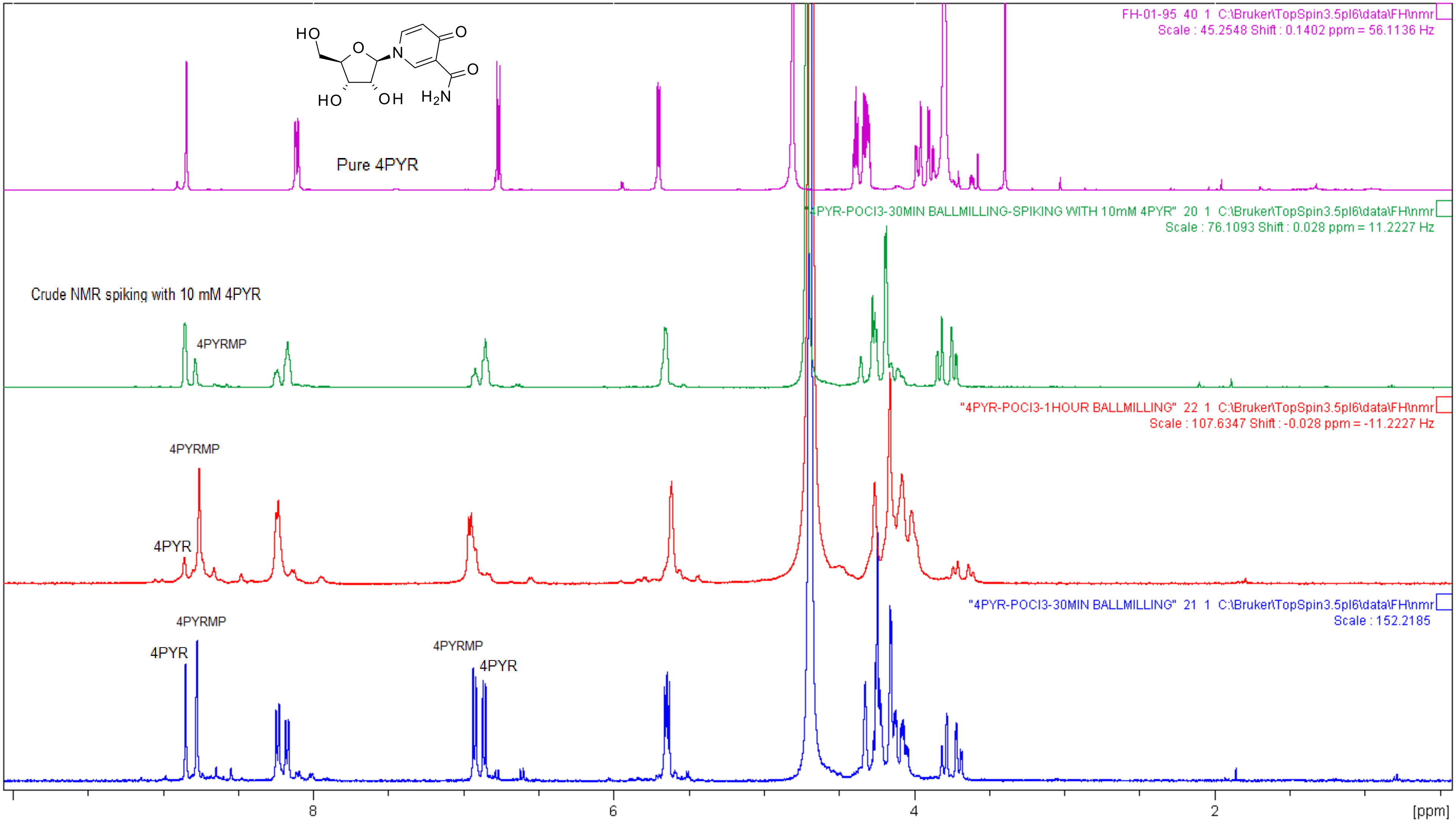

Compound 13: 4PYR Signals from a one-hour ball-milling crude product spiked with 10 mM pure 4PYR (D_2O)

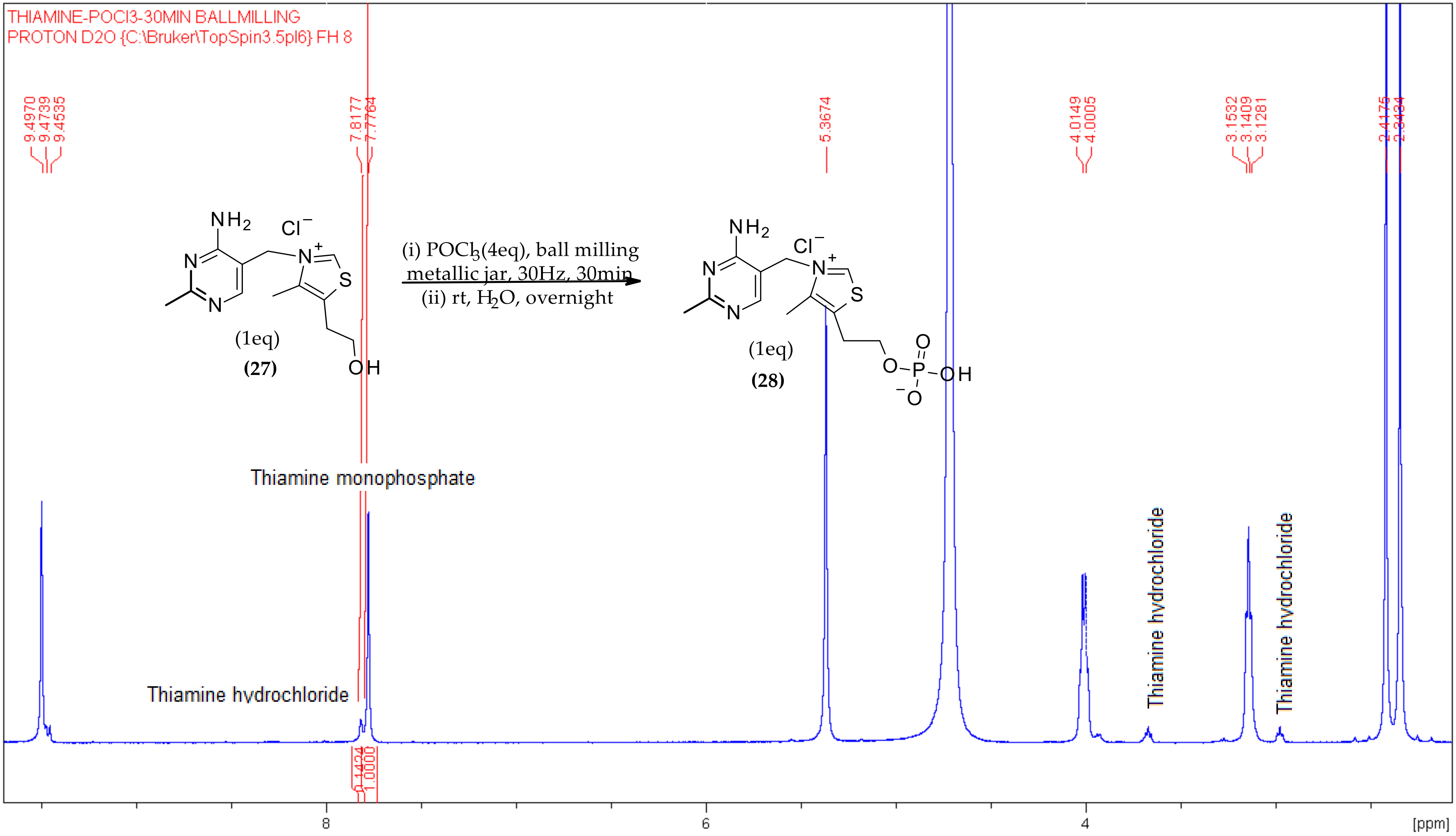

Compound 28: ^{1}H-NMR of crude product after 30 minutes of ball-milling (D_2O)

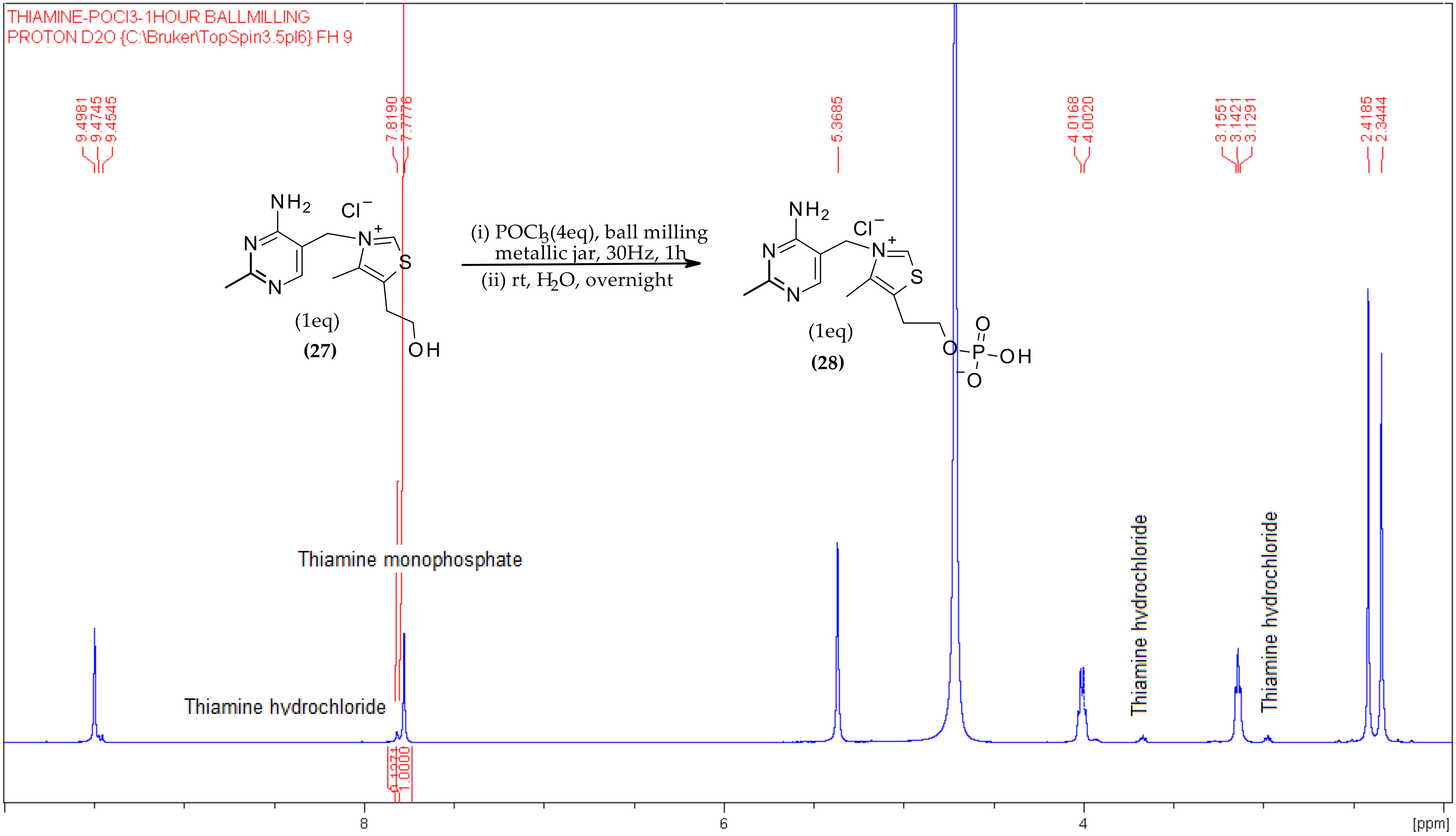

Compound 28: ^{1}H-NMR of crude product after 1 hour of ball-milling (D_2O)

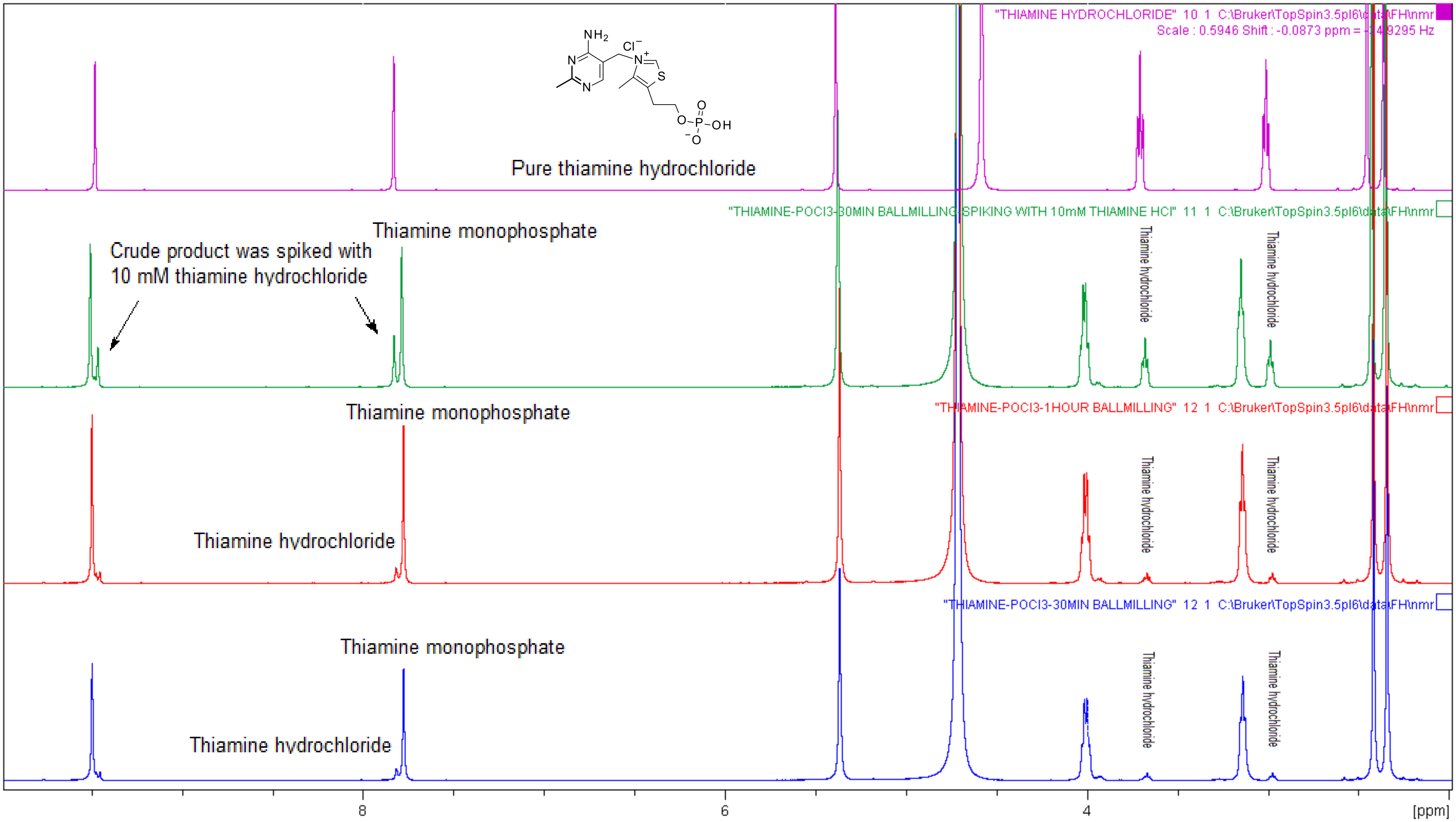

Compound 28: Thiamine Signals from a one-hour ball-milling crude product spiked with 10 mM pure thiamine hydrochloride (D_2O)

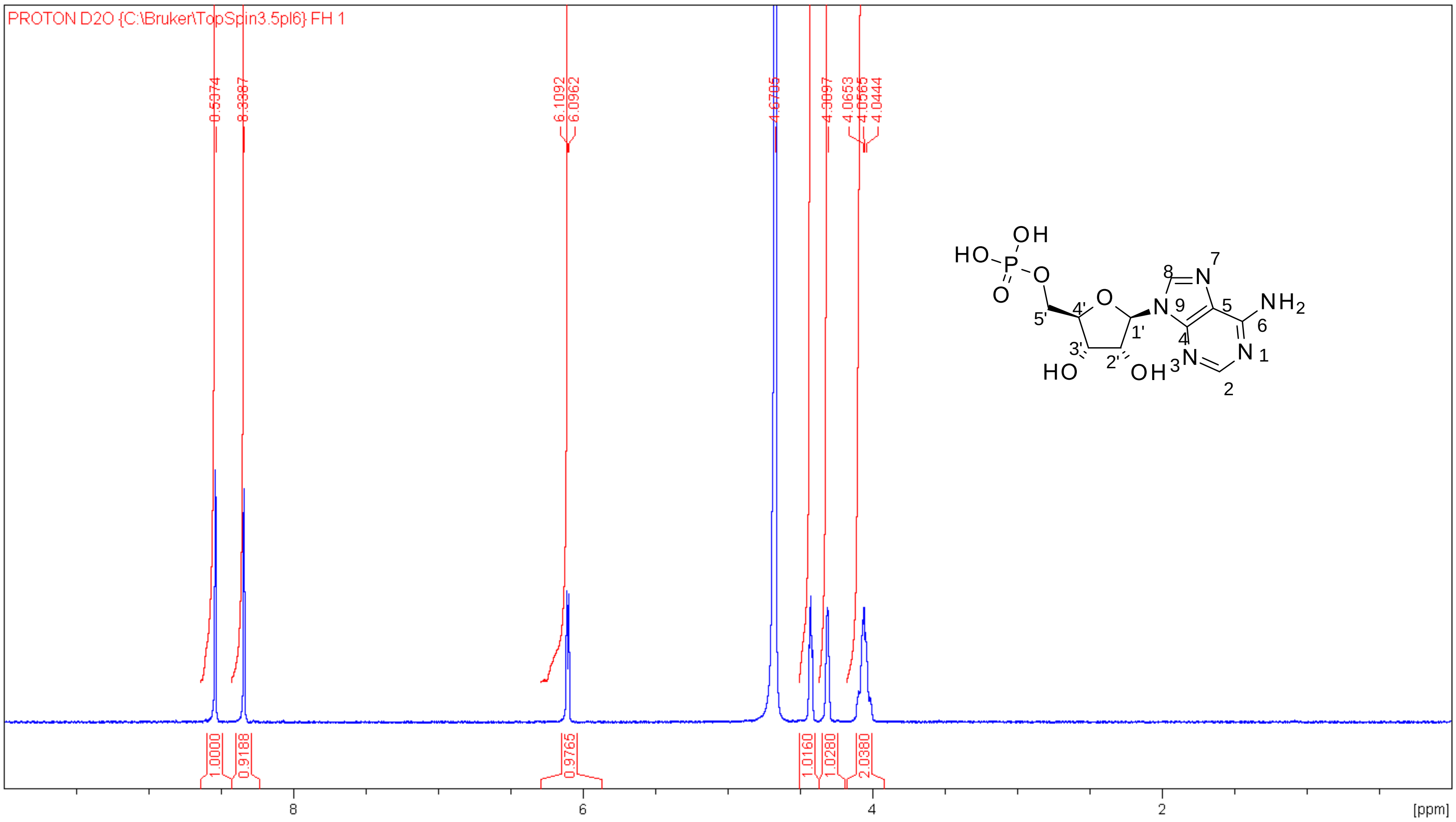

Compound 11. 400 MHz ^{1}H NMR spectrum in D_2O

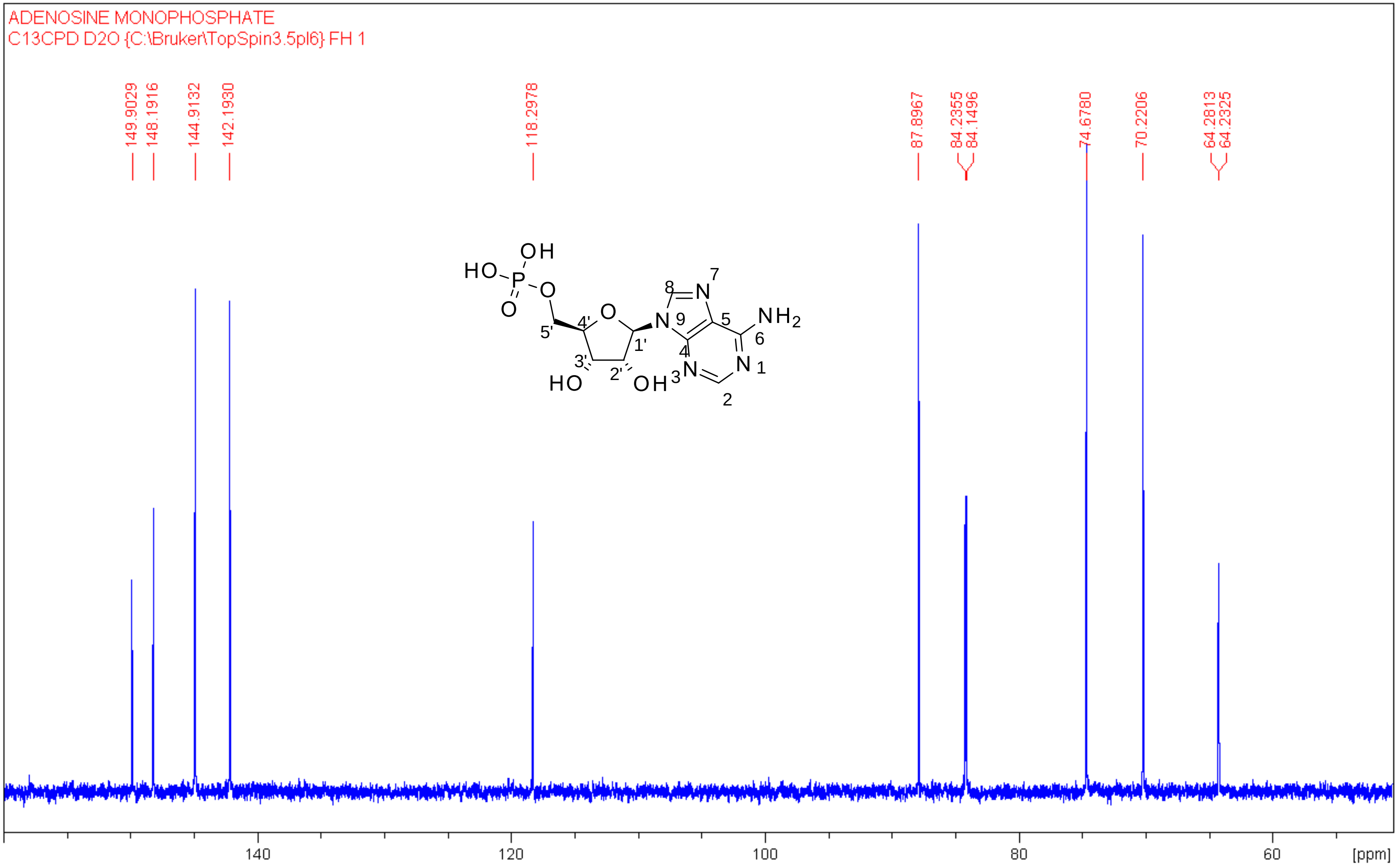

Compound 11. 100 MHz ^{13}C NMR spectrum in D_2O

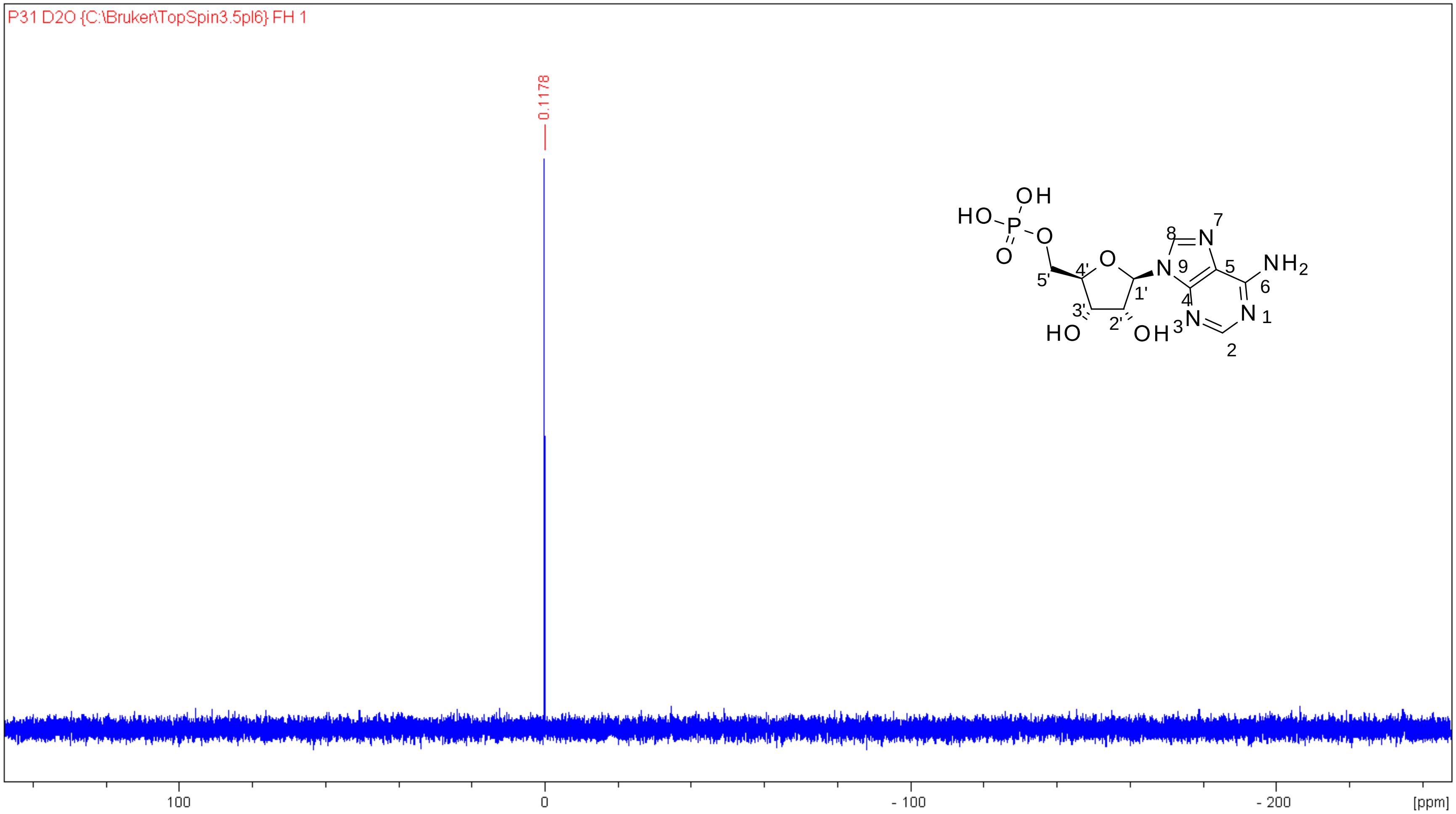

Compound 11. 162 MHz ^{31}PNMR spectrum in D_2O

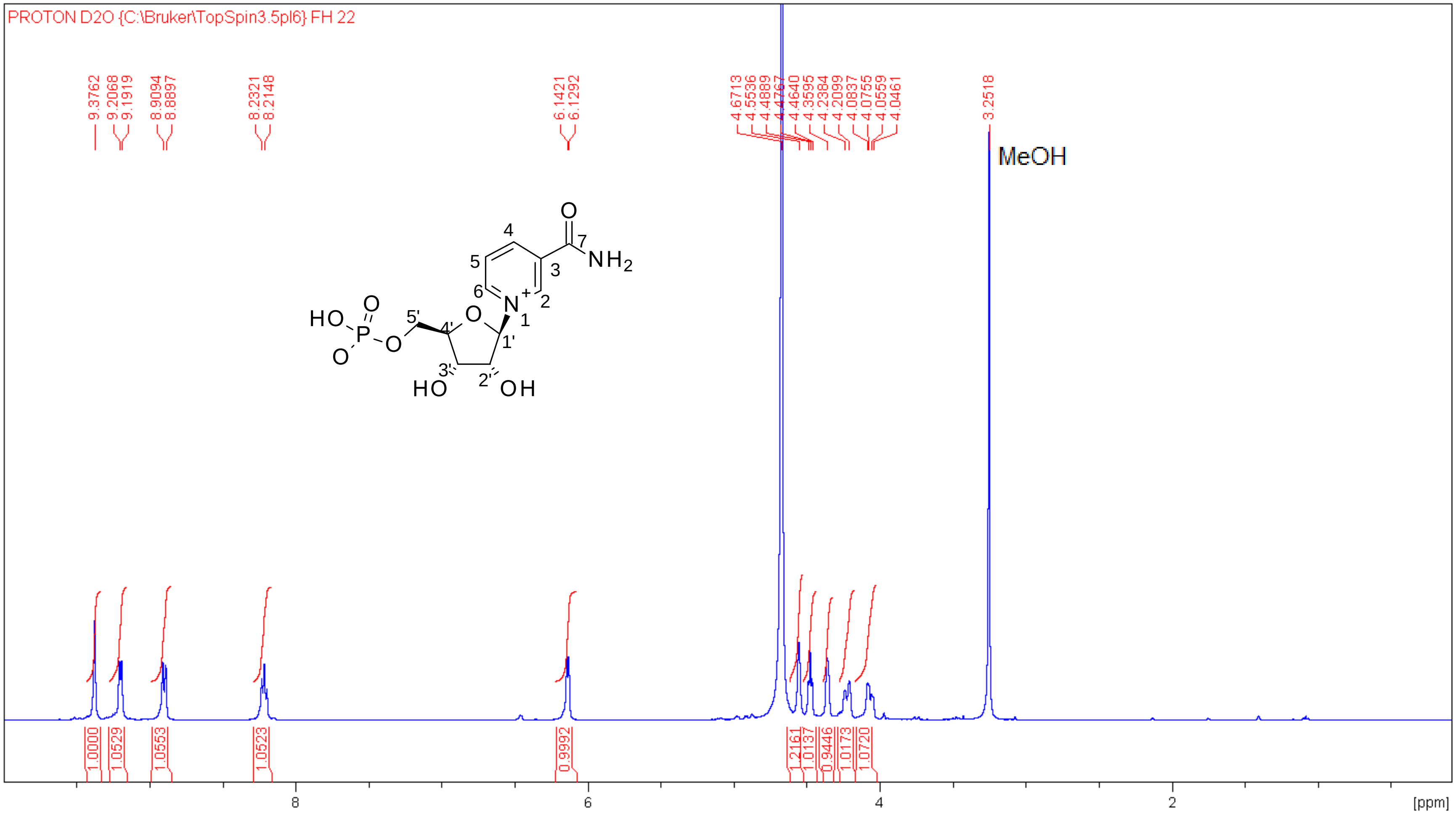

Compound 12. 400 MHz 1H NMR spectrum in D_2O

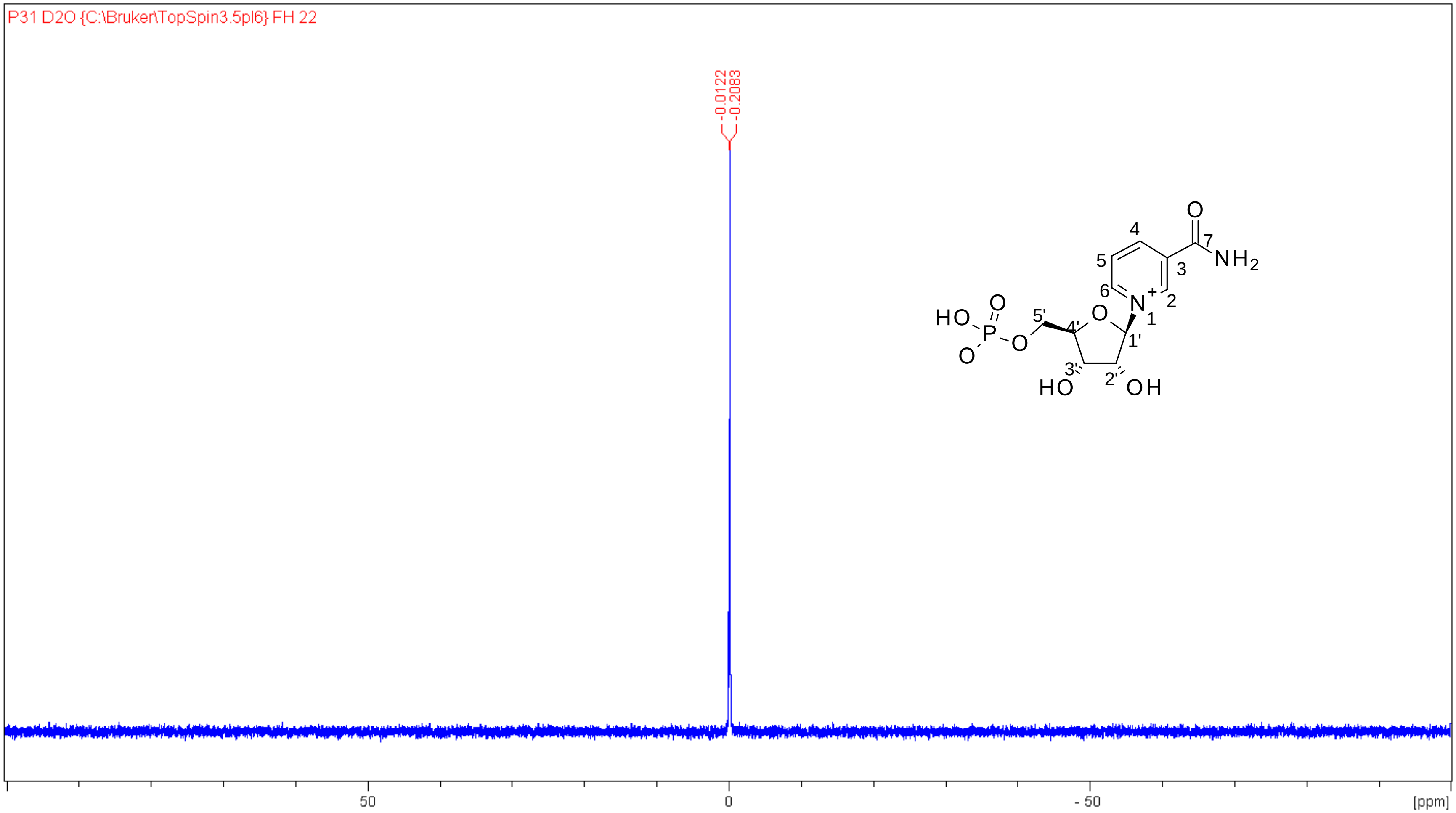

Compound12. 162 MHz ^{31}PNMR spectrum in D_2O

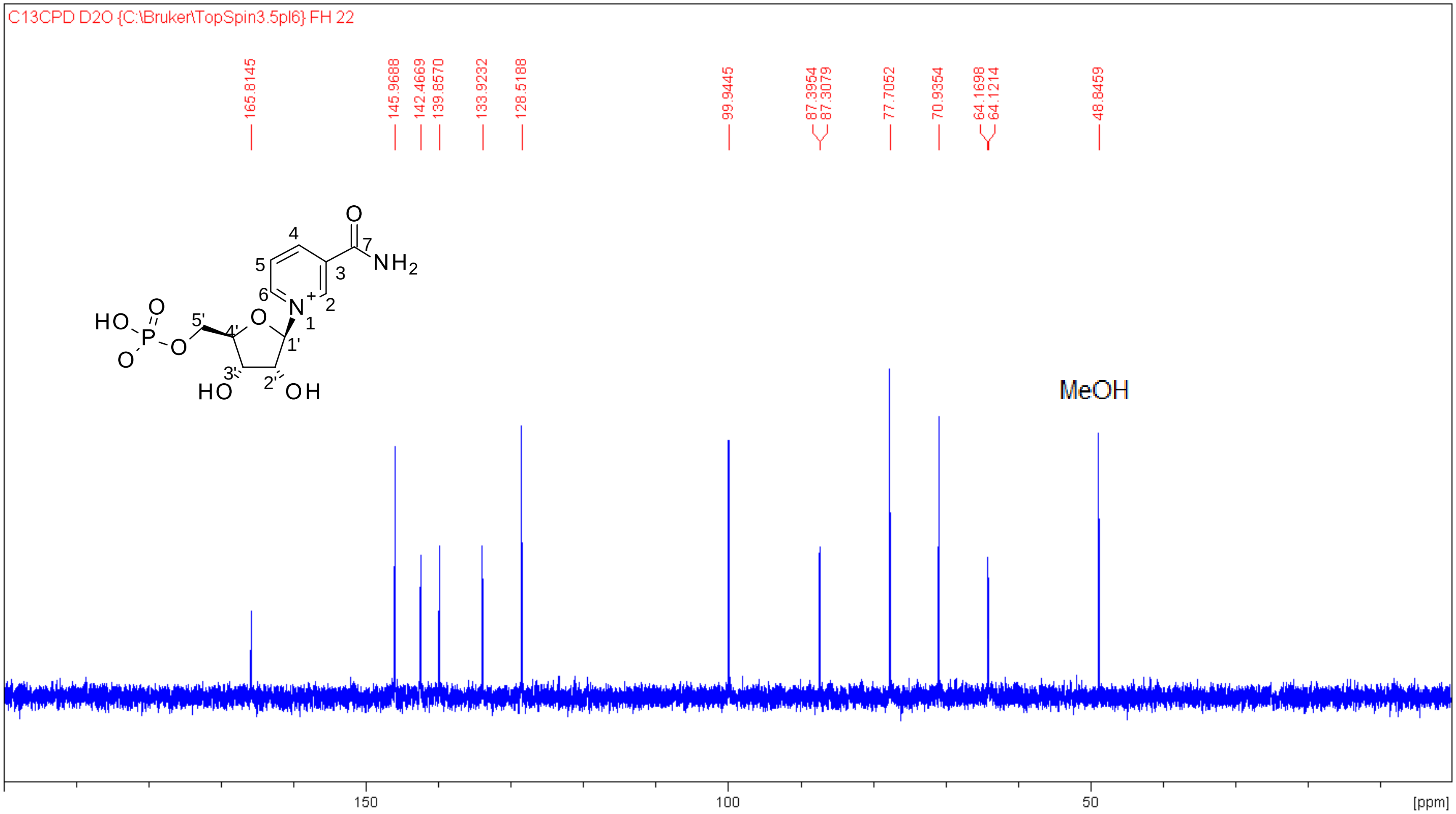

Compound 12. 100 MHz ^{13}C NMR spectrum in D_2O

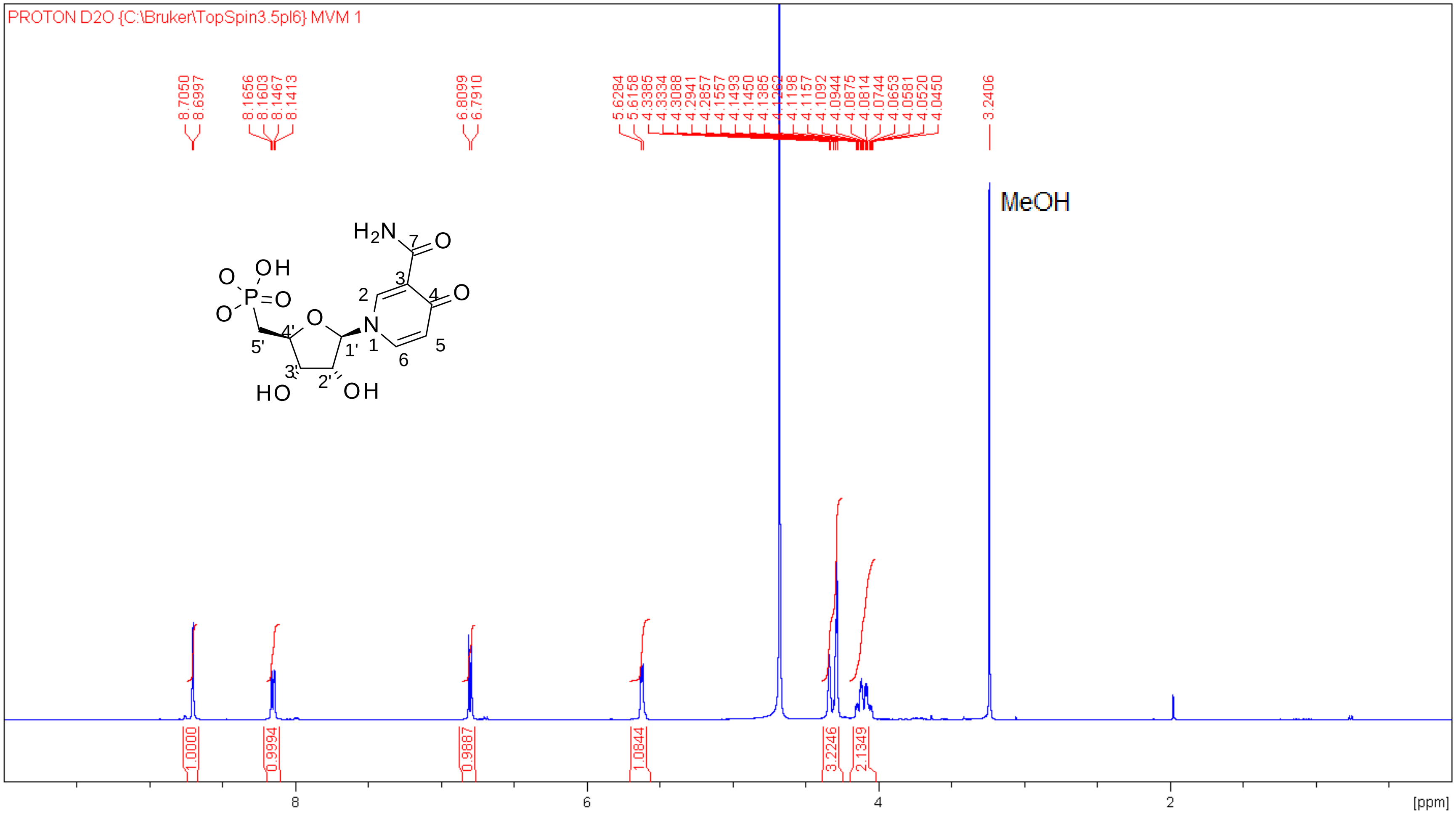

Compound 13. 400 MHz ^{1}H NMR spectrum in D_2O

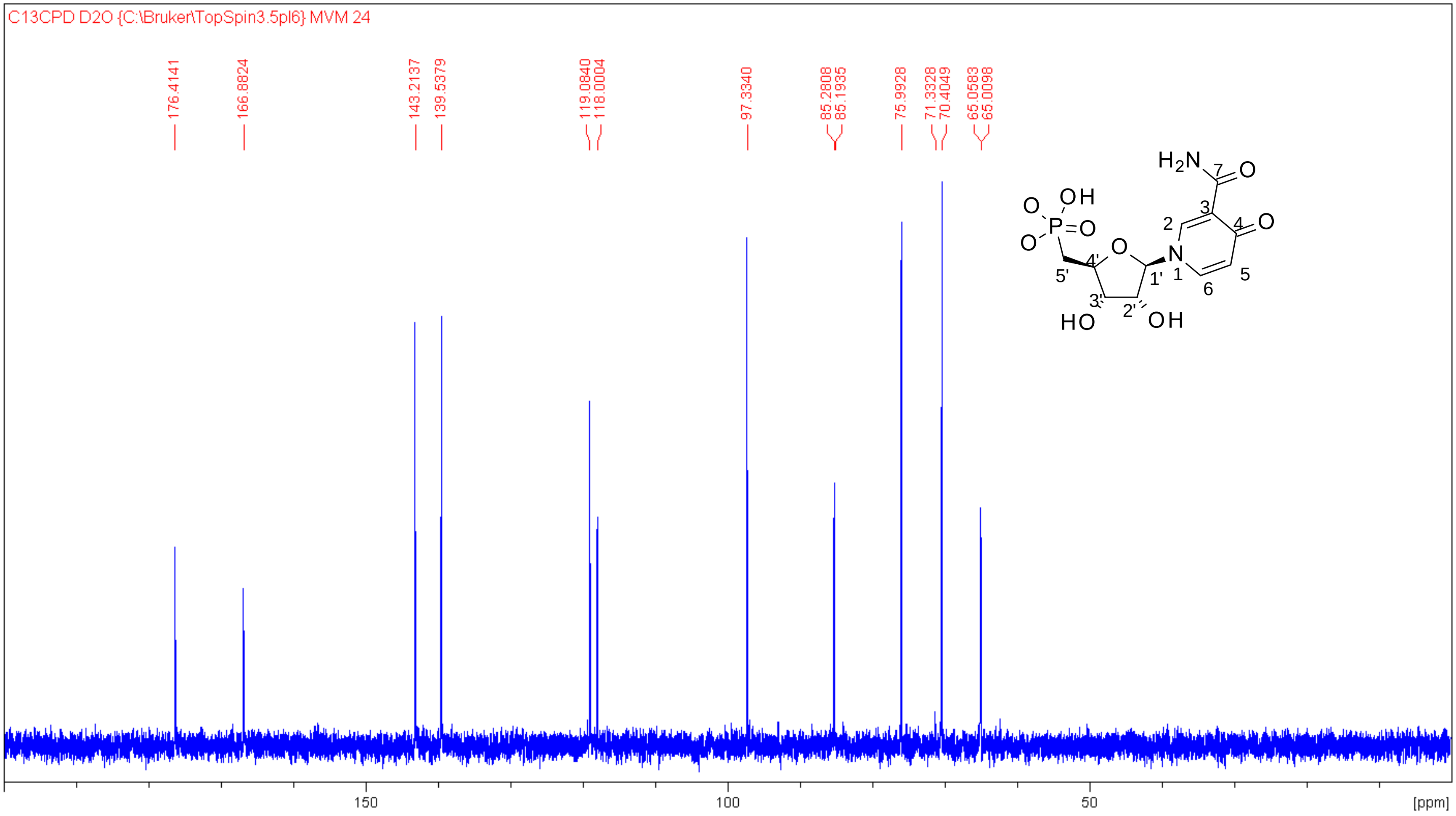

Compound 13. 100 MHz ^{13}C NMR spectrum in D_2O

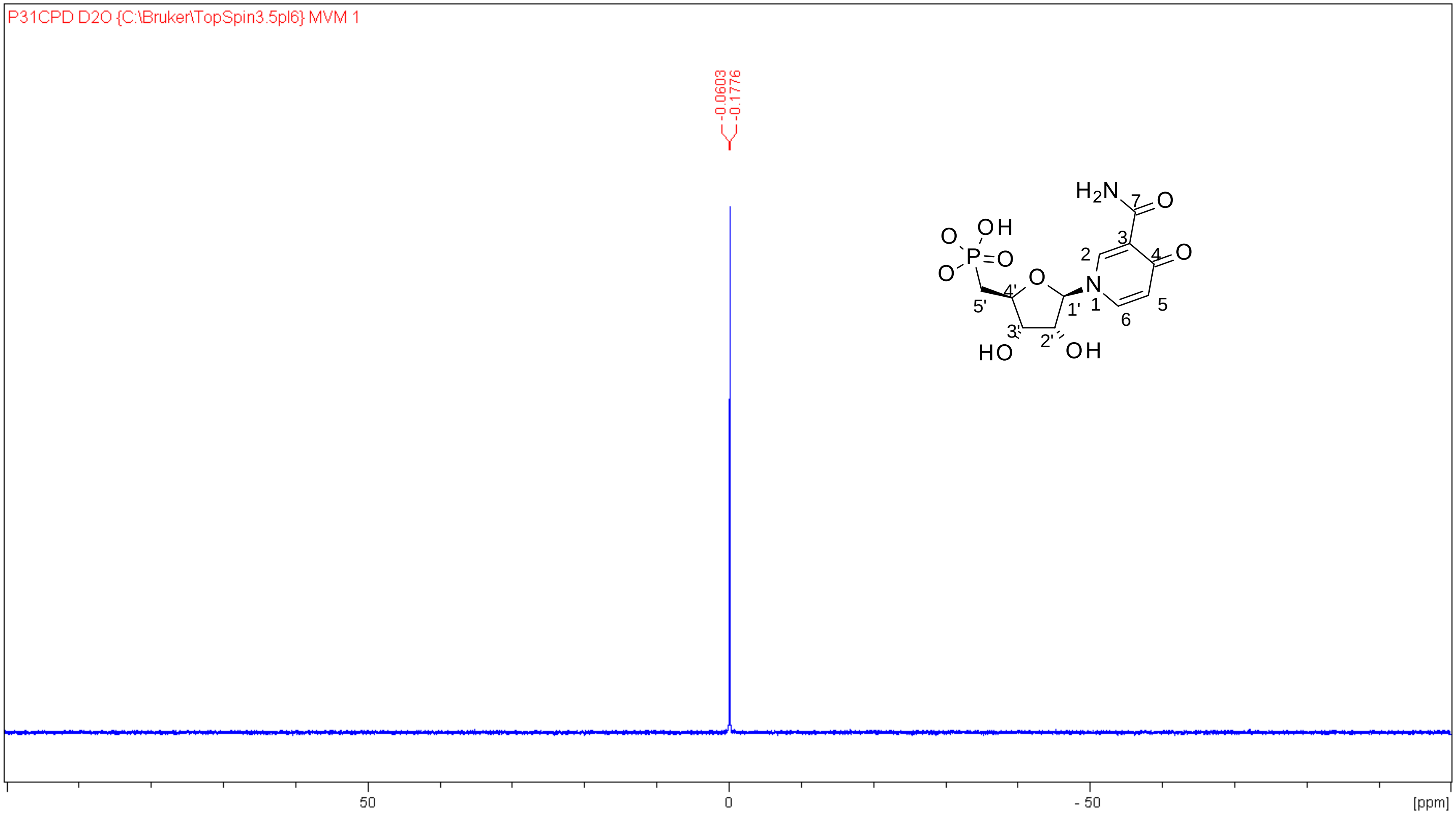

Compound 13. 162 MHz ^{31}P NMR spectrum in D_2O

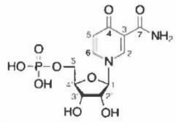

Theoretical M/Z = 351.058793

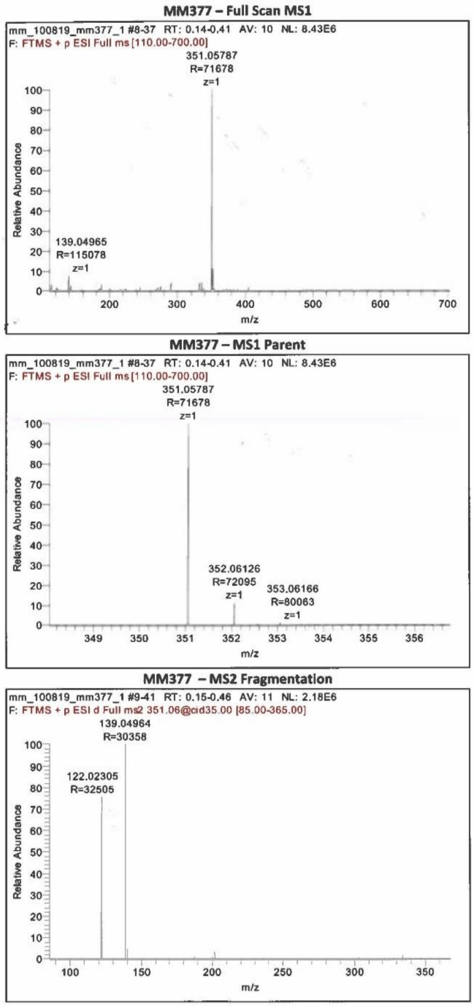

Compound 13. HRMS spectra

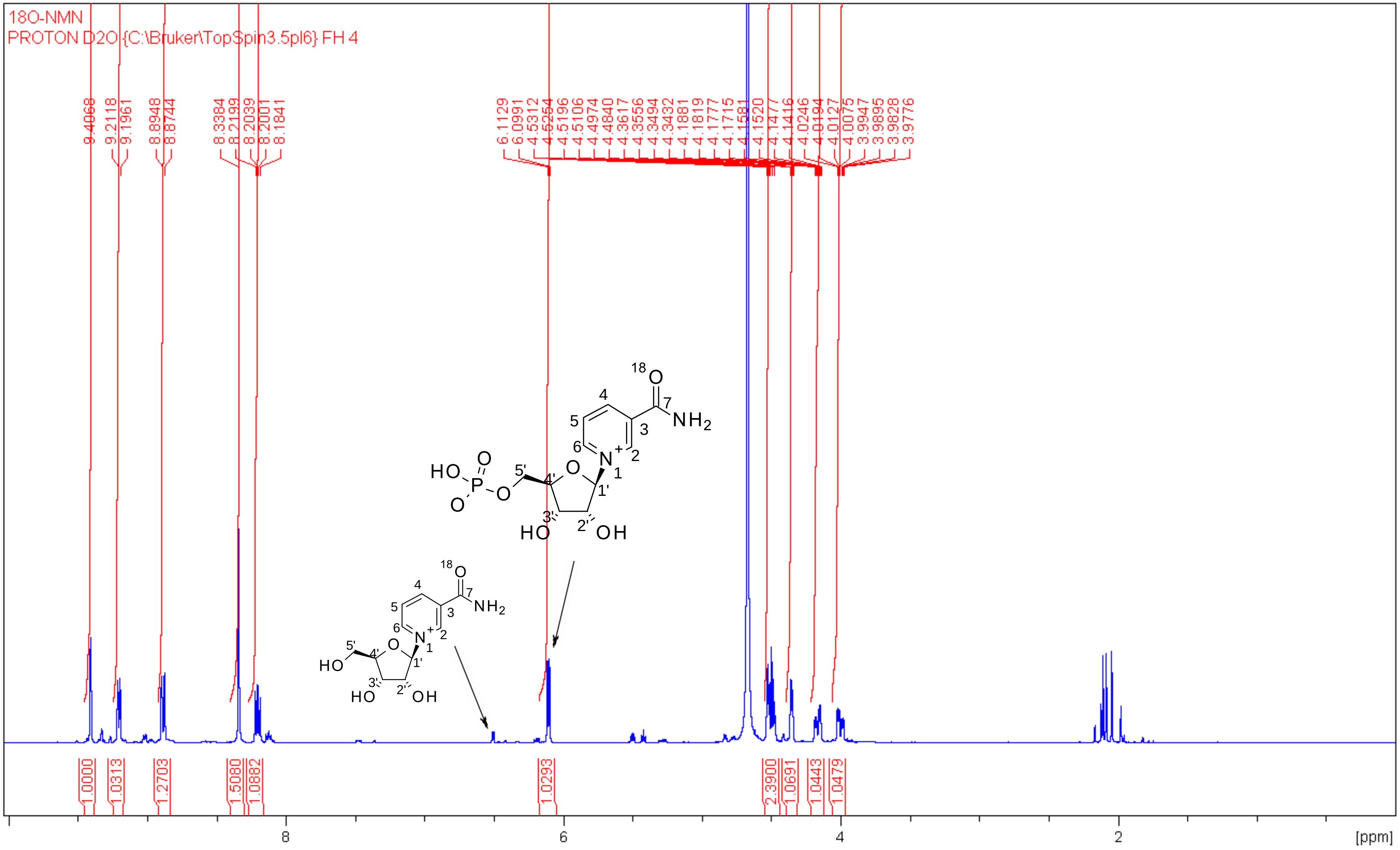

Compound 15. 400 MHz ^{1}H NMR spectrum in D_2O

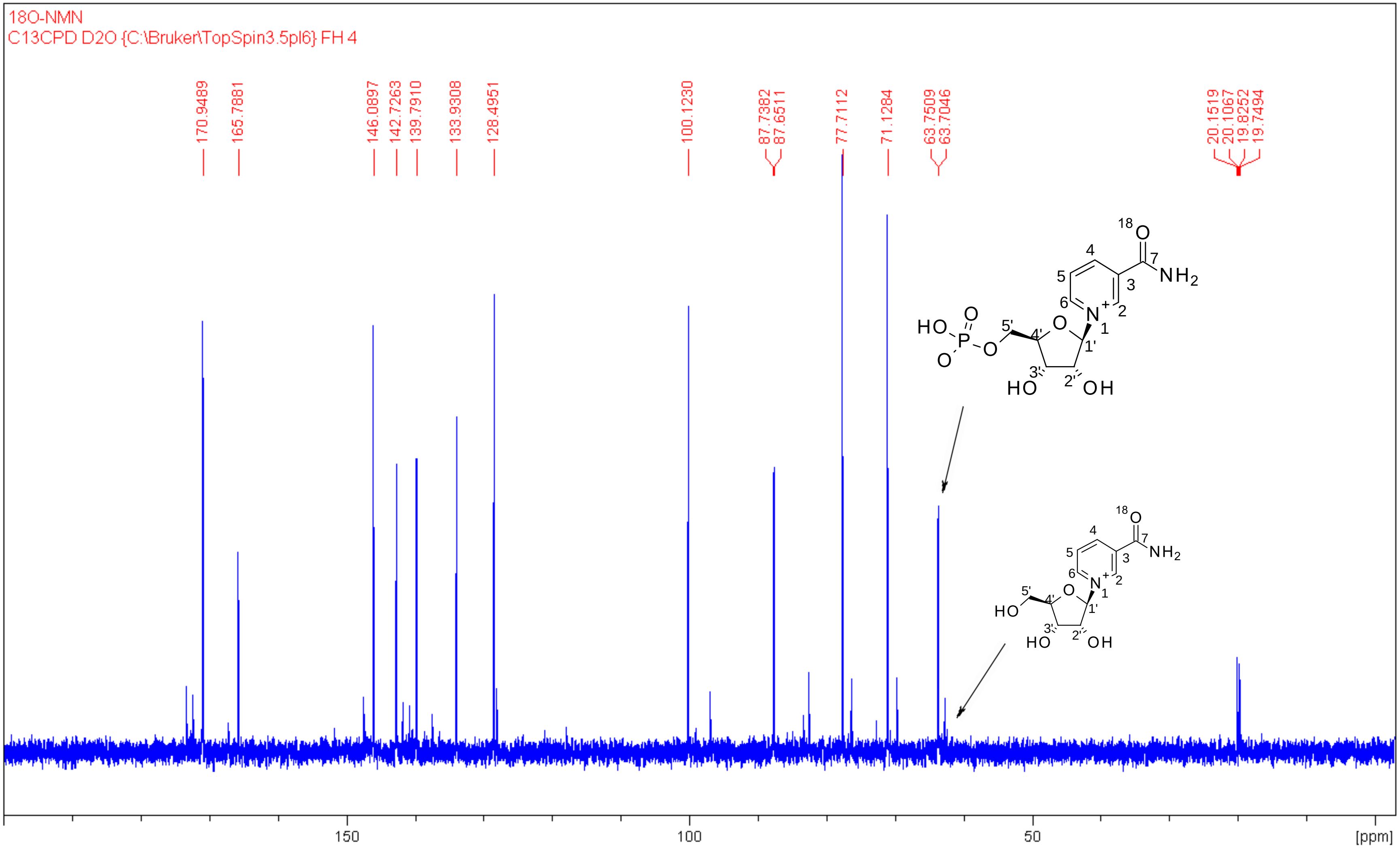

Compound 15. 100 MHz ^{1}H NMR spectrum in D_2O

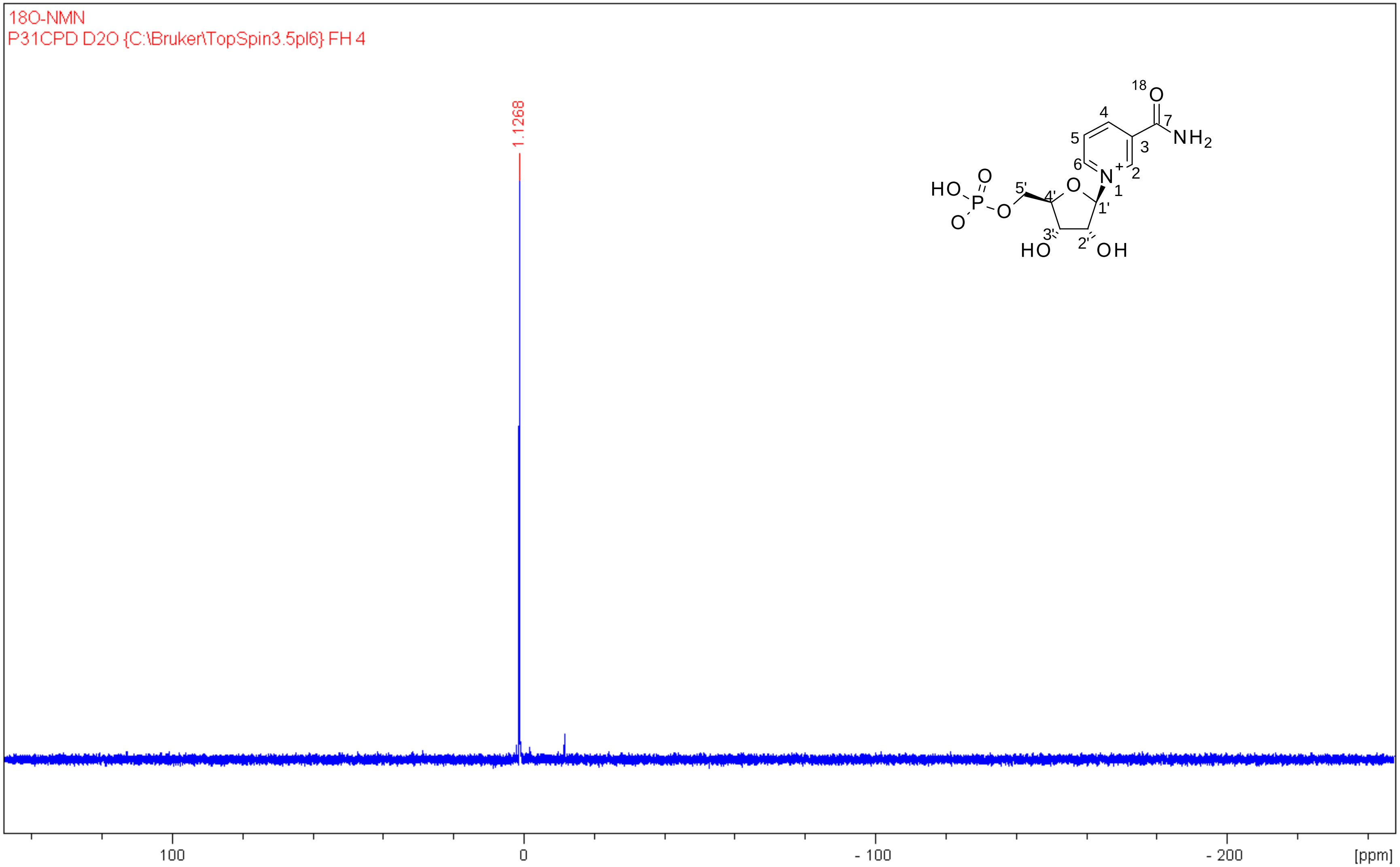

Compound 15. 162 MHz ^{31}P NMR spectrum in D_2O

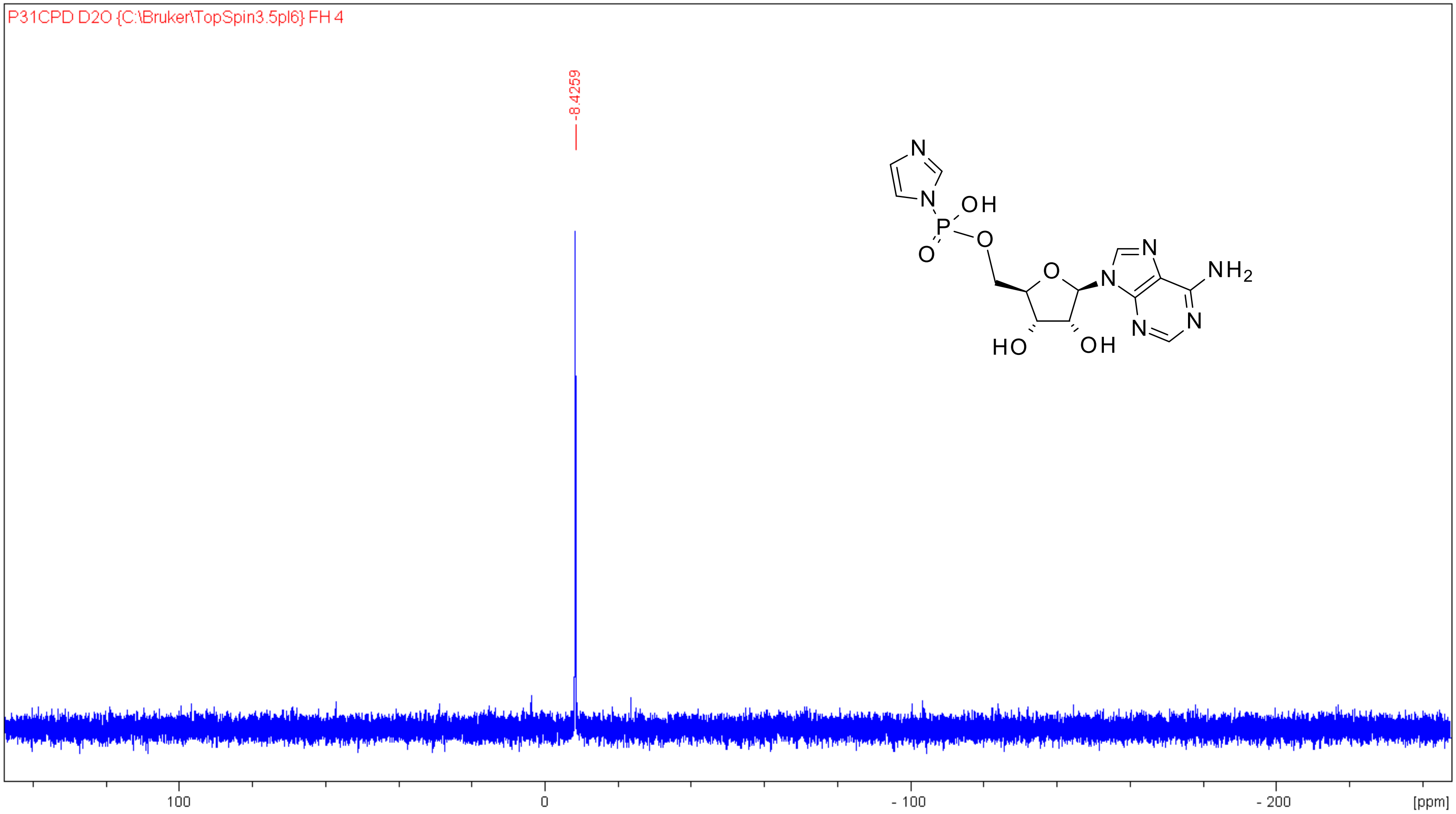

Compound 16. 162 MHz ^{31}P NMR spectrum in D_2O

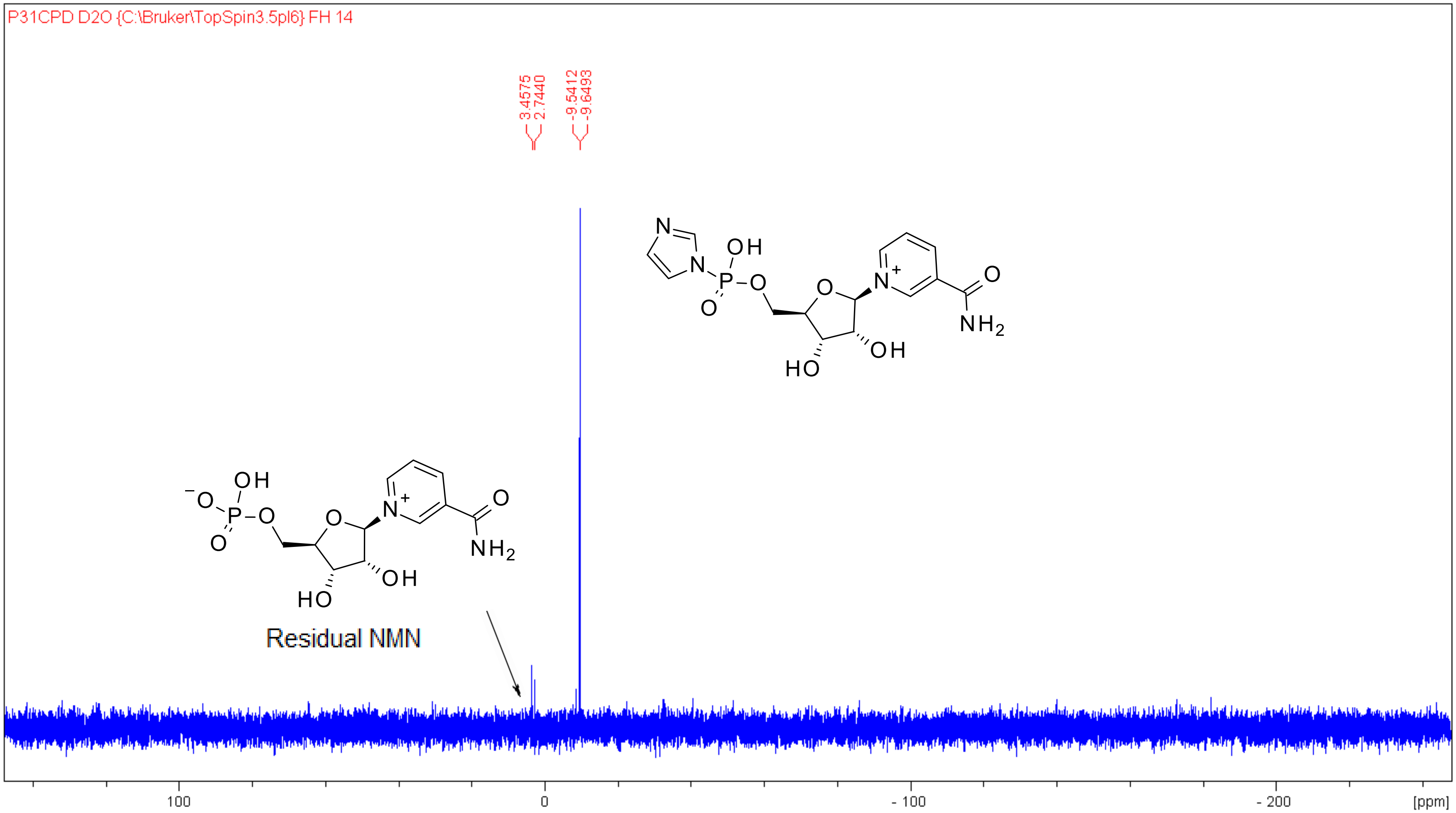

Compound 17. 162 MHz ^{31}P NMR spectrum in D_2O

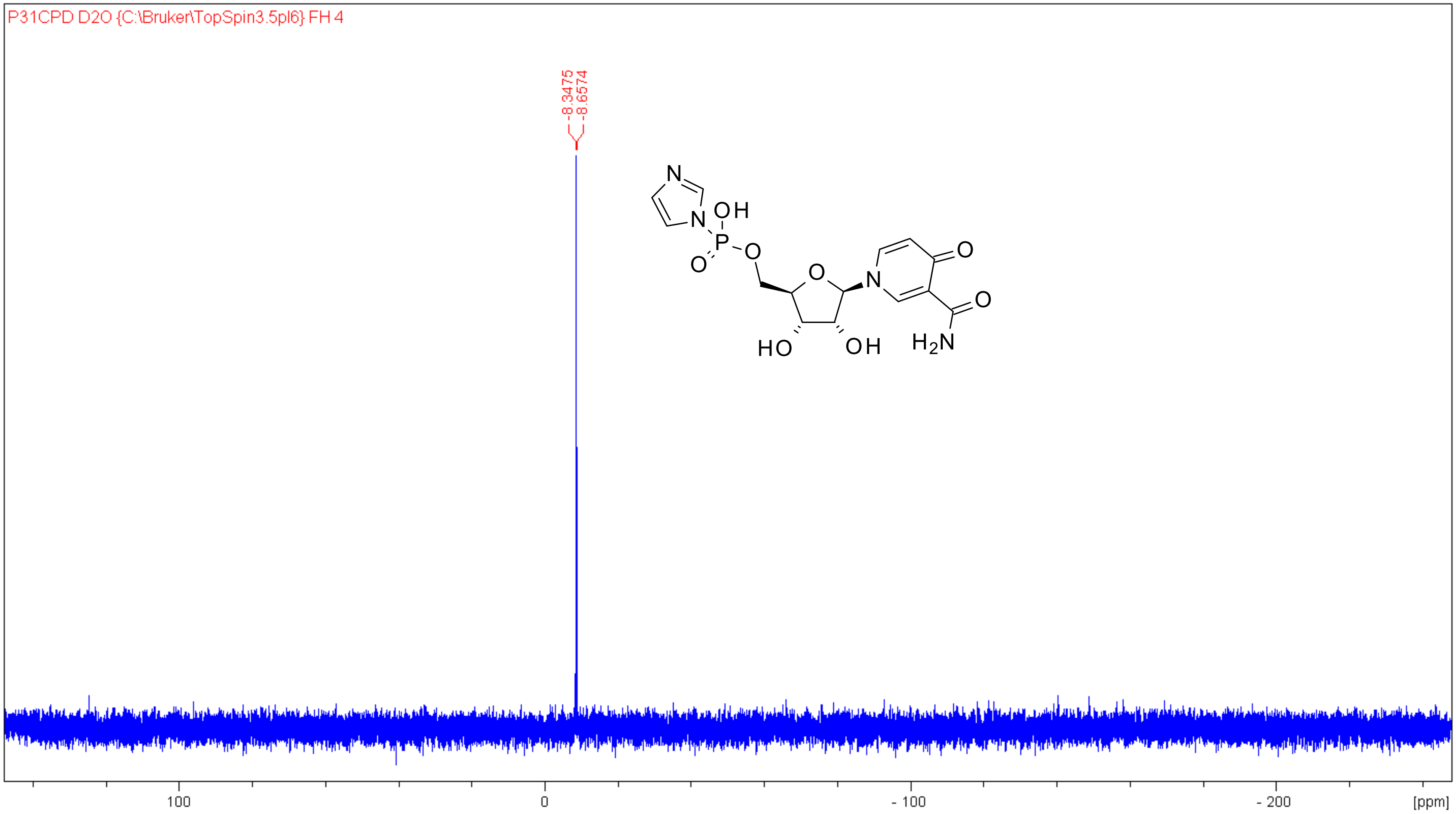

Compound 18. 162 MHz ^{31}P NMR spectrum in D_2O

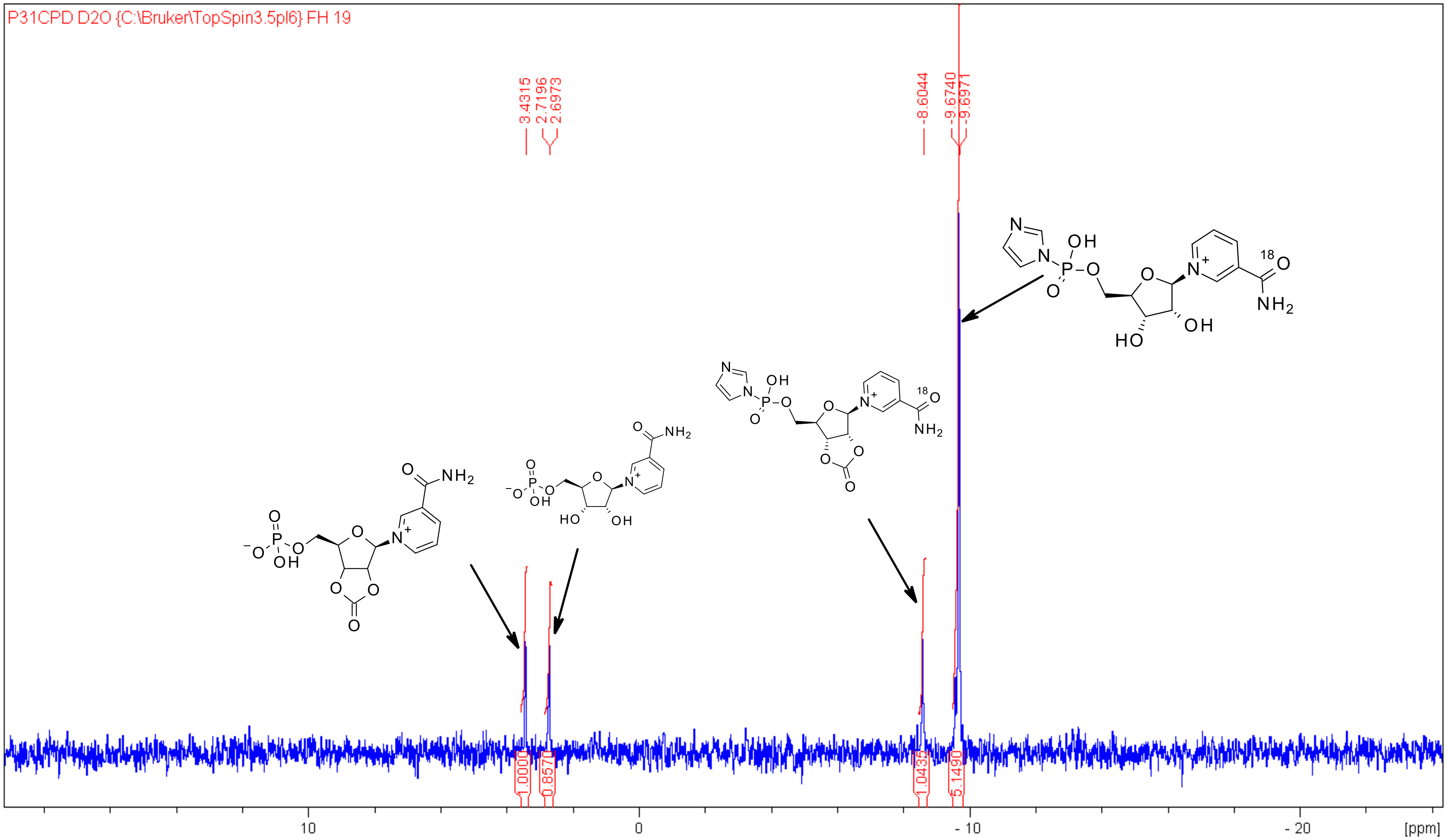

Compound 19. 162 MHz ^{31}P NMR spectrum in D_2O

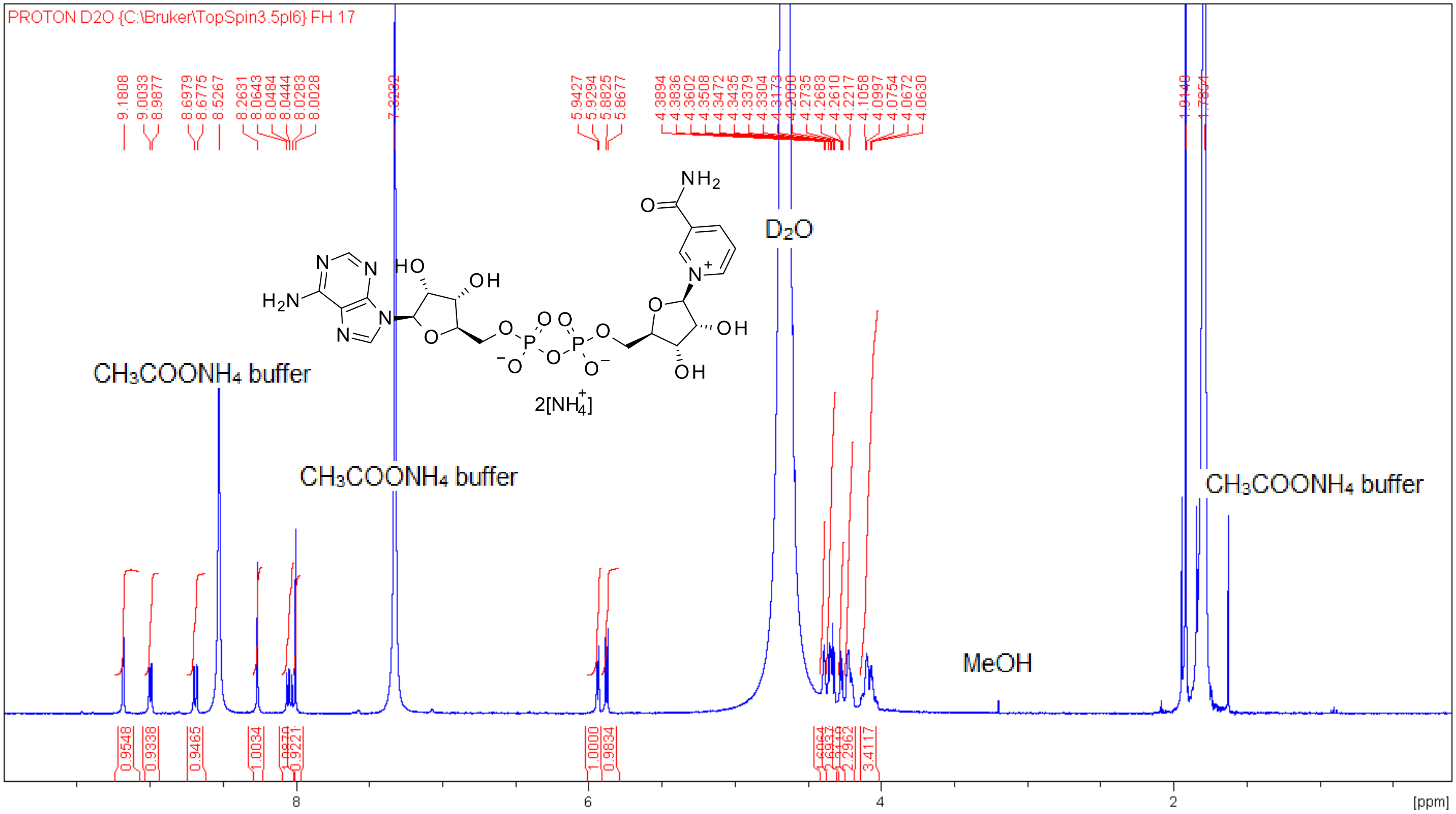

Compound 20. 400 MHz ^{1}H NMR spectrum in D_2O

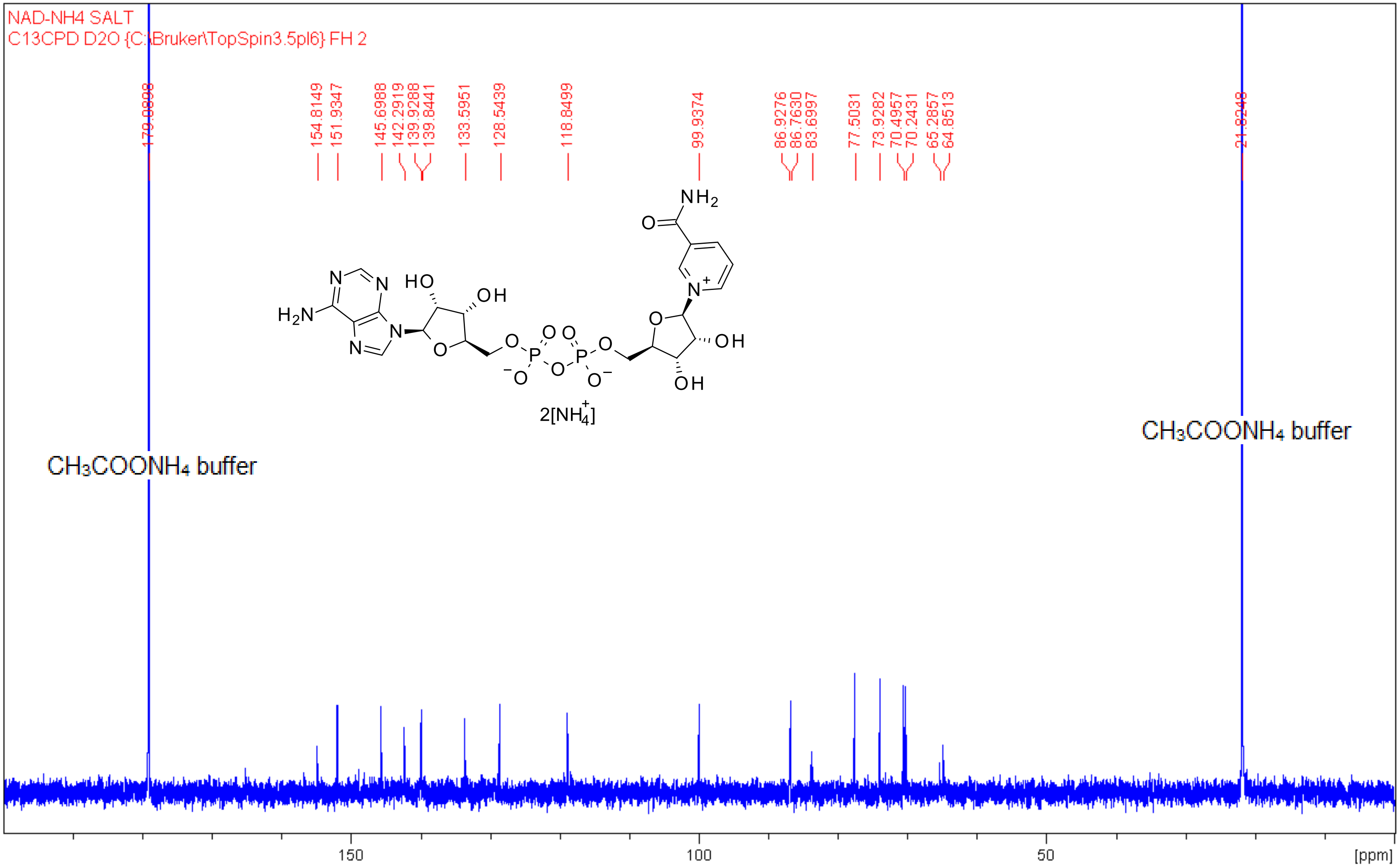

Compound 20. 100 MHz ^{13}C NMR spectrum in D_2O

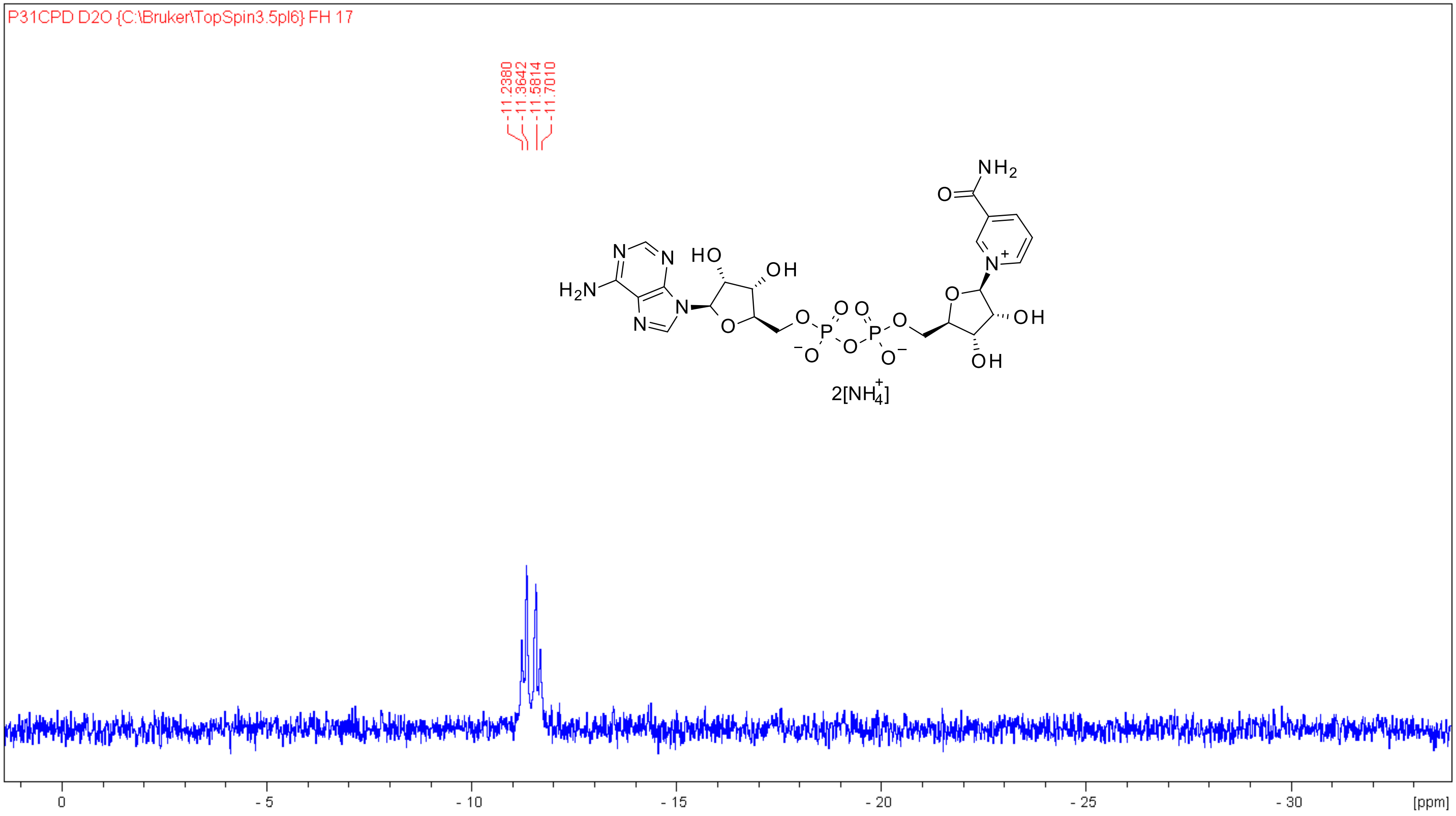

Compound 20. 162MHz ^{31}P NMR spectrum in D_2O

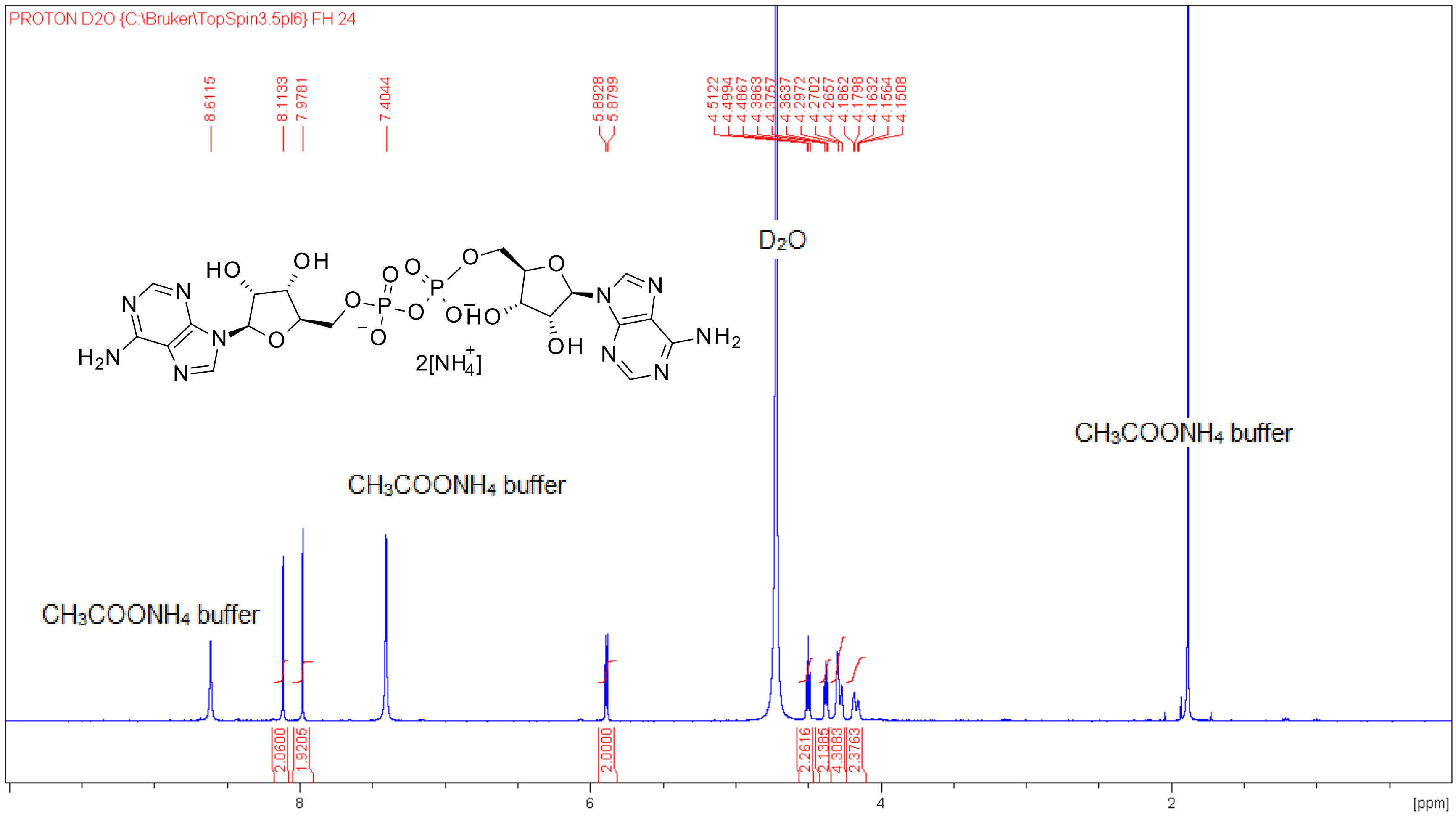

Compound 21. 400 MHz ^{1}H NMR spectrum in D_2O

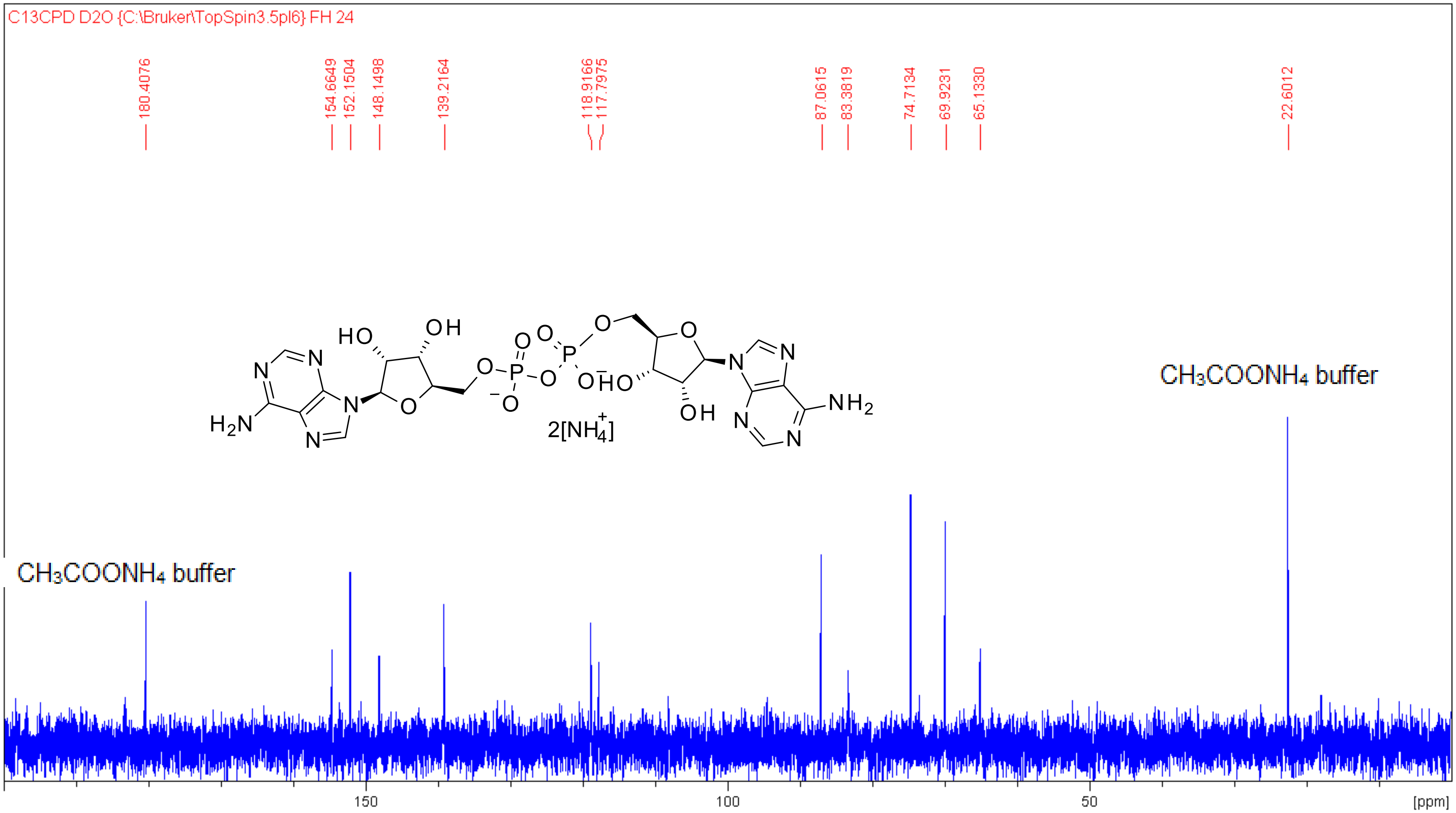

Compound 21. 100 MHz ^{13}C NMR spectrum in D_2O

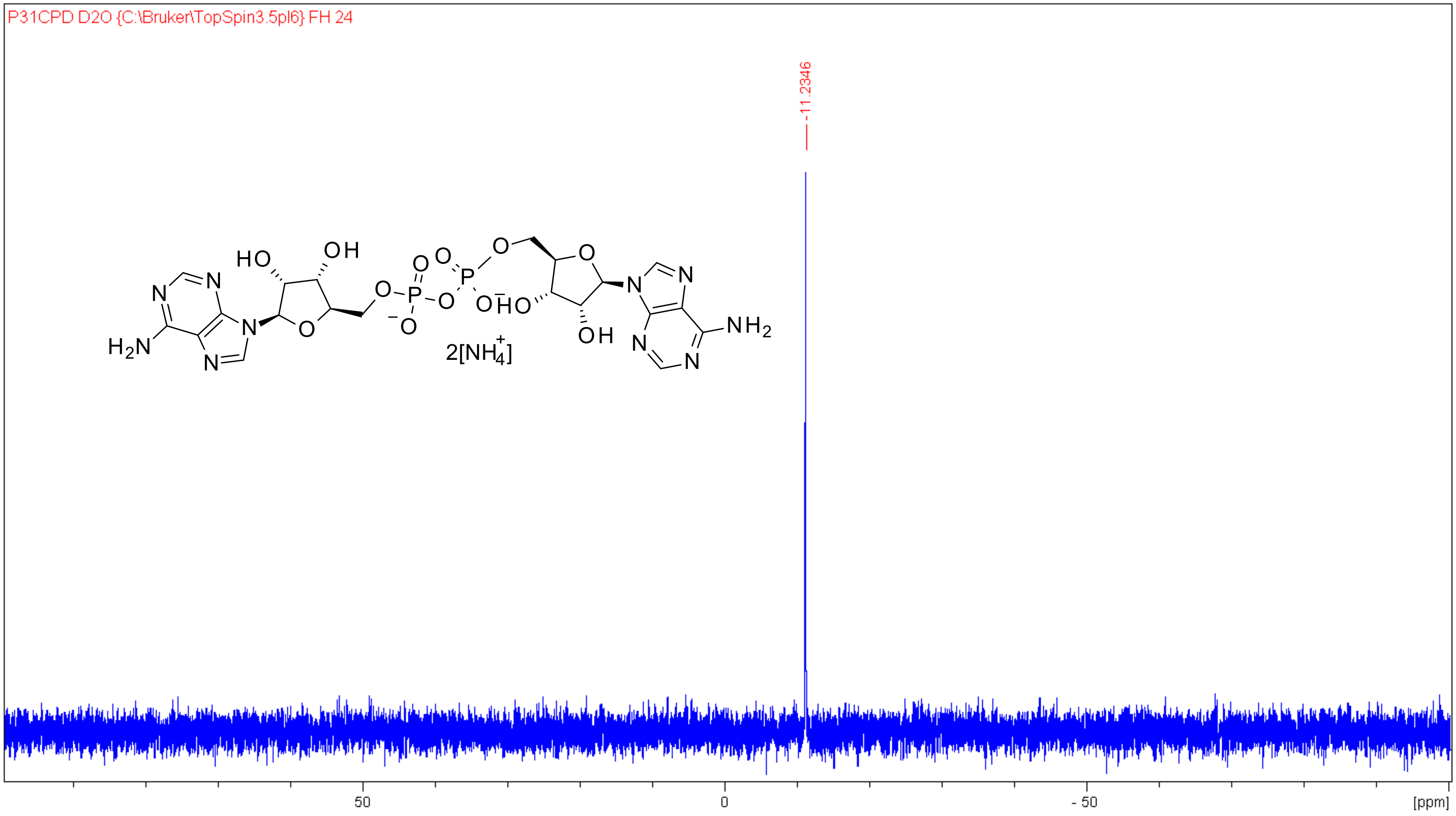

Compound 21. 162MHz ^{31}P NMR spectrum in D_2O

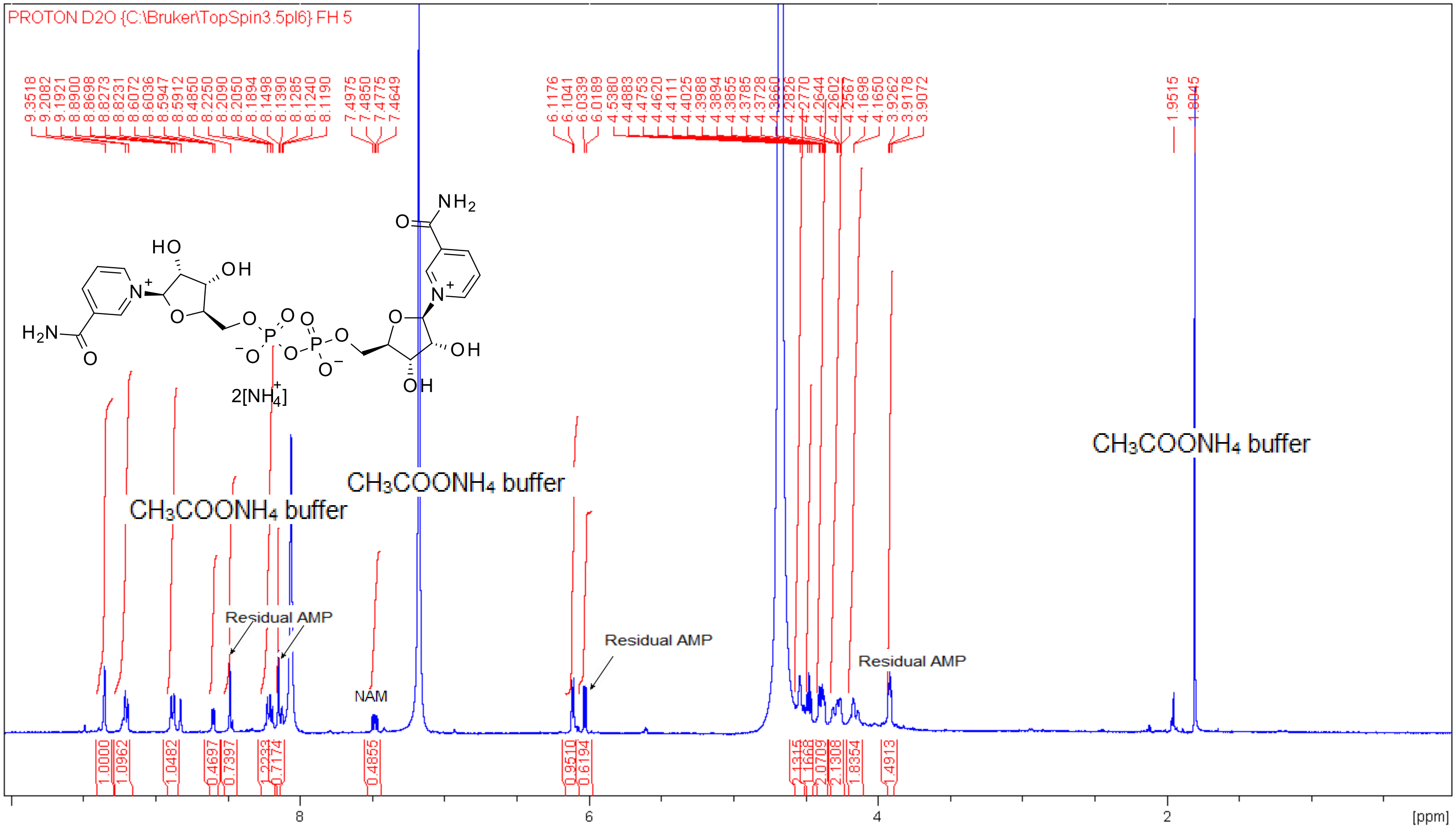

Compound 22. 400 MHz ^{1}H NMR spectrum in D_2O

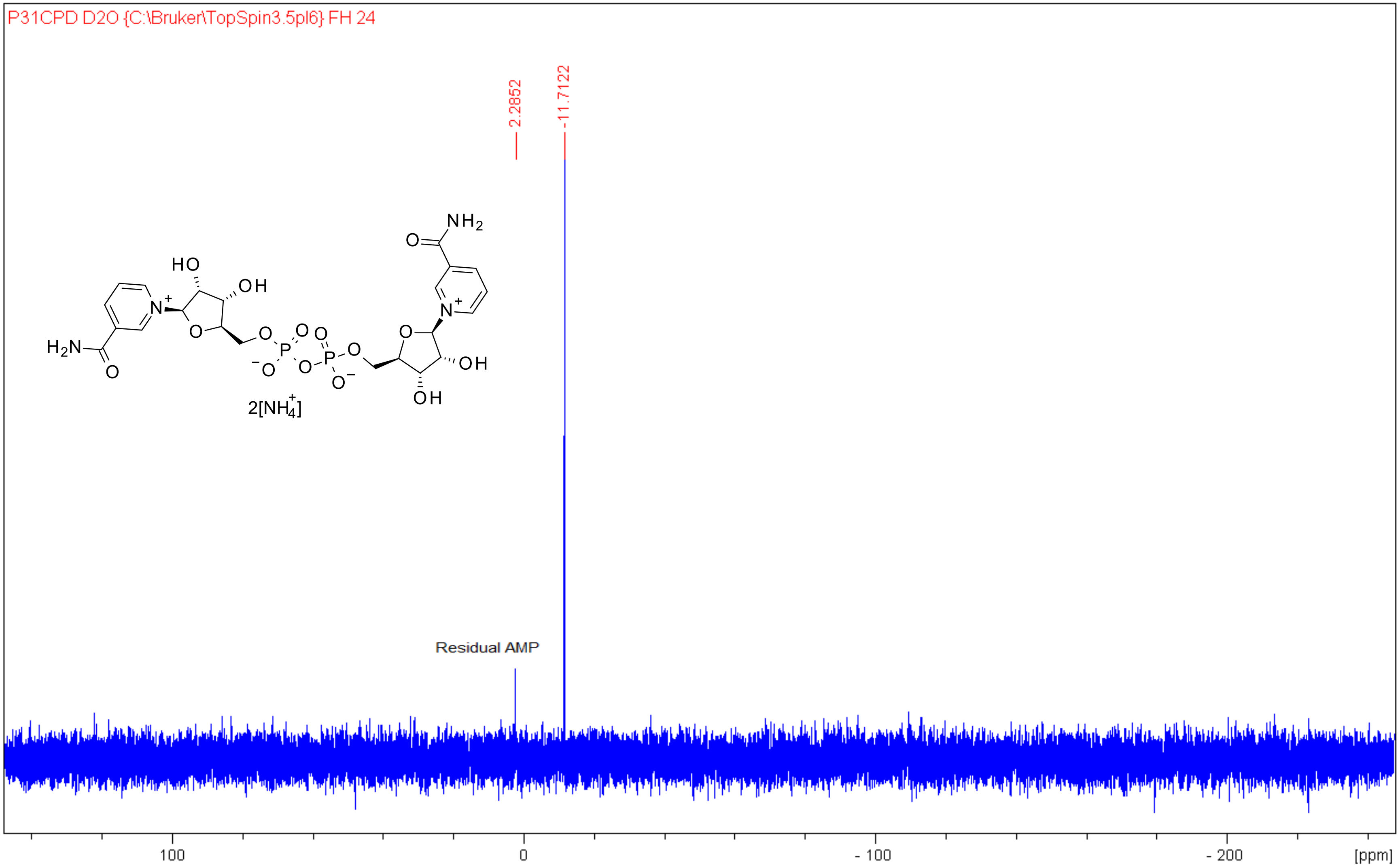

Compound 22. 162 MHz ^{31}P NMR spectrum in D_2O

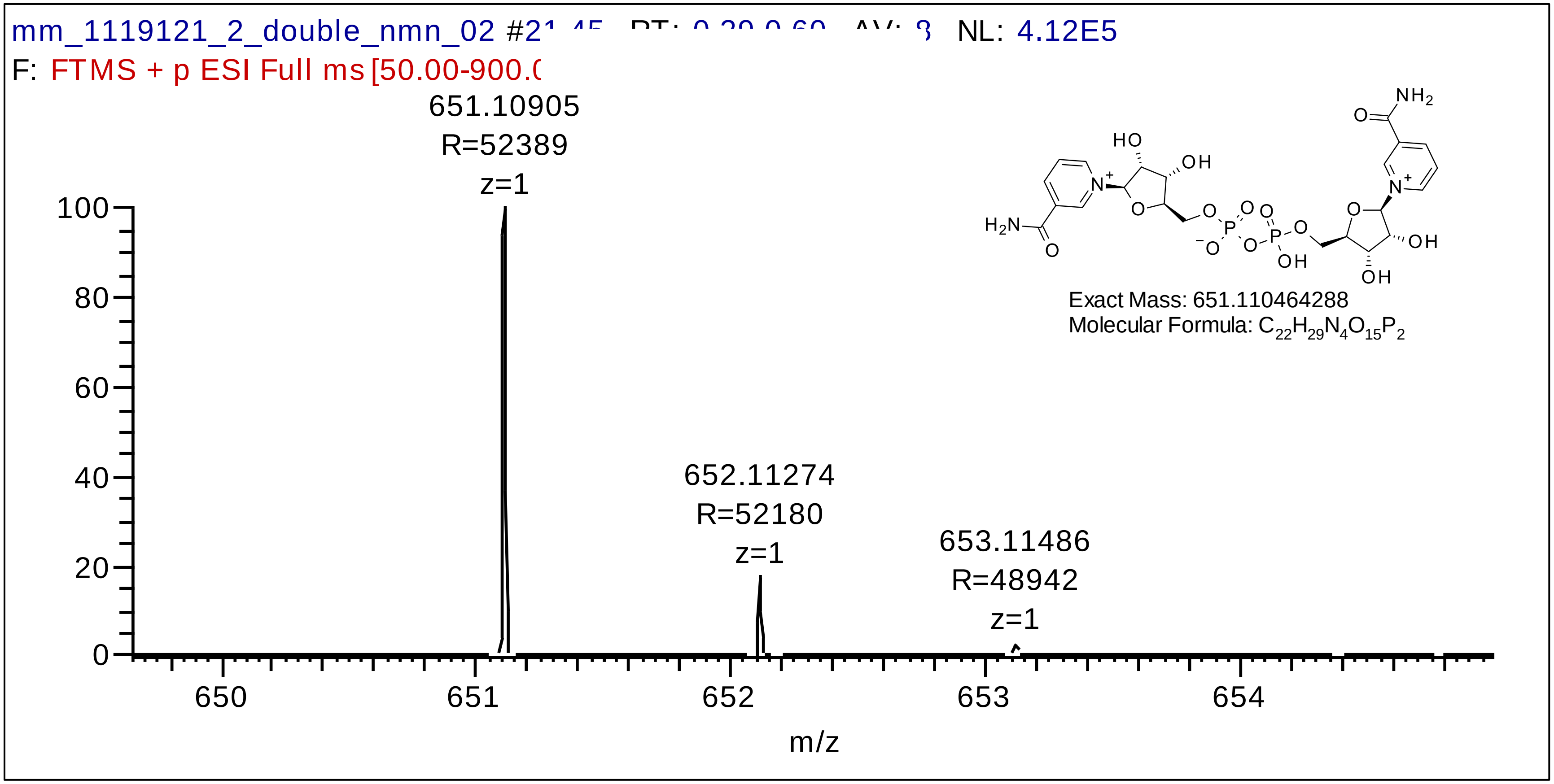

Compound 22. HRMS spectra

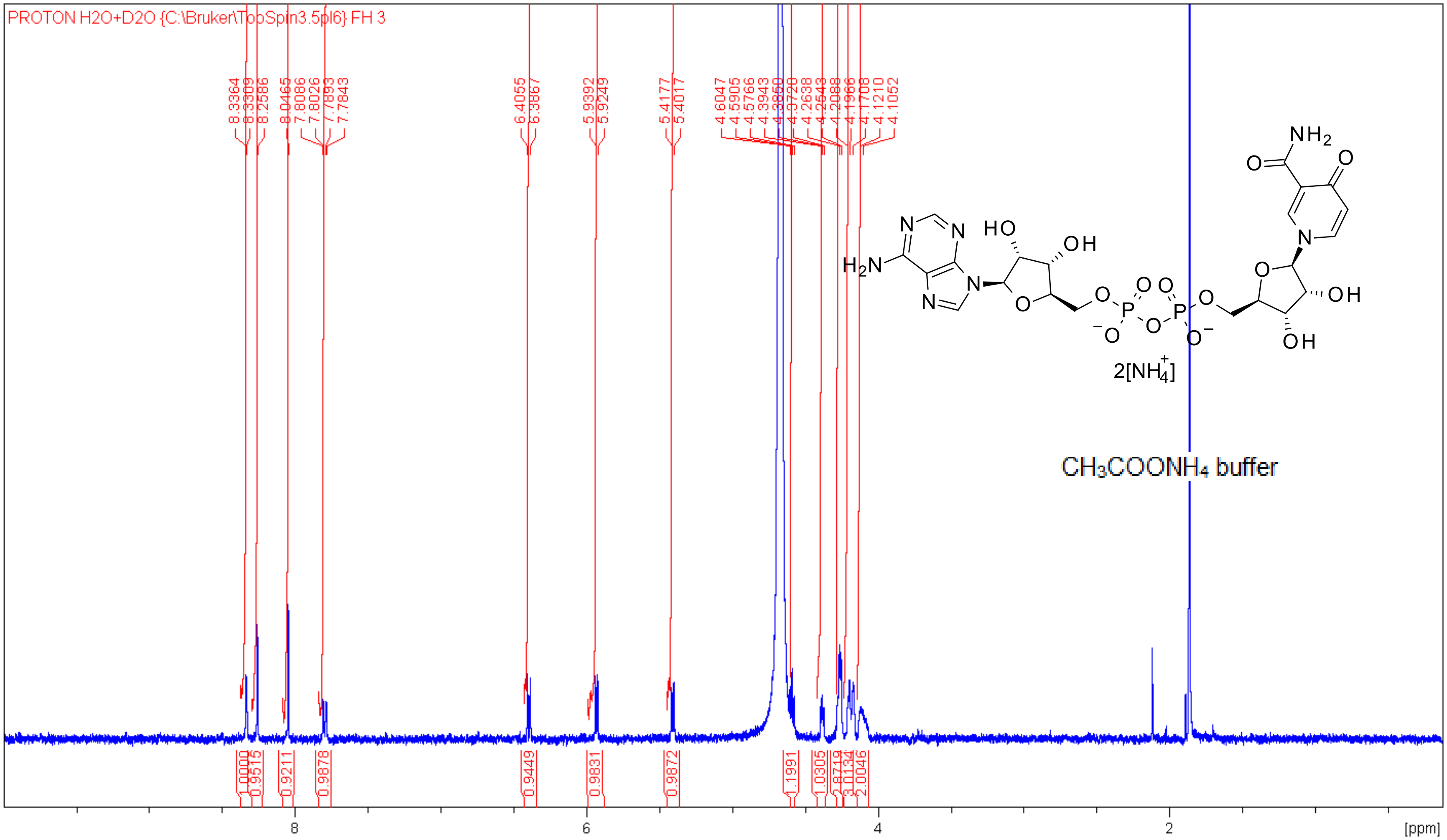

Compound 23. 400 MHz ^{1}H NMR spectrum in D_2O

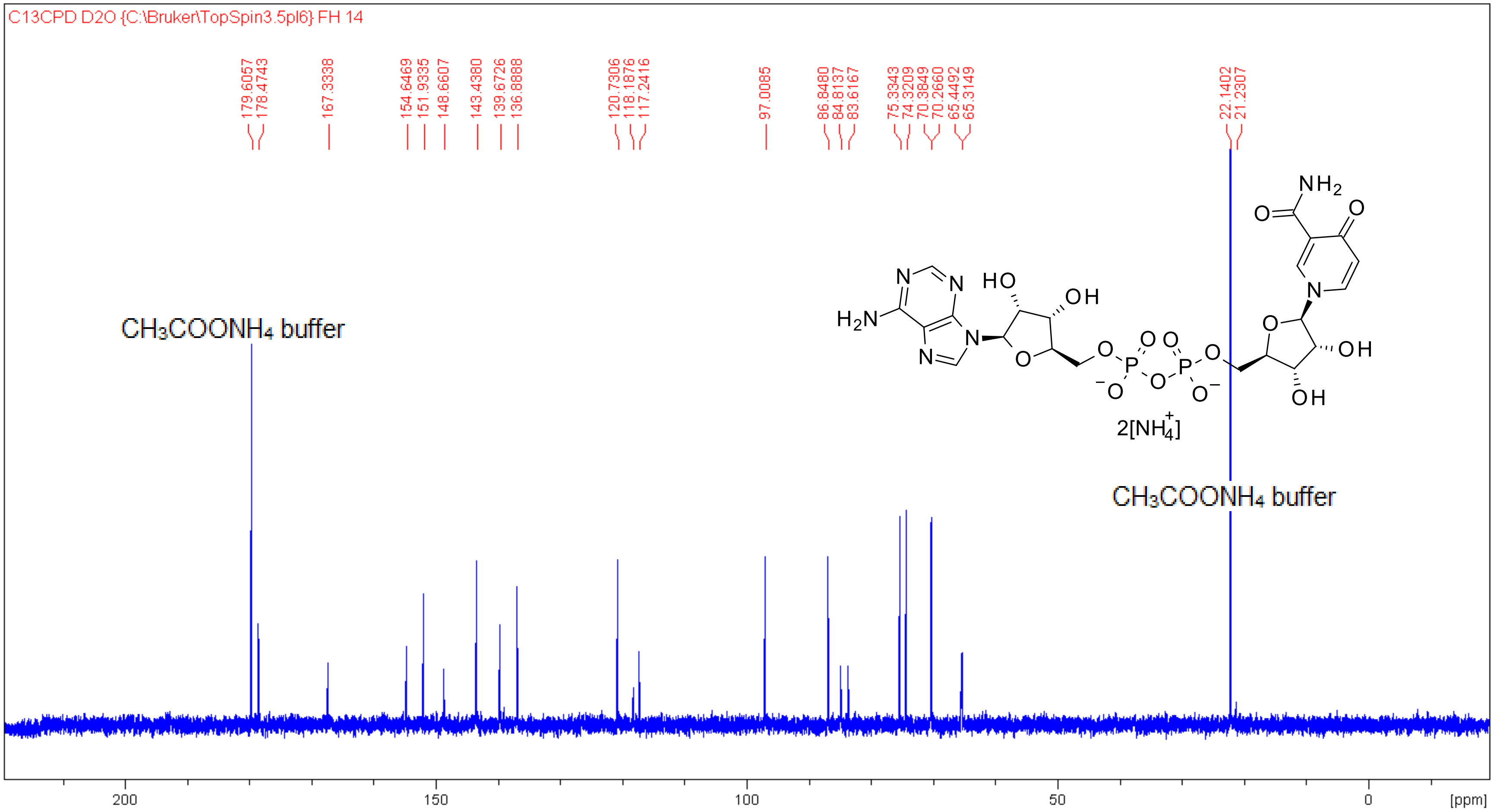

Compound 23. 100 MHz ^{13}C NMR spectrum in D_2O

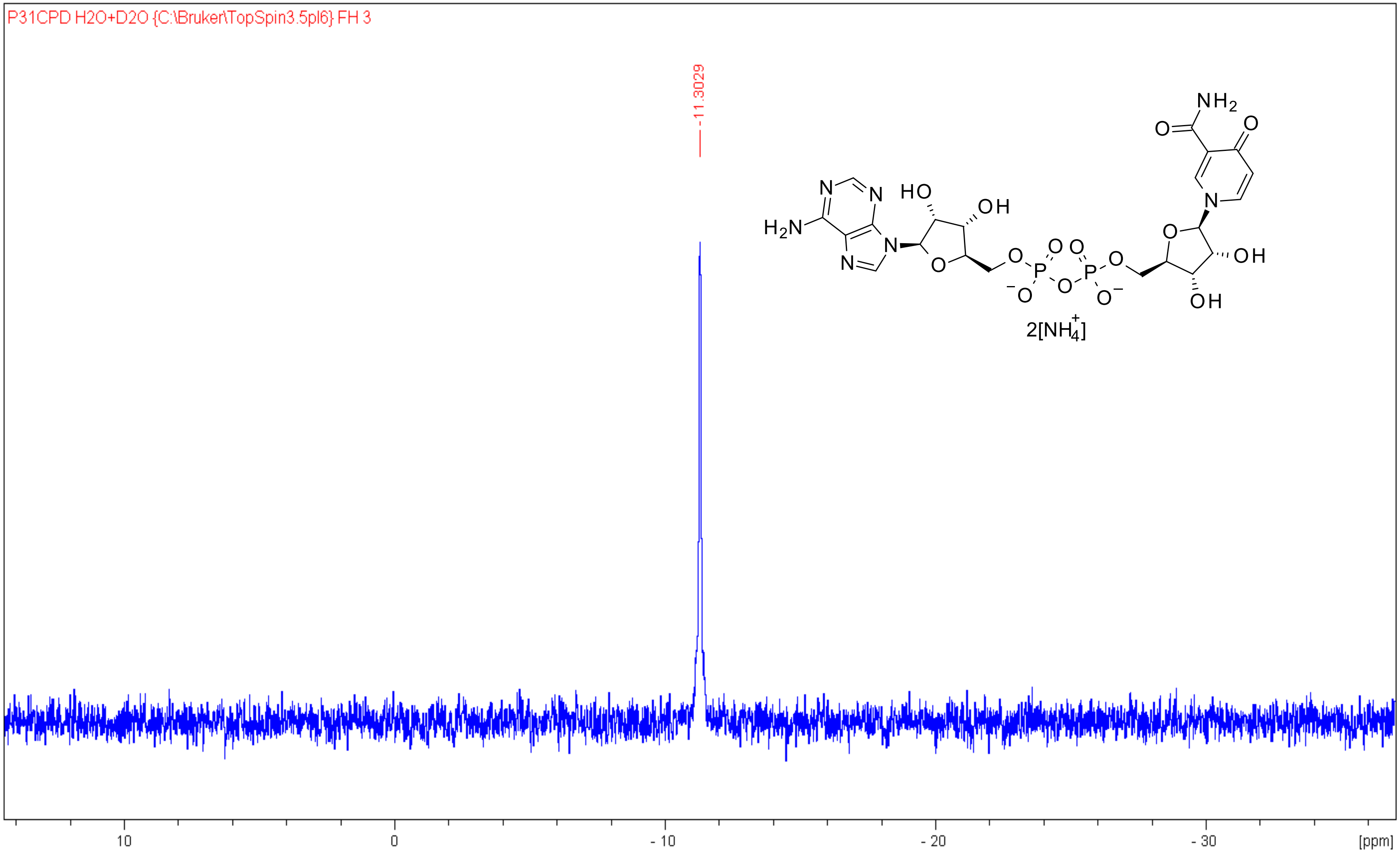

Compound 23. 162 MHz ^{31}P NMR spectrum in D_2O

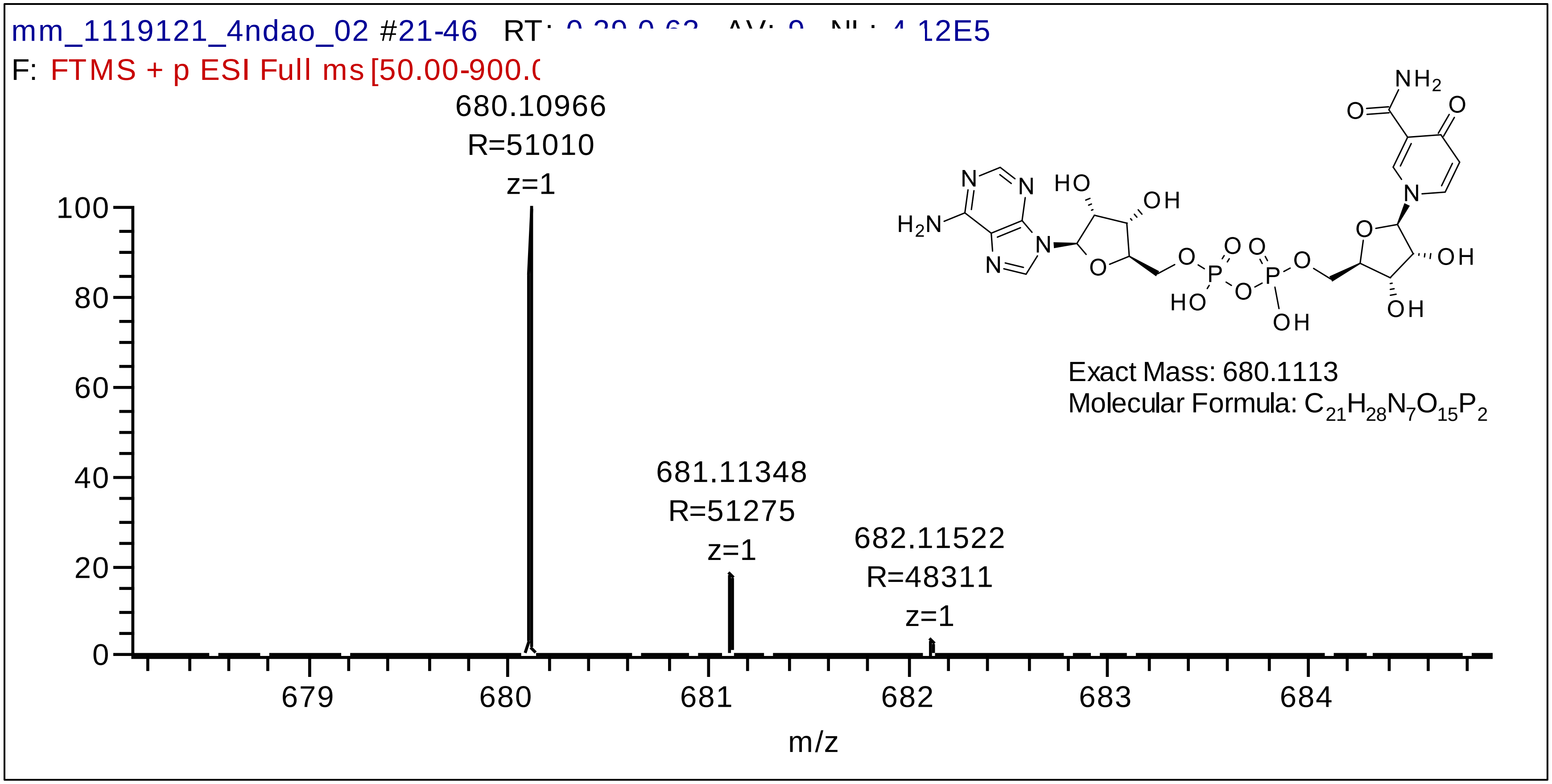

Compound 23. HRMS Spectra

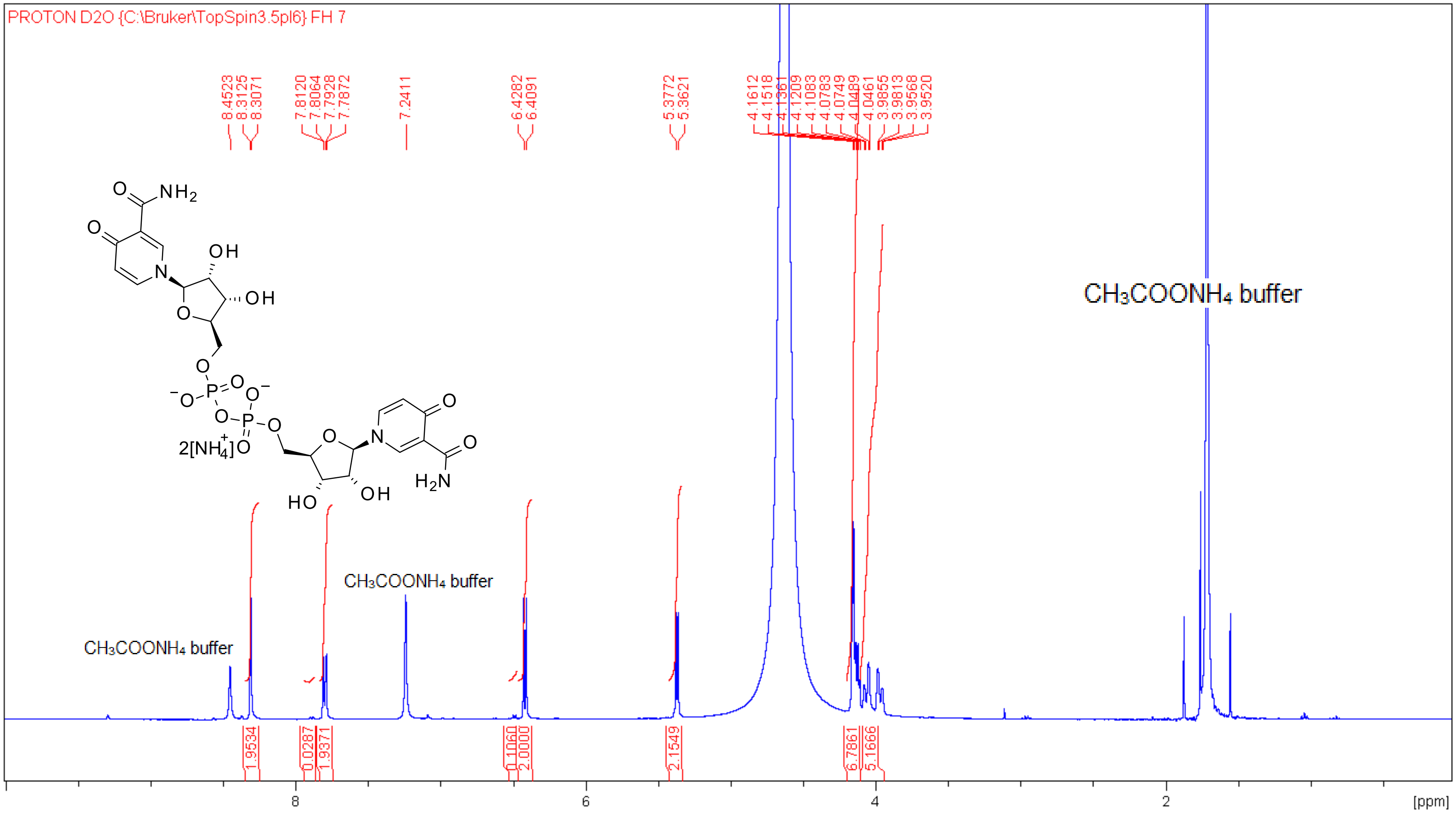

Compound 24. 400 MHz ^{1}H NMR spectrum in D_2O

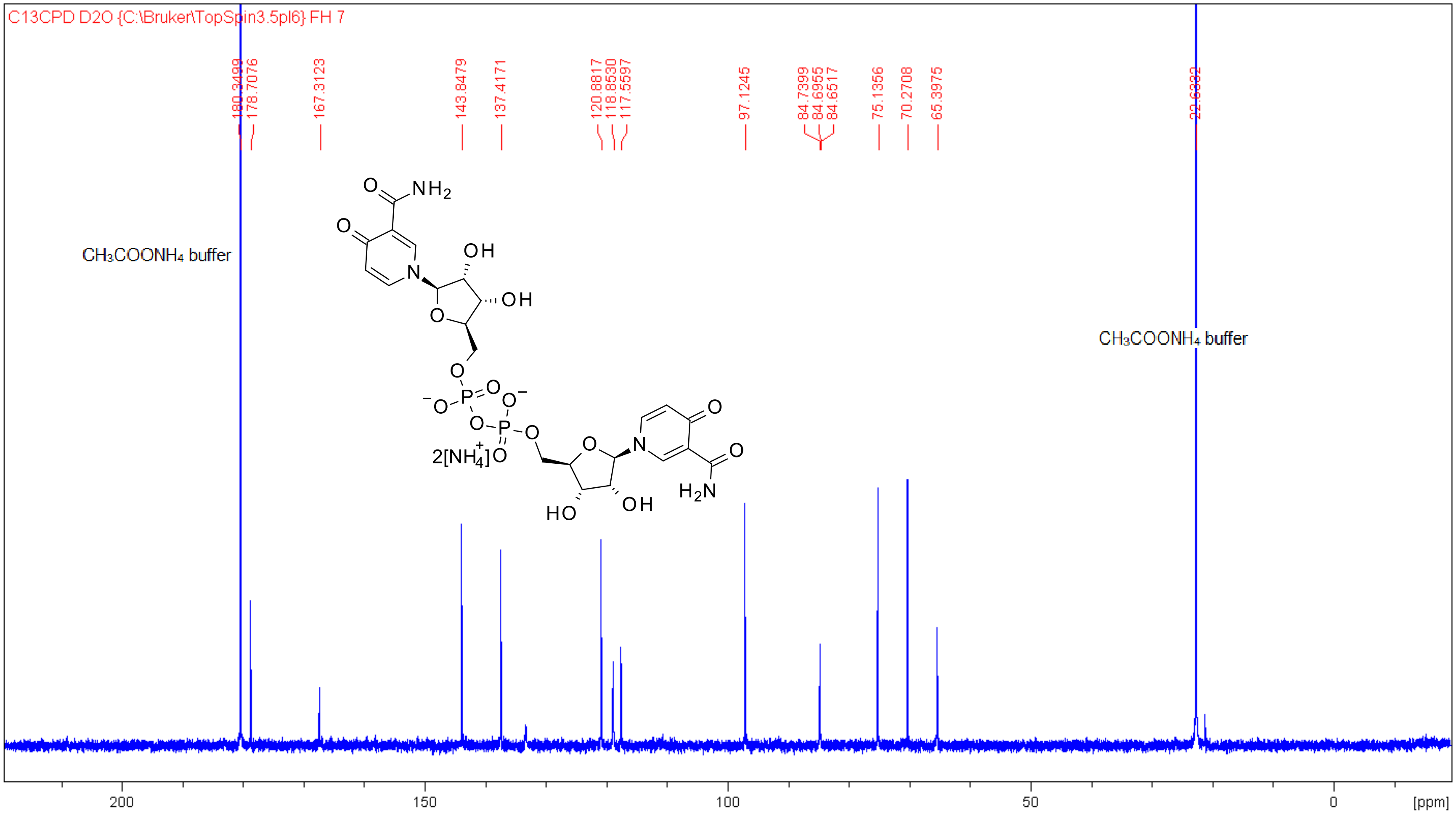

Compound 24. 100 MHz ^{13}C NMR spectrum in D_2O

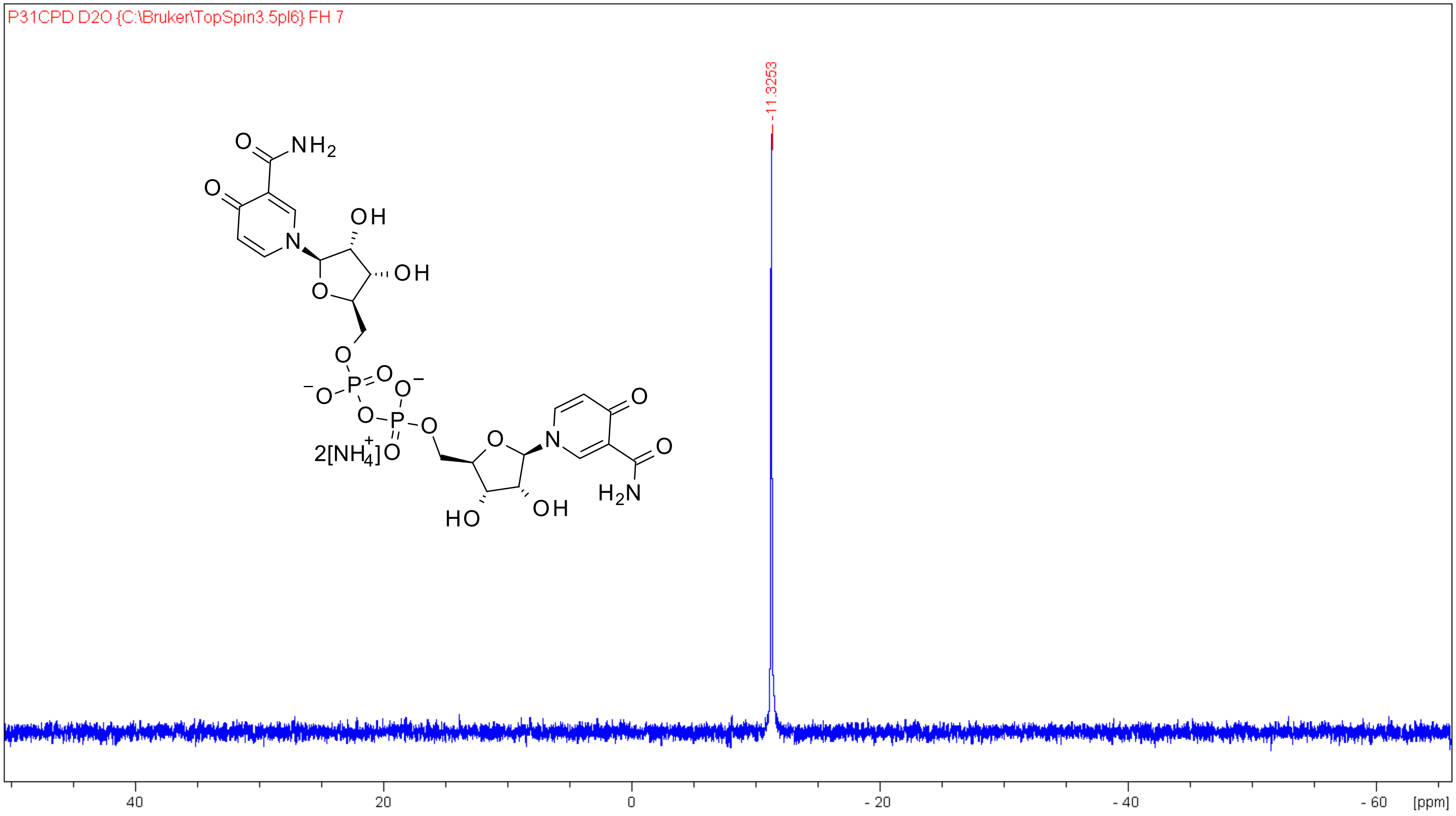

Compound 24. 162 MHz ^{31}P NMR spectrum in D_2O

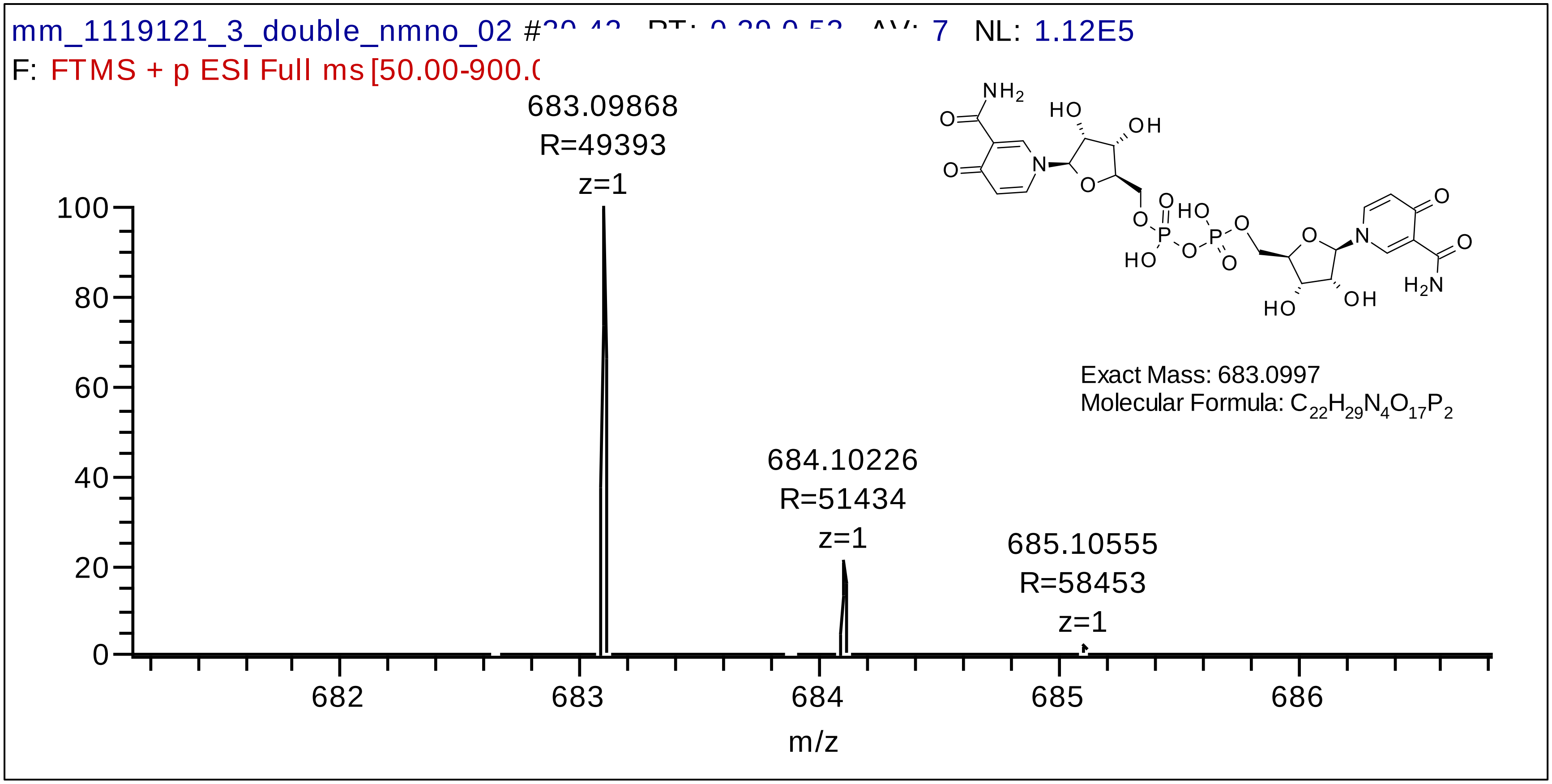

Compound 24. HRMS Sprctra

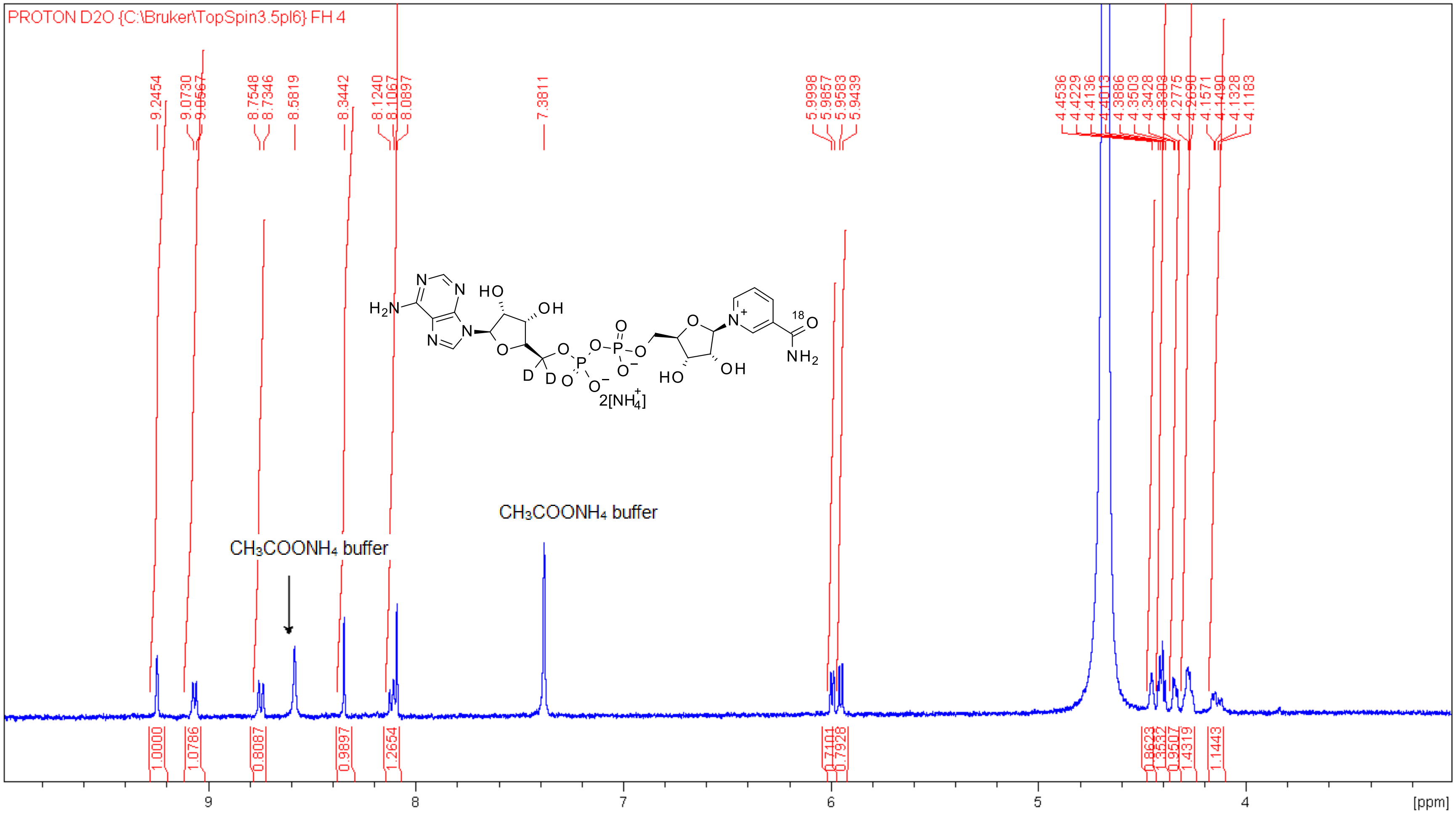

Compound 25. 400 MHz ^{1}H NMR spectrum in D_2O

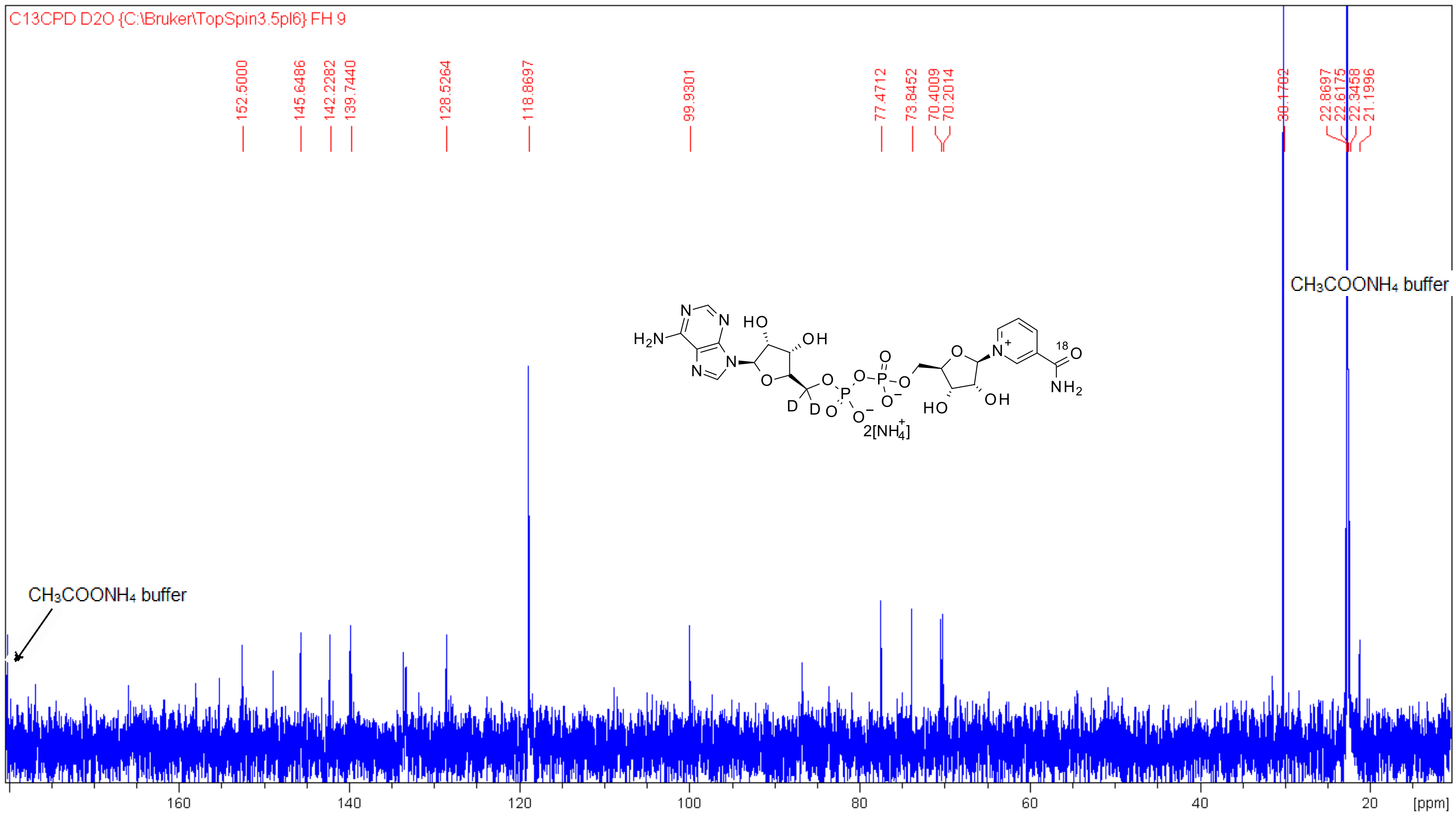

Compound 25. 100 MHz ^{13}C NMR spectrum in D_2O

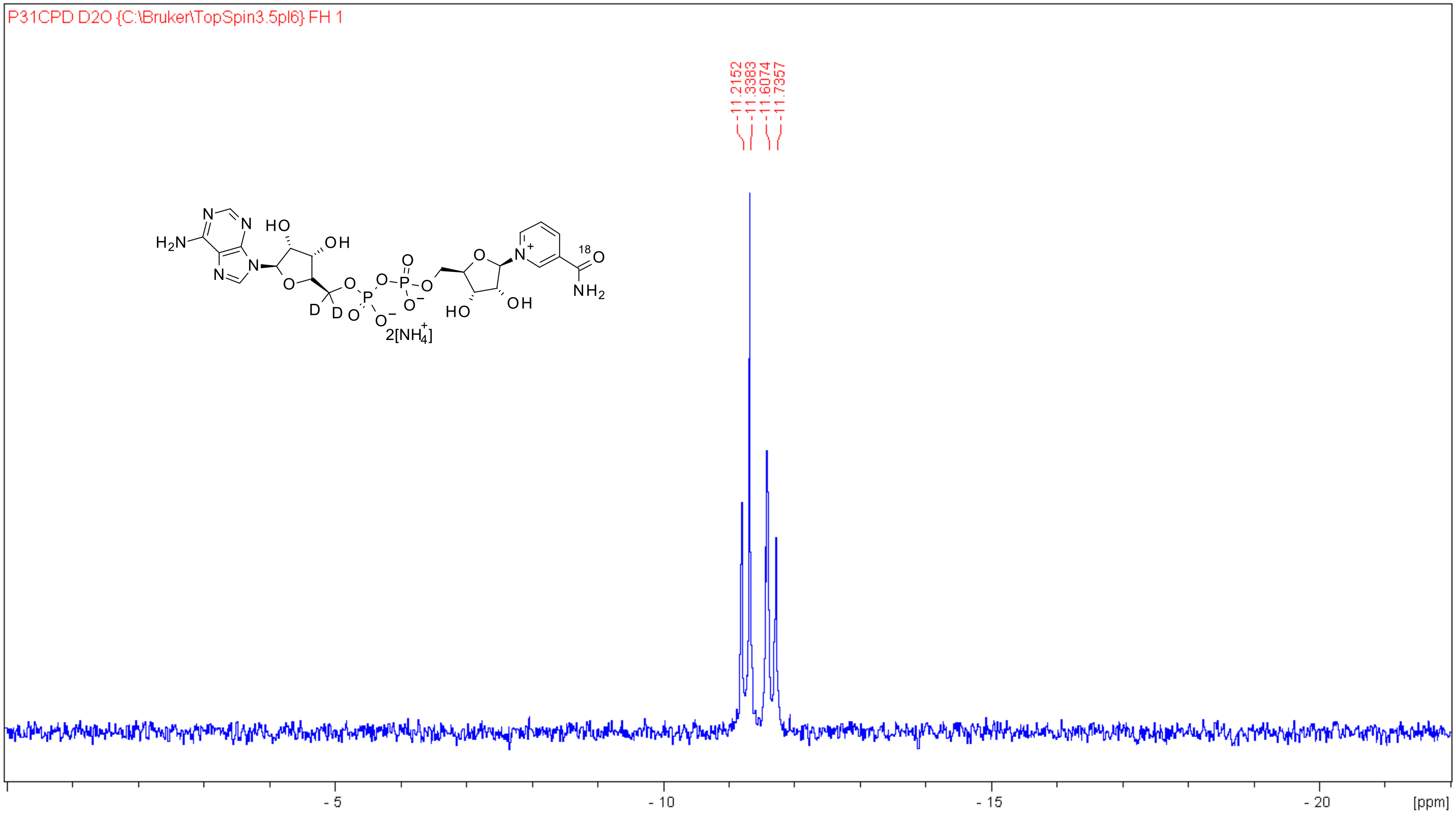

Compound 25. 162 MHz ^{31}P NMR spectrum in D_2O

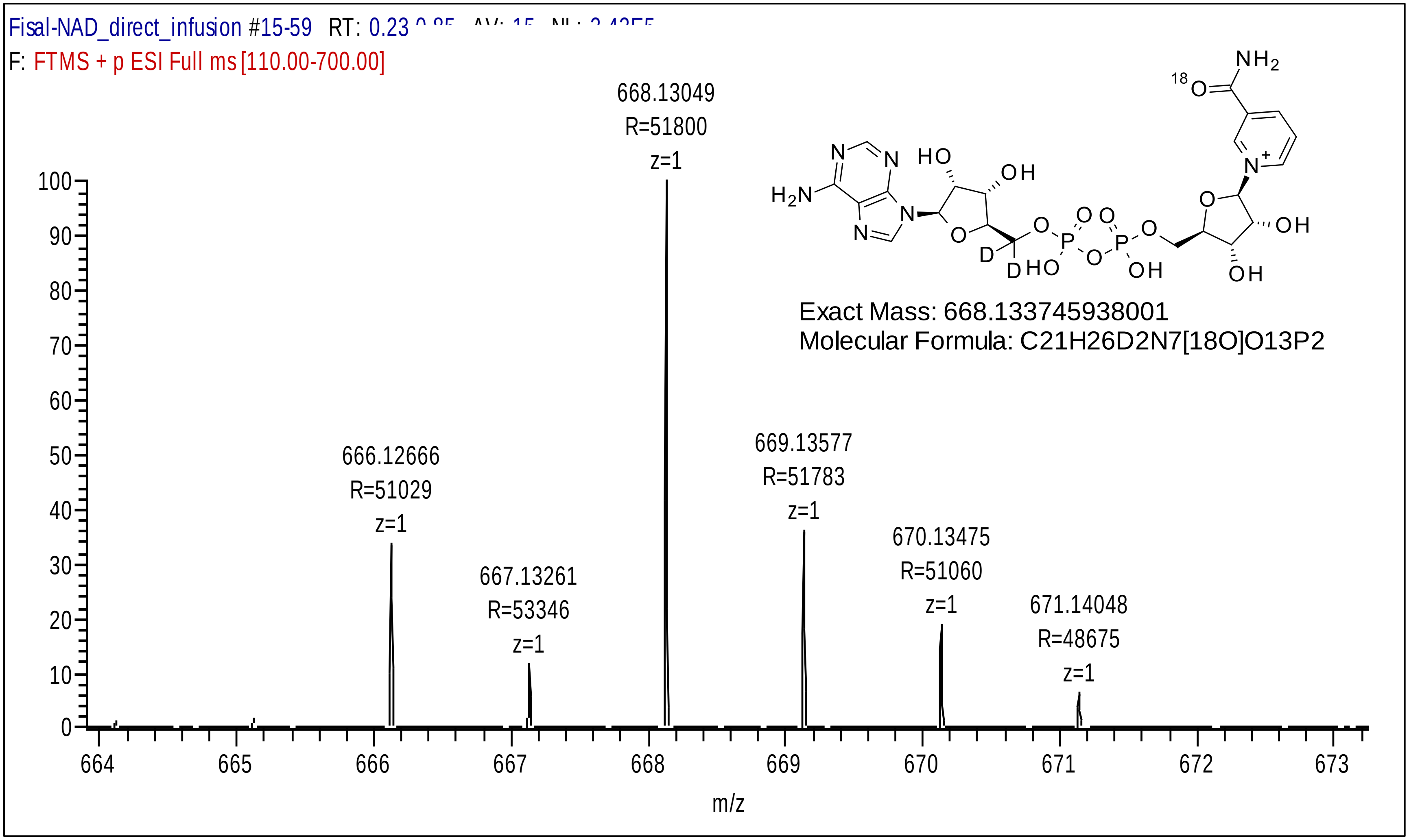

Compound 25. HRMS Spectra

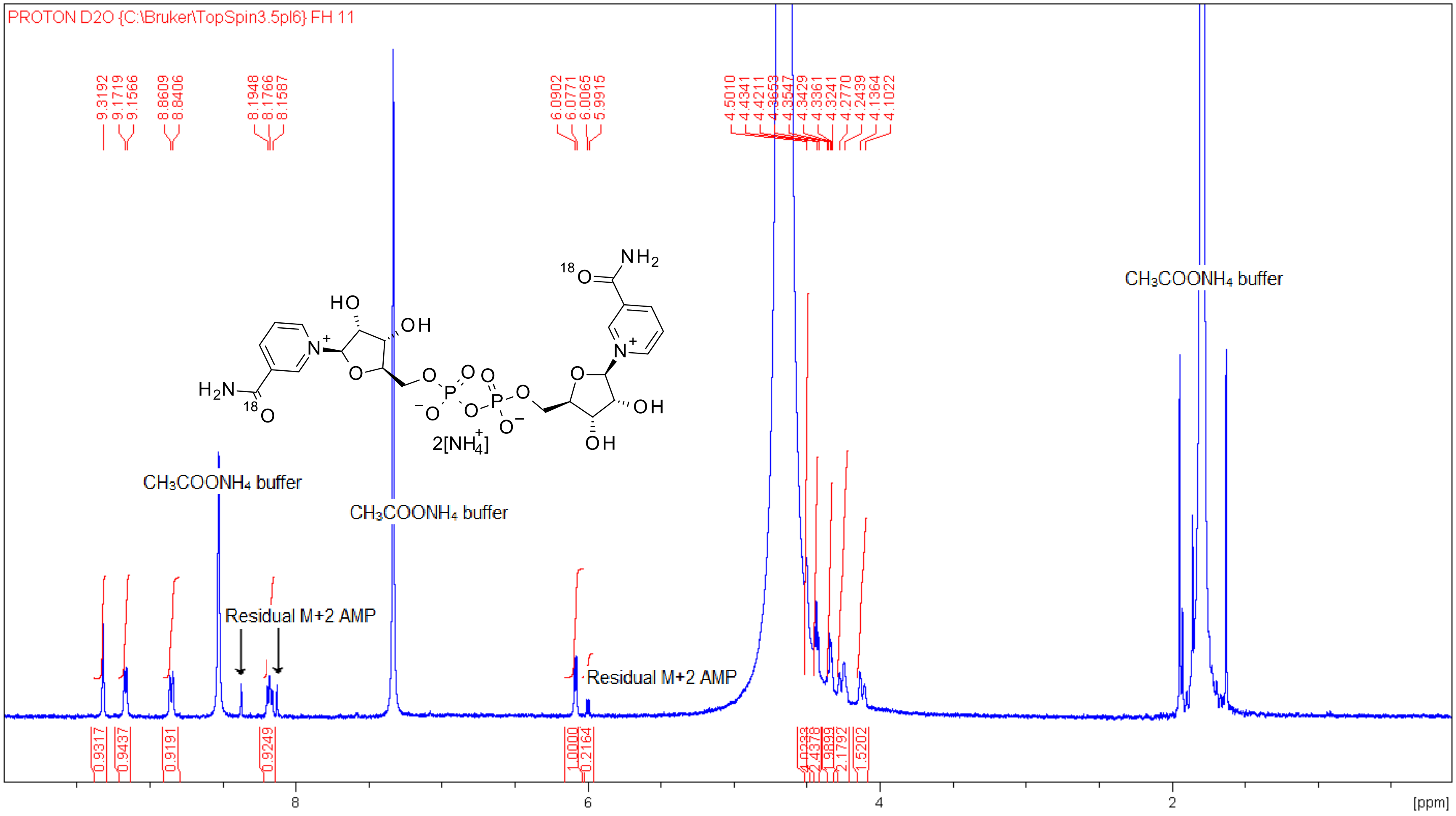

Compound 26. 400 MHz ^{1}H NMR spectrum in D_2O

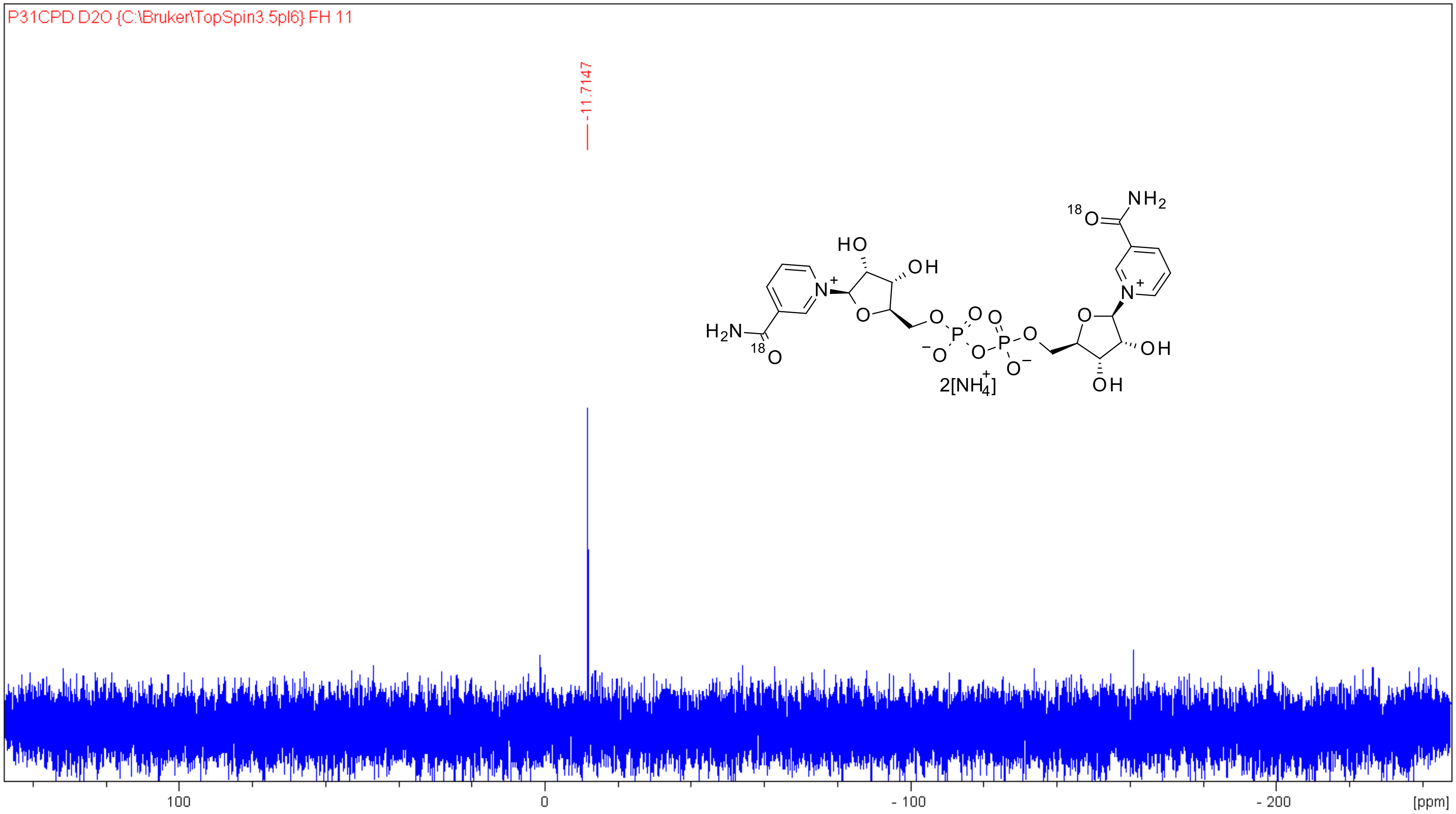

Compound 26. 162 MHz ^{31}P NMR spectrum in D_2O

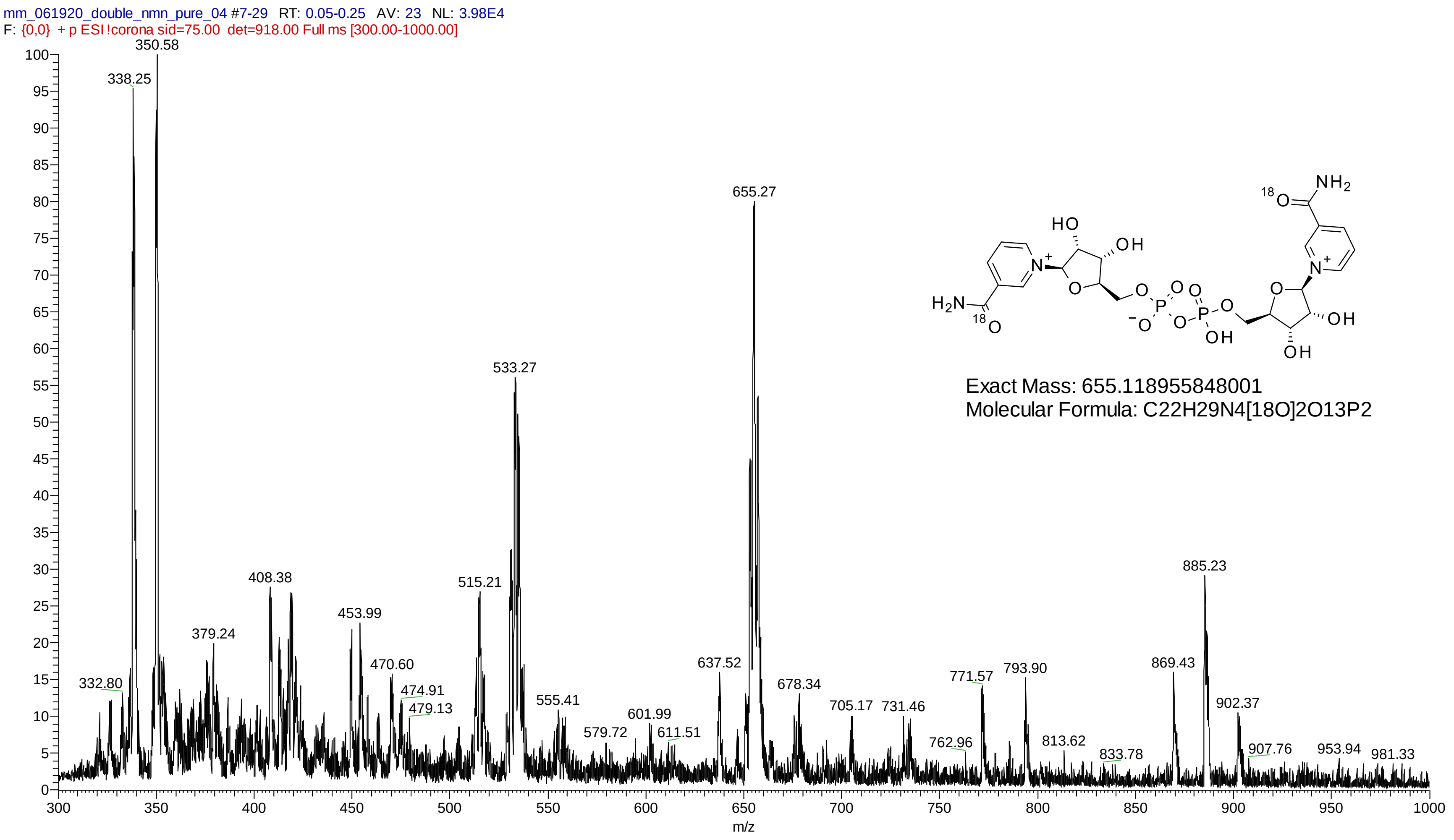

Compound 26. ESI-MS spectra

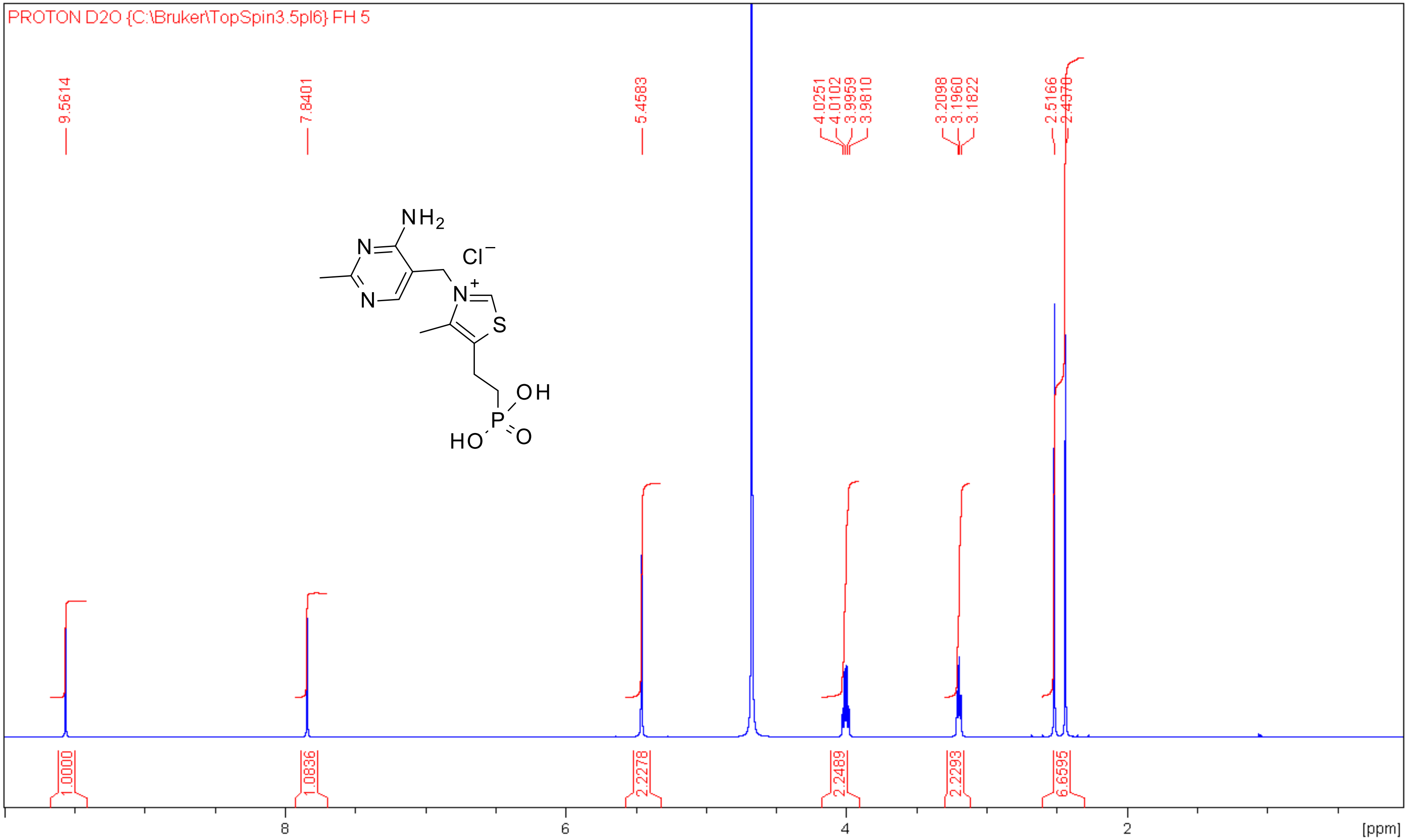

Compound 28. 400 MHz ^{1}H NMR spectrum in D_2O

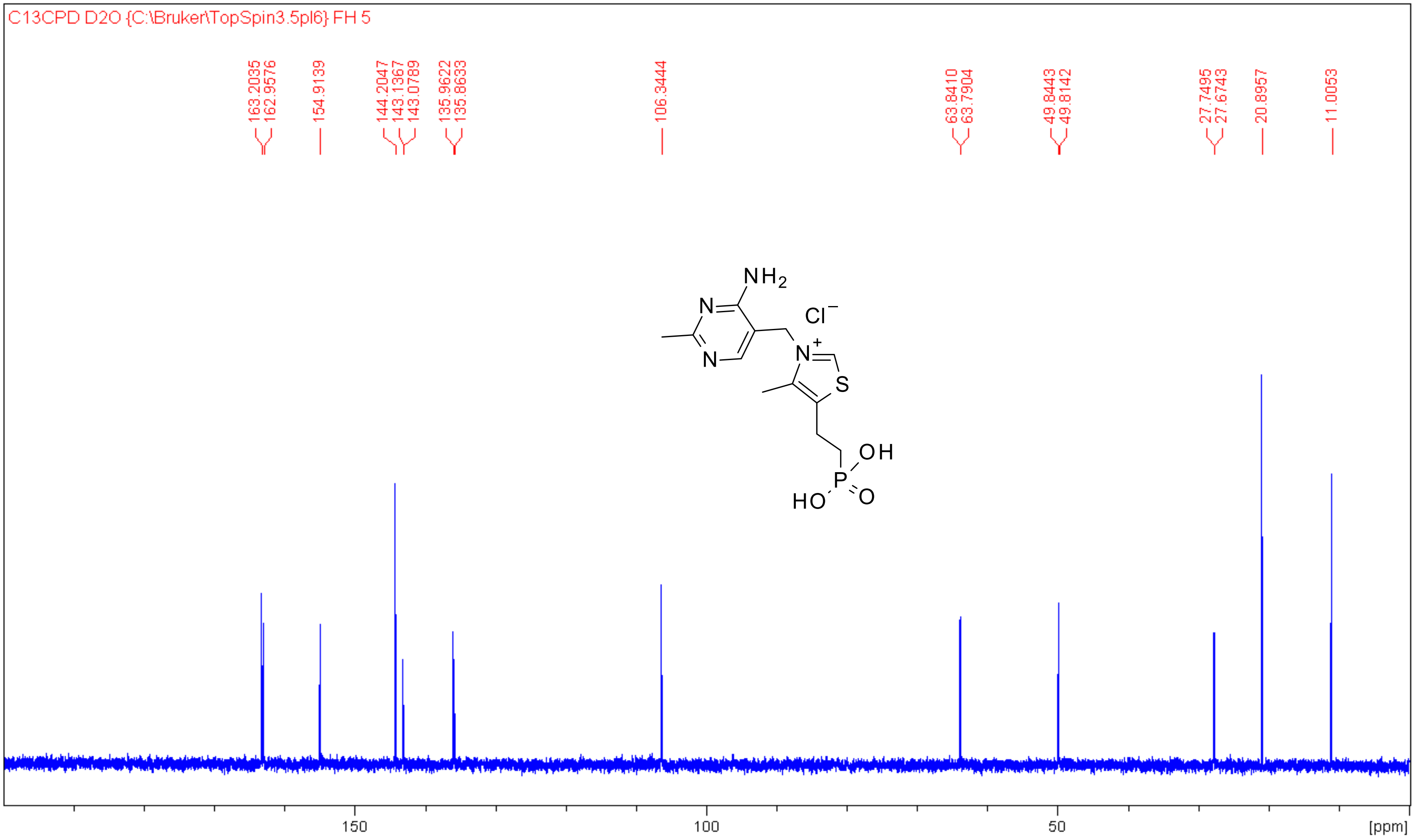

Compound 28. 100 MHz ^{13}C NMR spectrum in D_2O

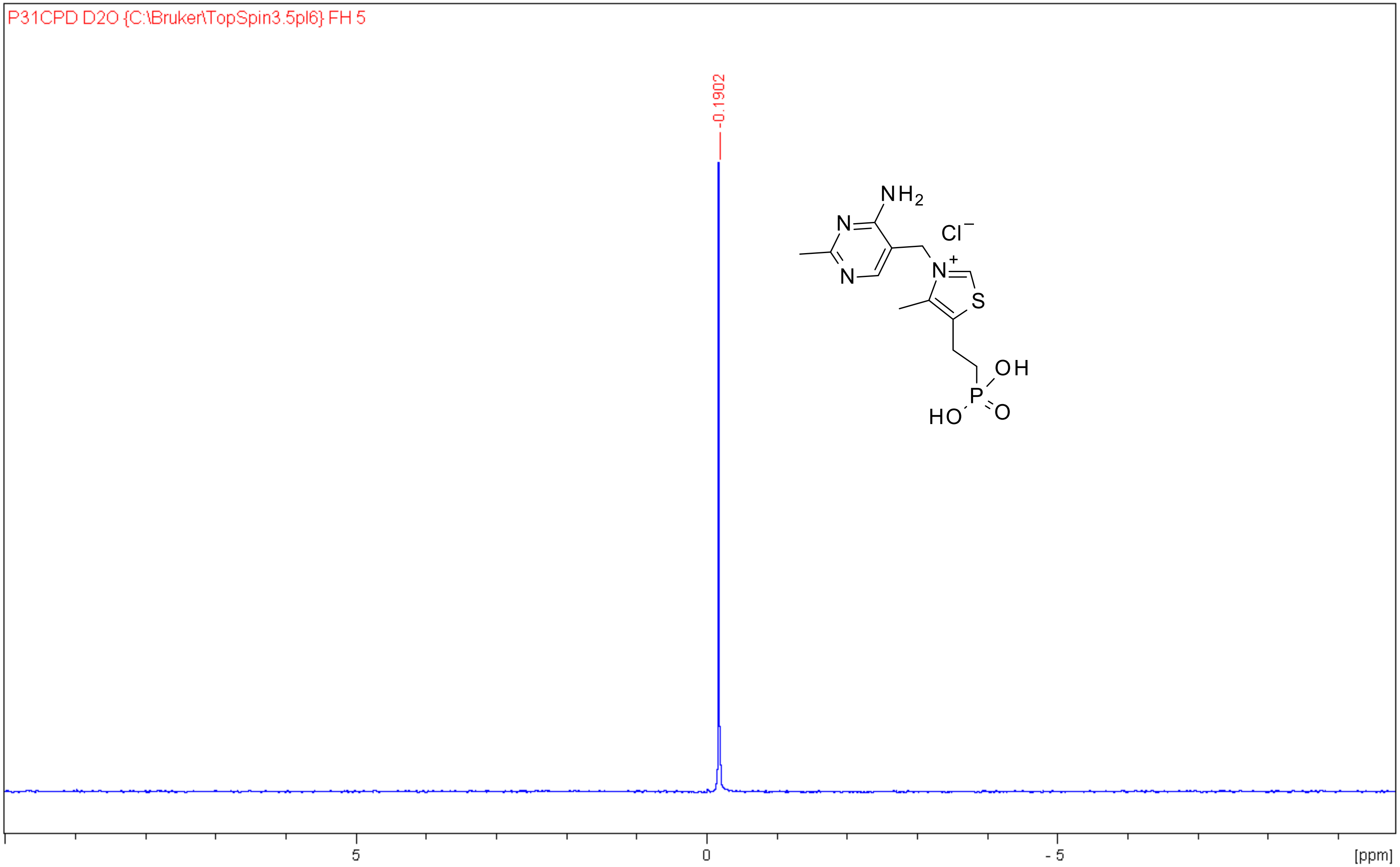

Compound 28. 162 MHz ^{31}P NMR spectrum in D_2O

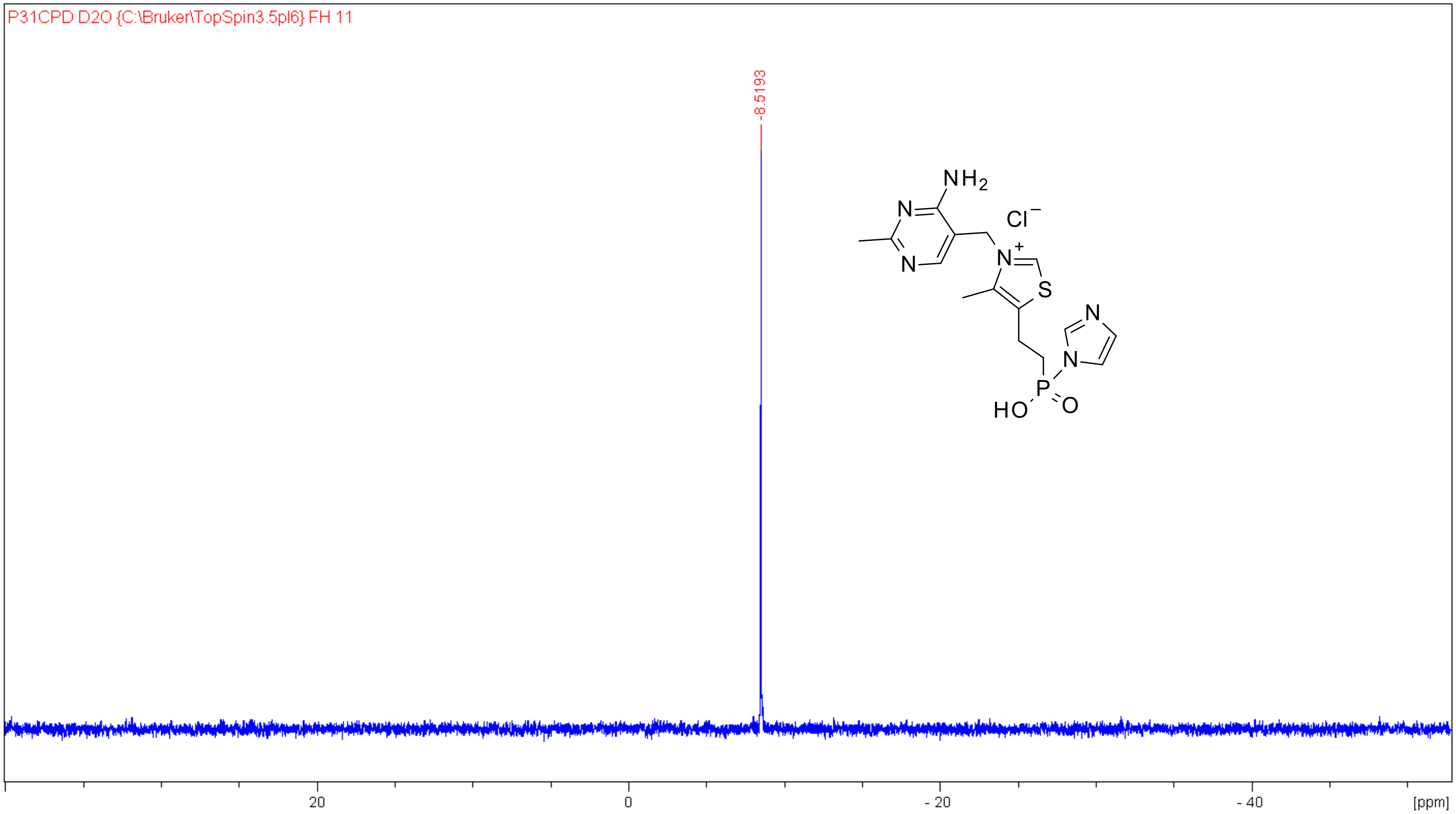

Compound 29. 162 MHz ^{31}P NMR spectrum in D_2O

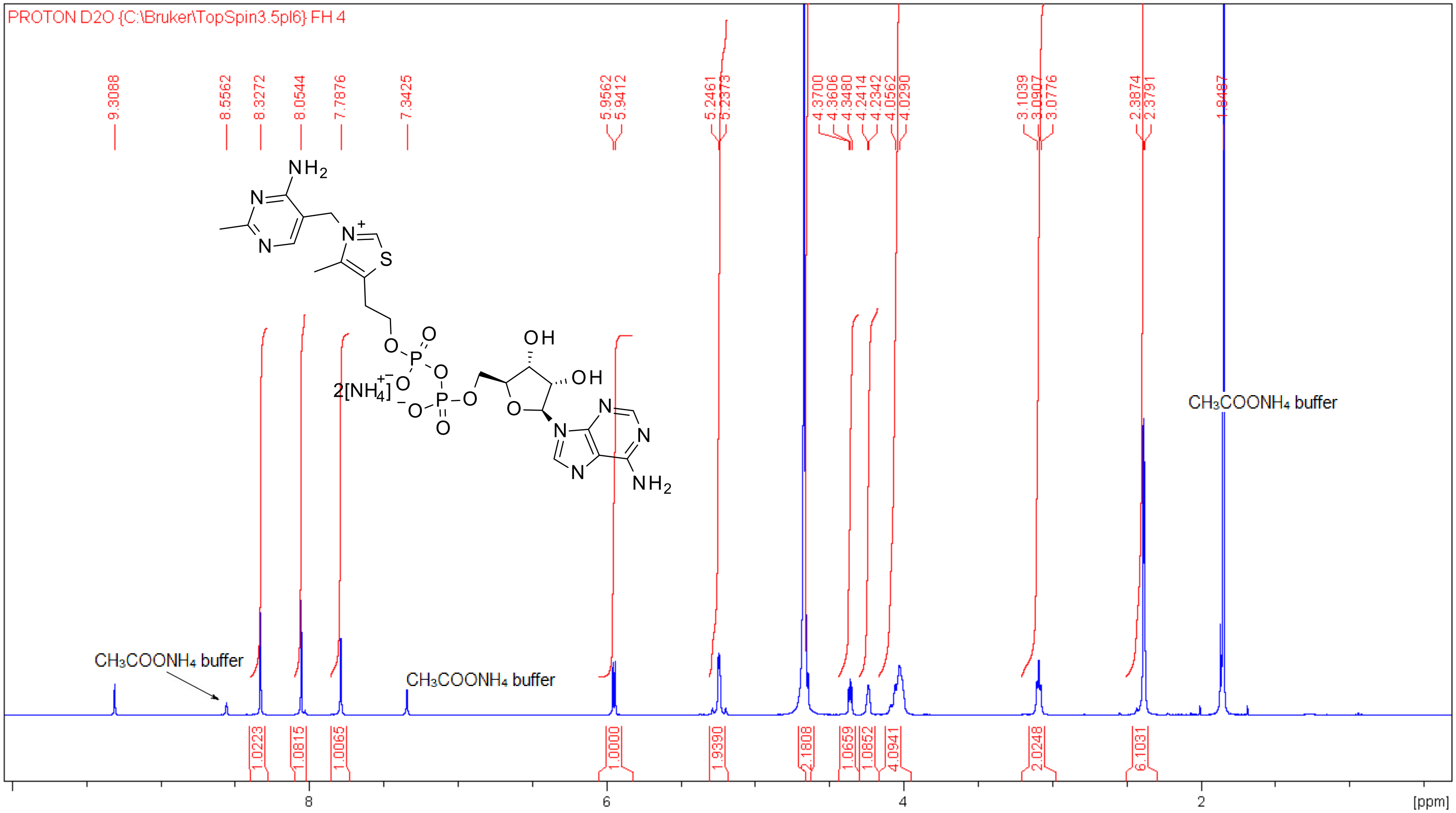

Compound 30a. 400 MHz ^{1}H NMR spectrum in D_2O

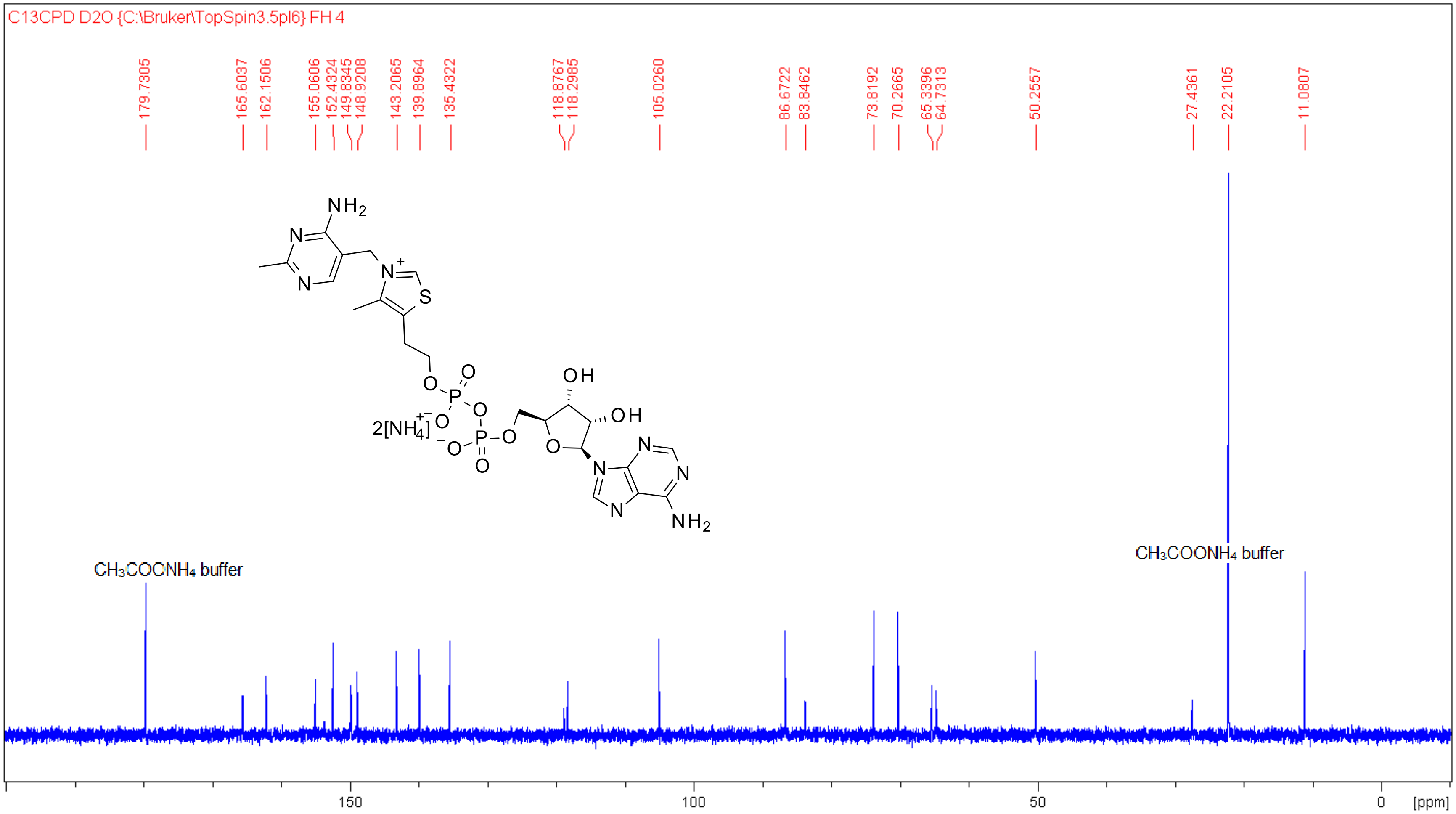
C13CPD D2O {C:\Bruker\TopSpin3.5pl6} FH 4
179.7305
165.6037
162.1506
155.0606
152.4324
149.8345
148.9208
143.2065
139.8964
135.4322
118.8767
118.2985
105.0260
86.6722
83.8462
73.8192
70.2665
65.3396
64.7313
50.2557
27.4361
22.2105
11.0807
CH3COONH4 buffer
CH3COONH4 buffer
2[NH4]+
150
100
50
0
[ppm]

Compound 30a. 100 MHz ^{13}C NMR spectrum in D_2O

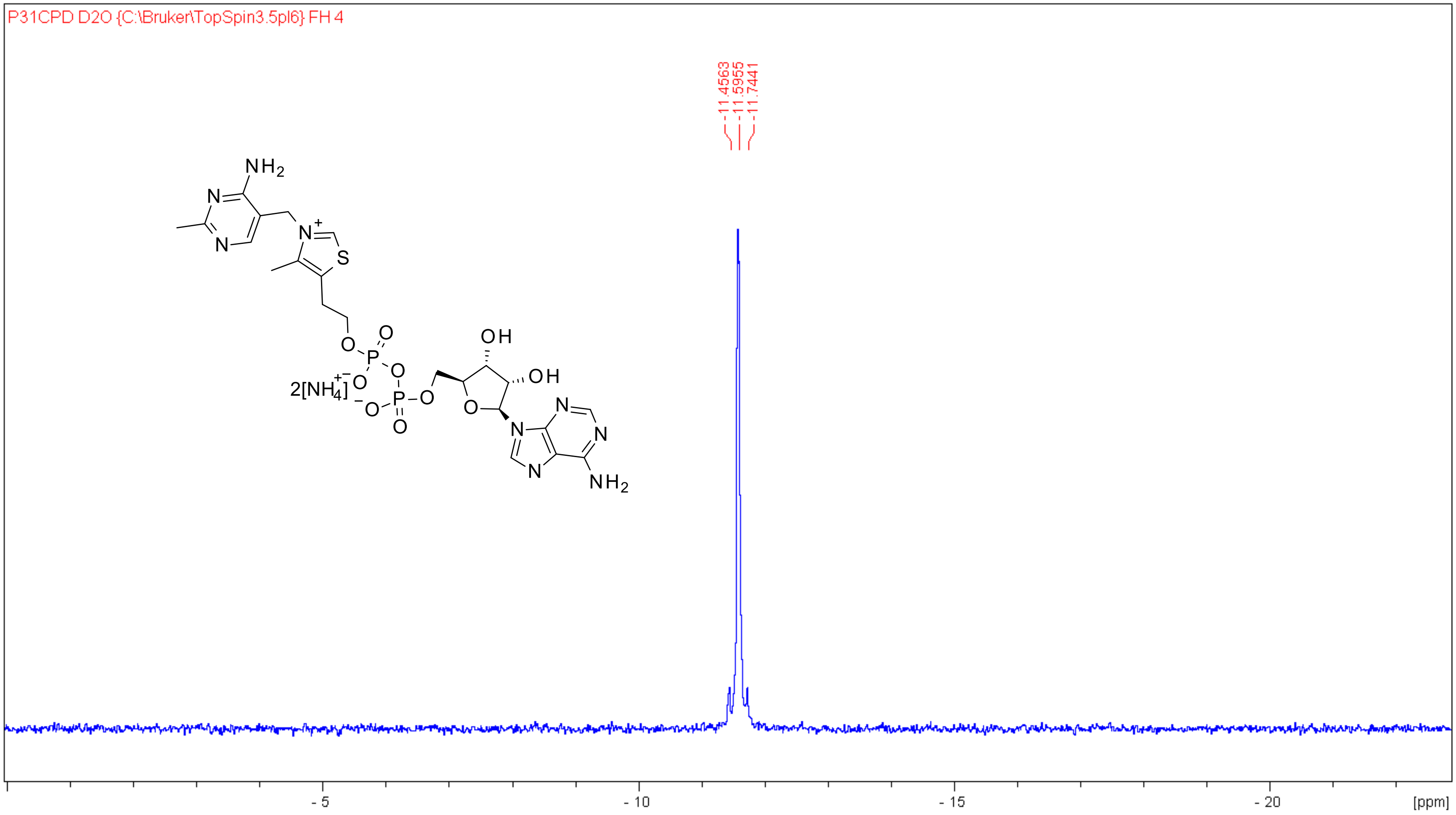

Compound 30a. 162 MHz ^{31}P NMR spectrum in D_2O

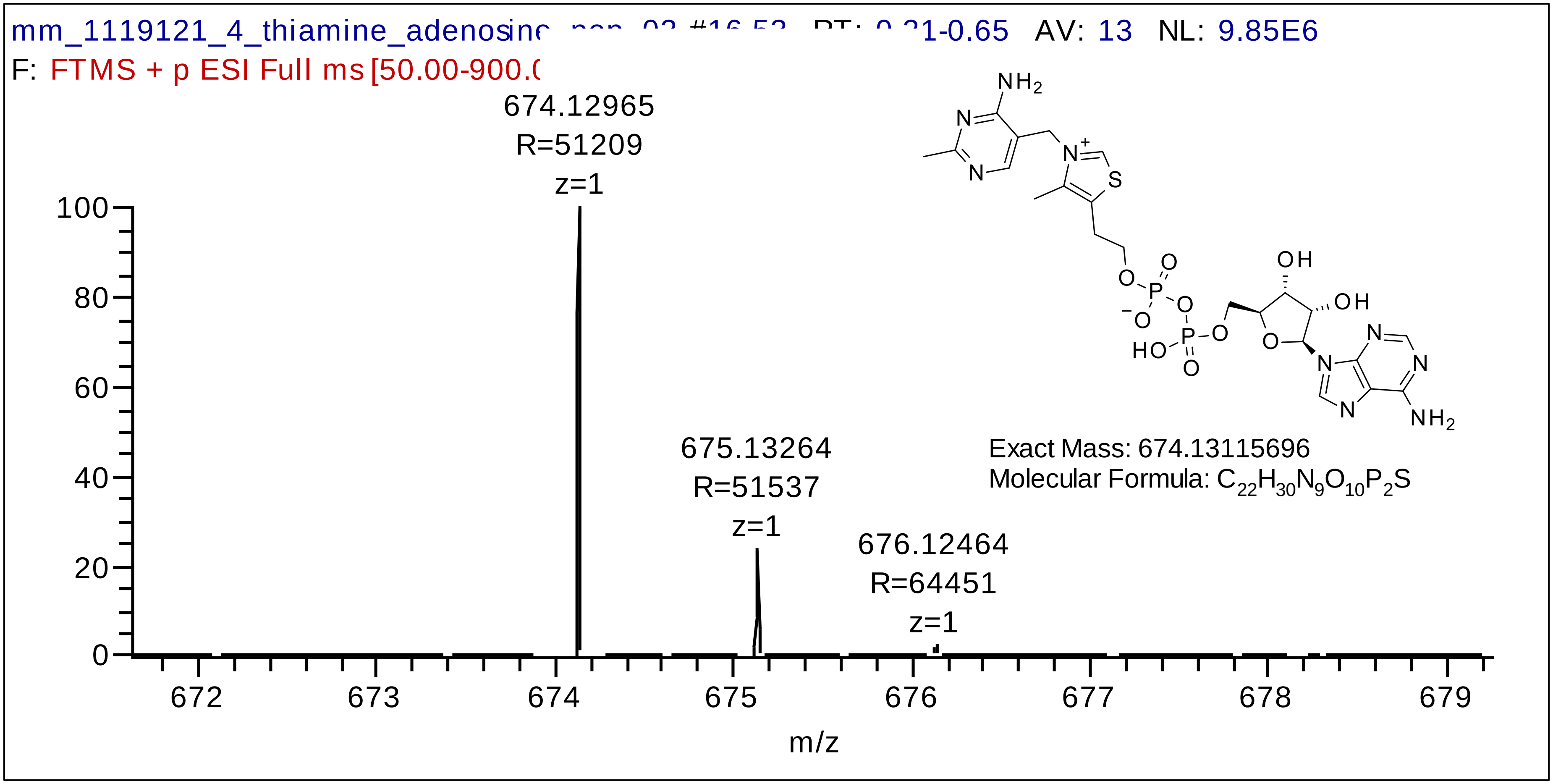

Compound 30a. HRMS spectra

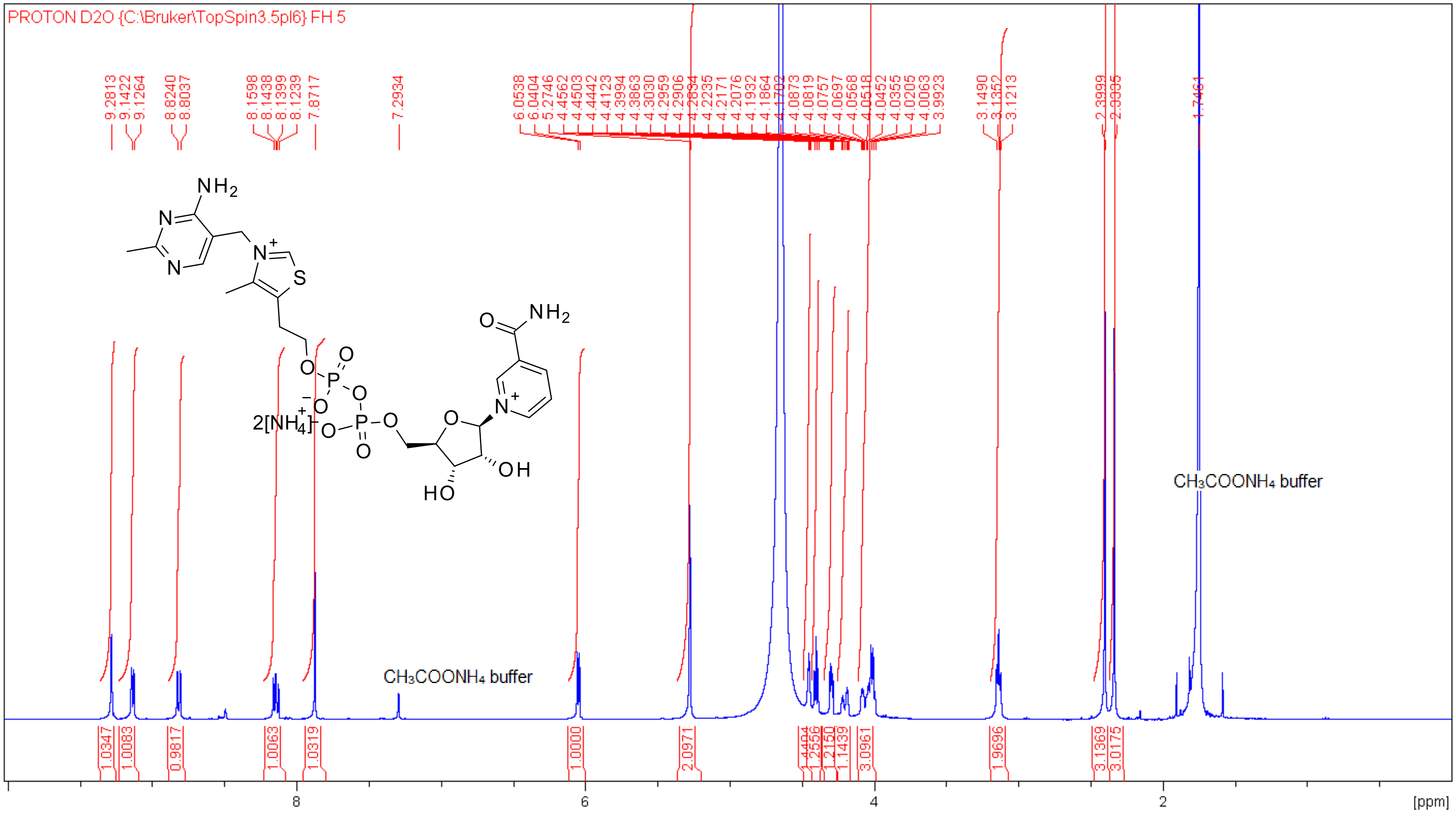

Compound 31. 400 MHz ^{1}H NMR spectrum in D_2O

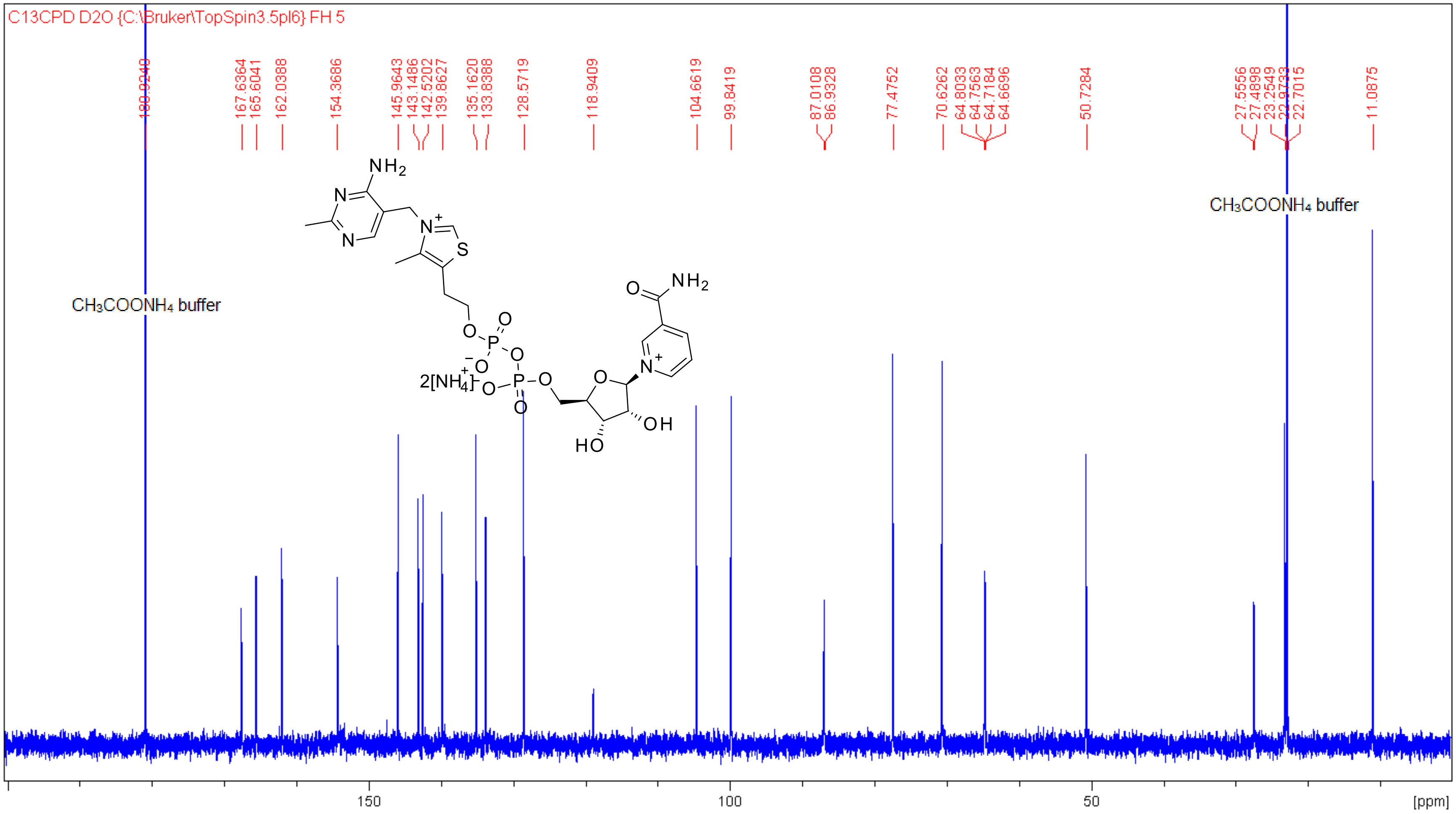

Compound 31. 100 MHz ^{13}C NMR spectrum in D_2O

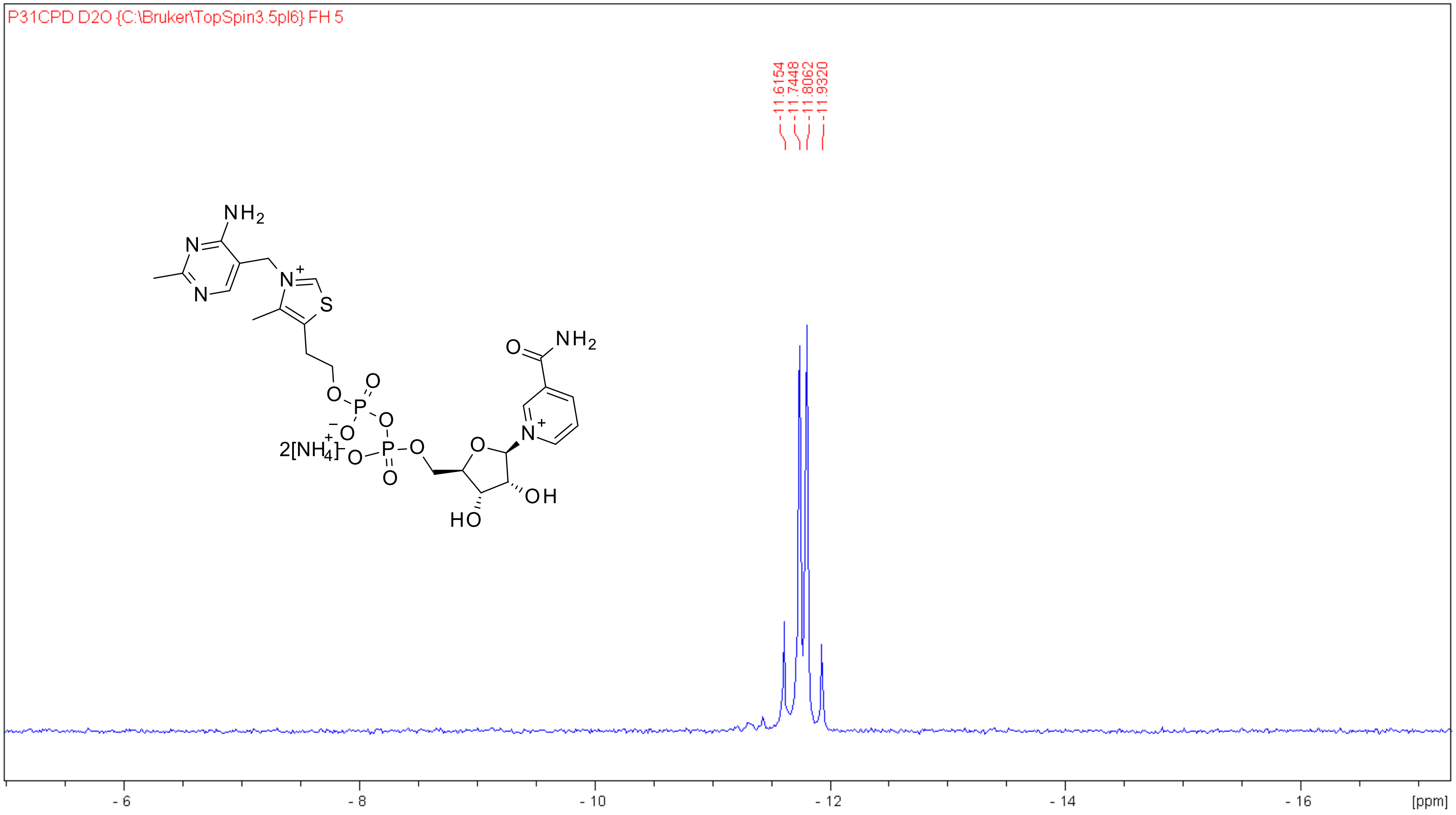

Compound 31. 162 MHz ^{31}P NMR spectrum in D_2O

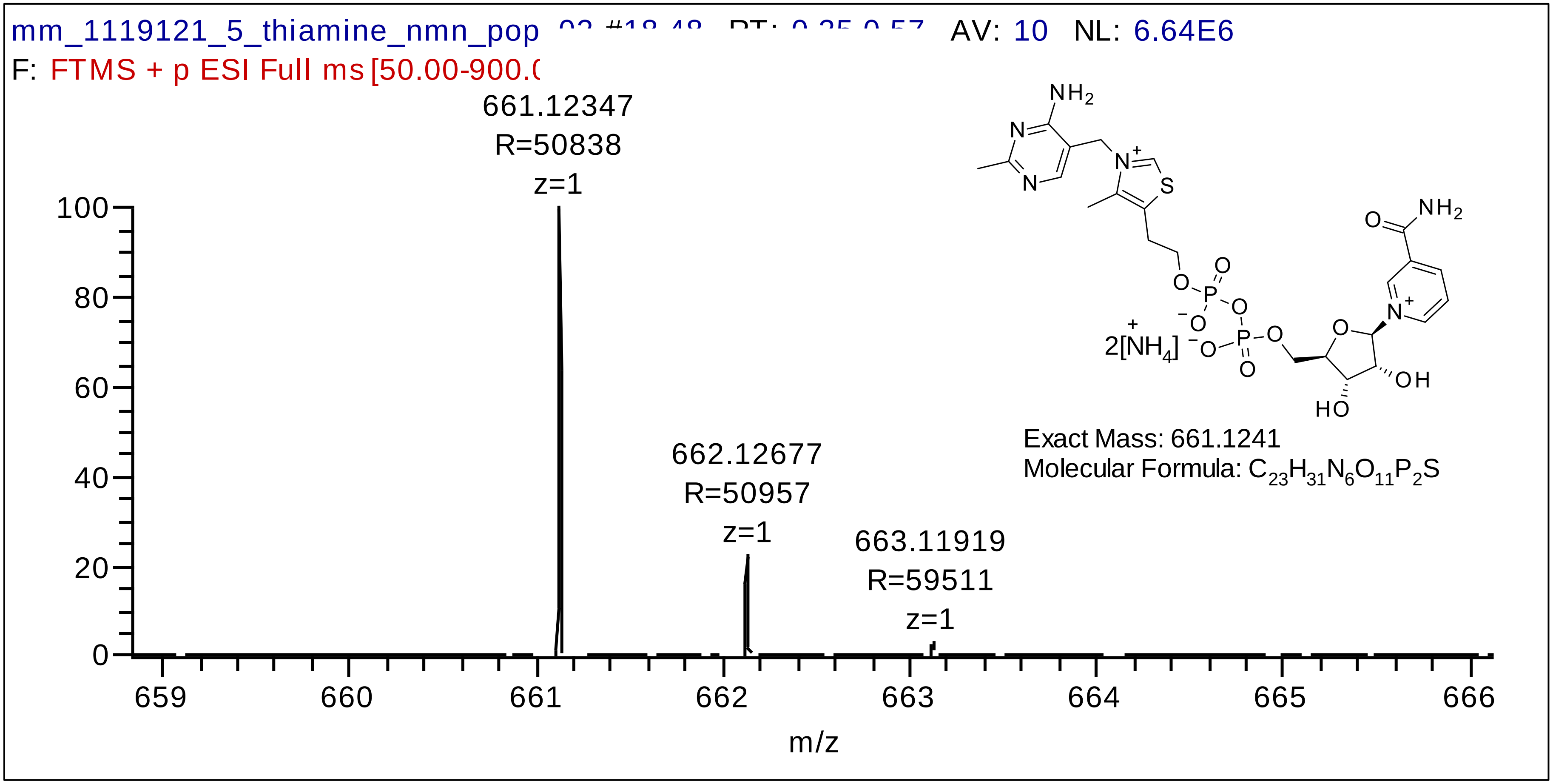

Compound 31. HRMS spectra

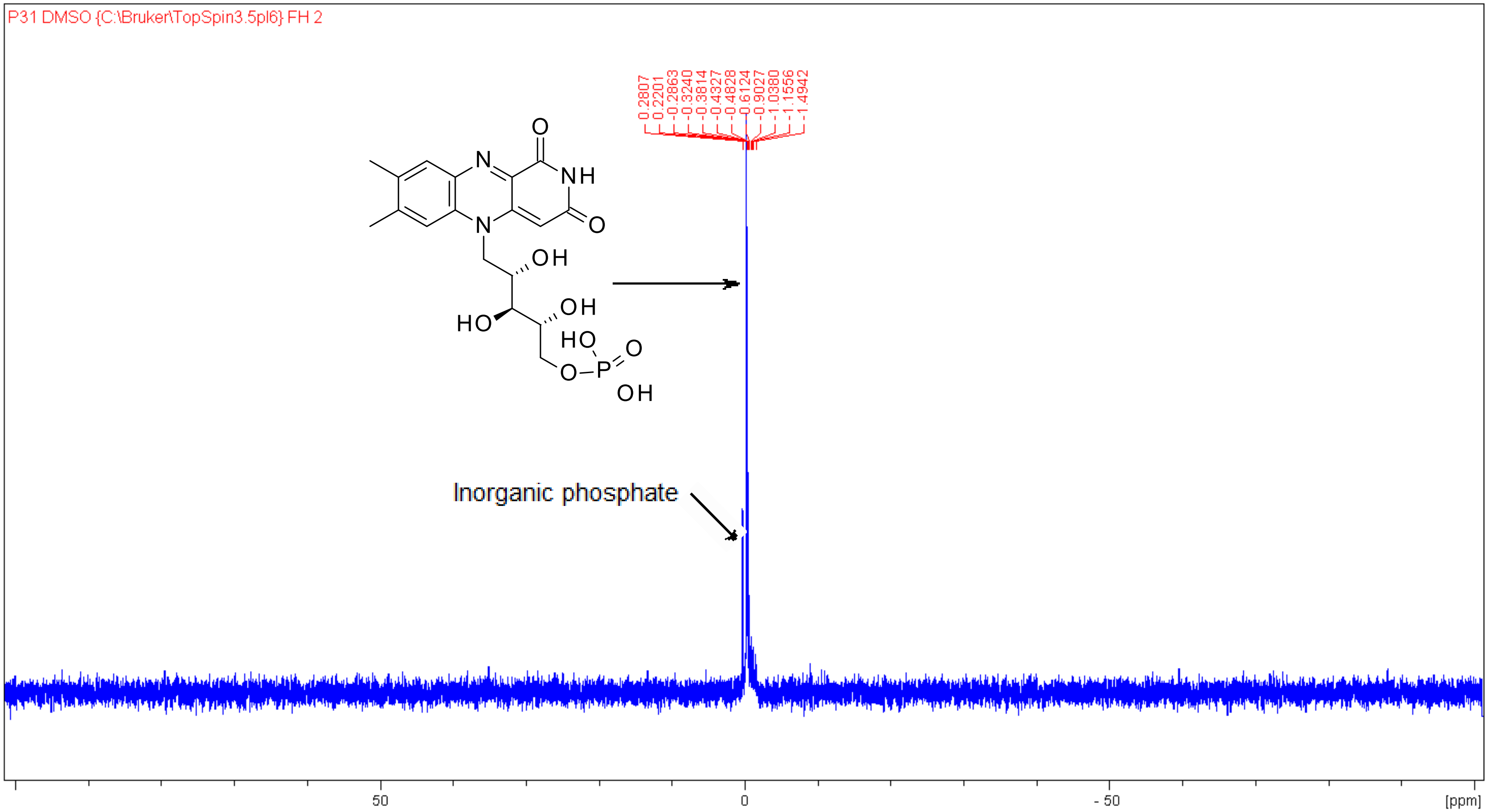

Compound 32. 162 MHz ^{31}P NMR spectrum in D_2O

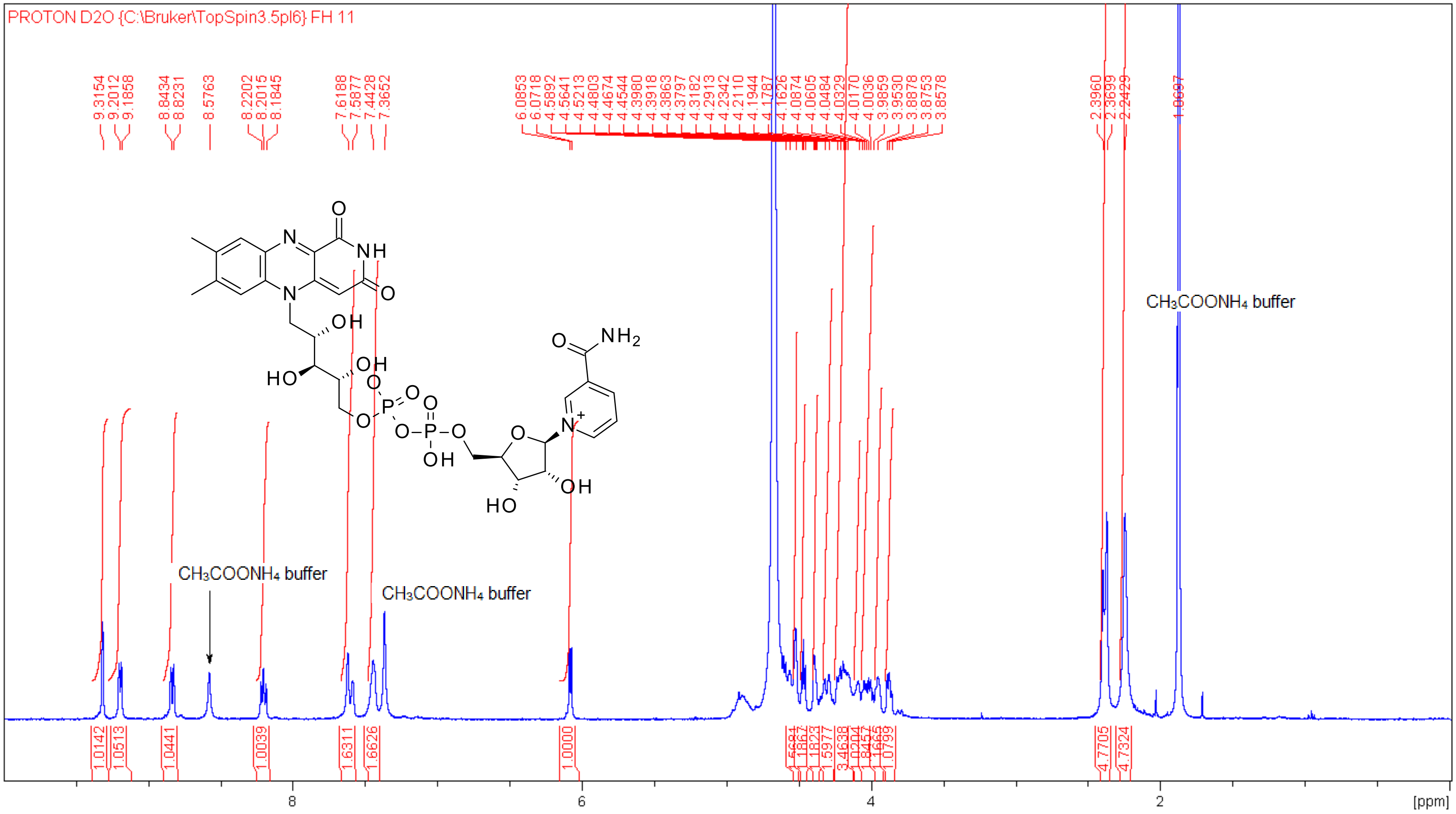

Compound33. 400 MHz ^{1}H NMR spectrum in D_2O

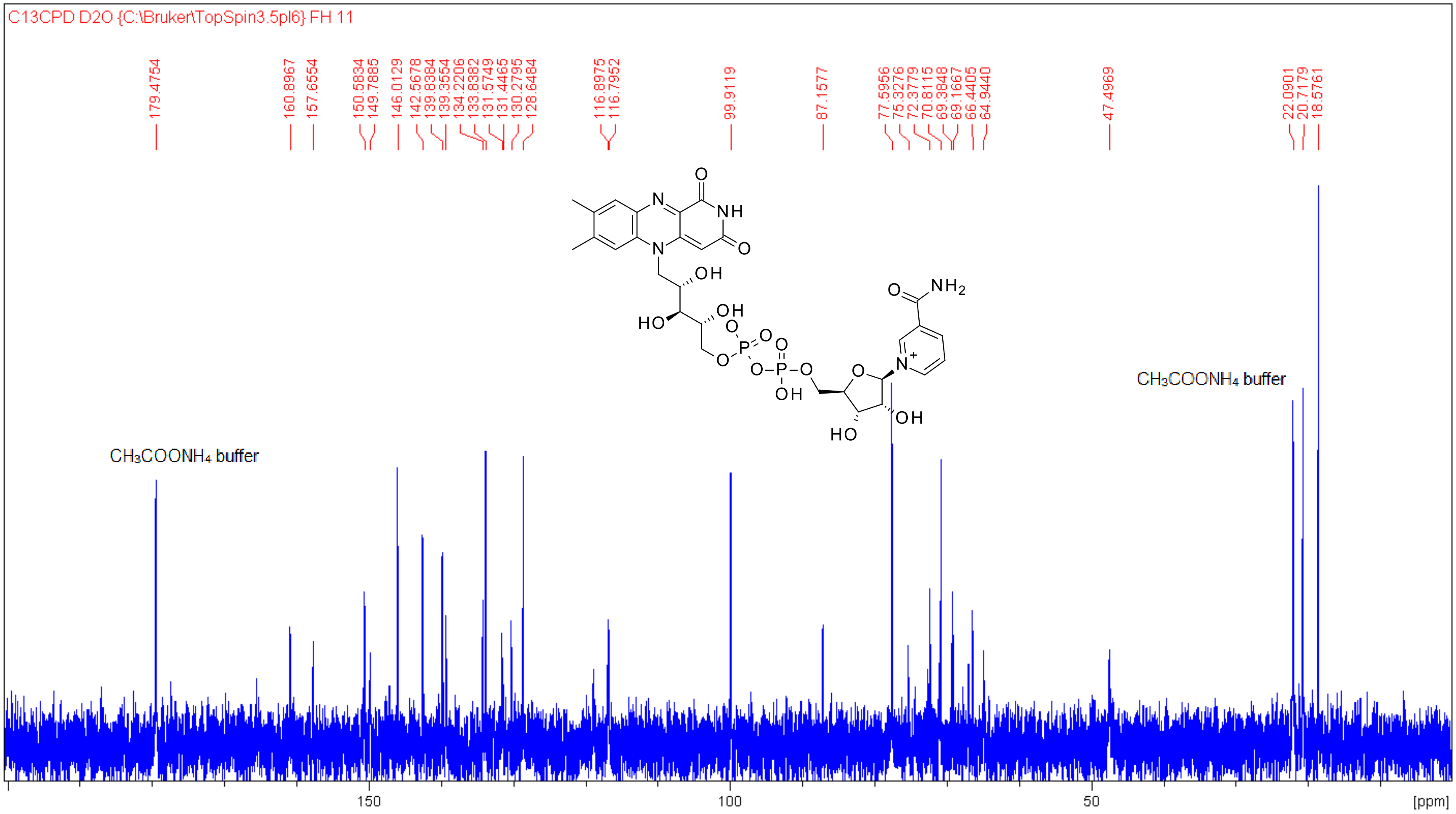

Compound 33. 100 MHz ^{13}C NMR spectrum in D_2O

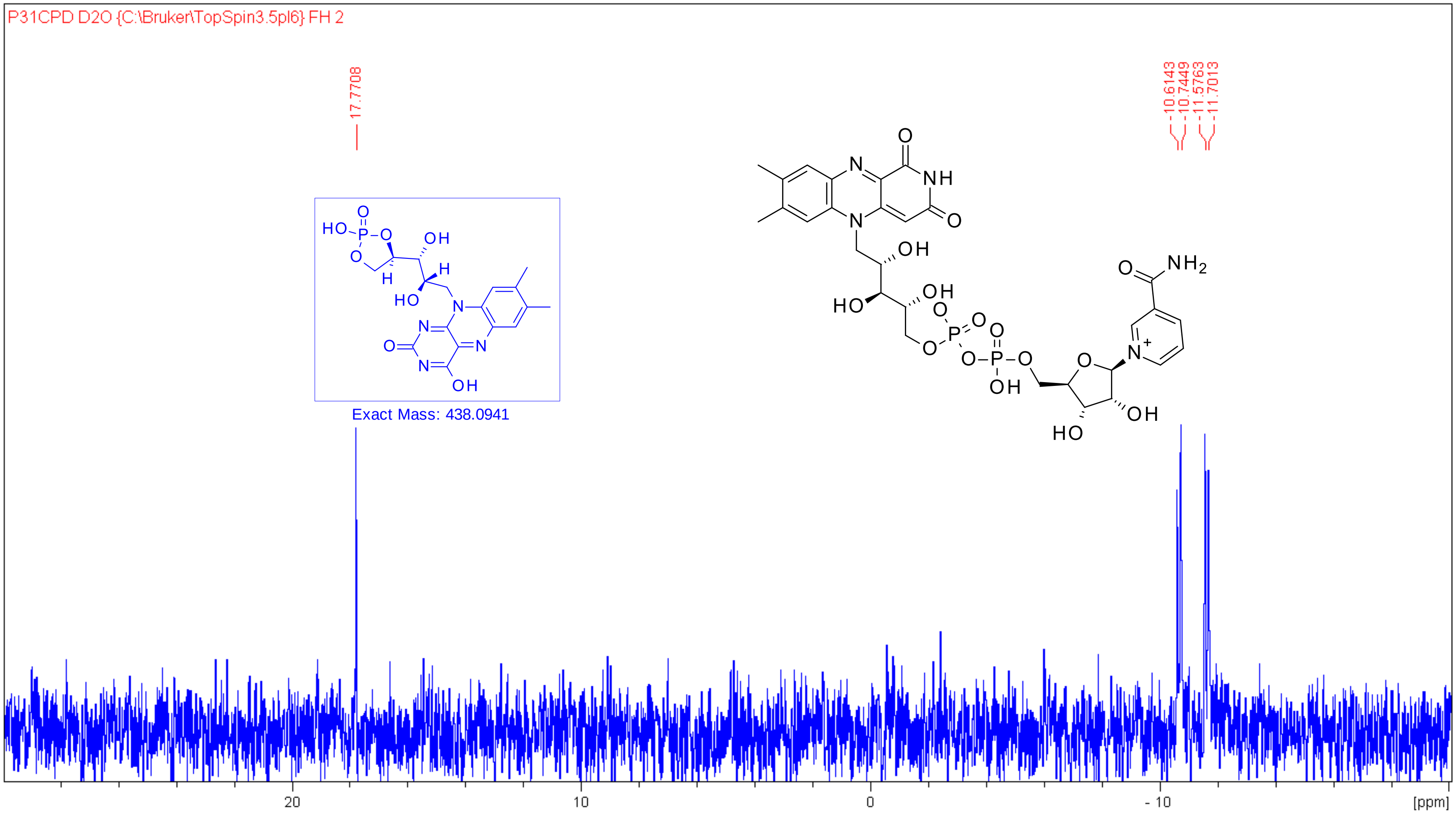

Compound 33. 162 MHz ^{31}P NMR spectrum in D_2O

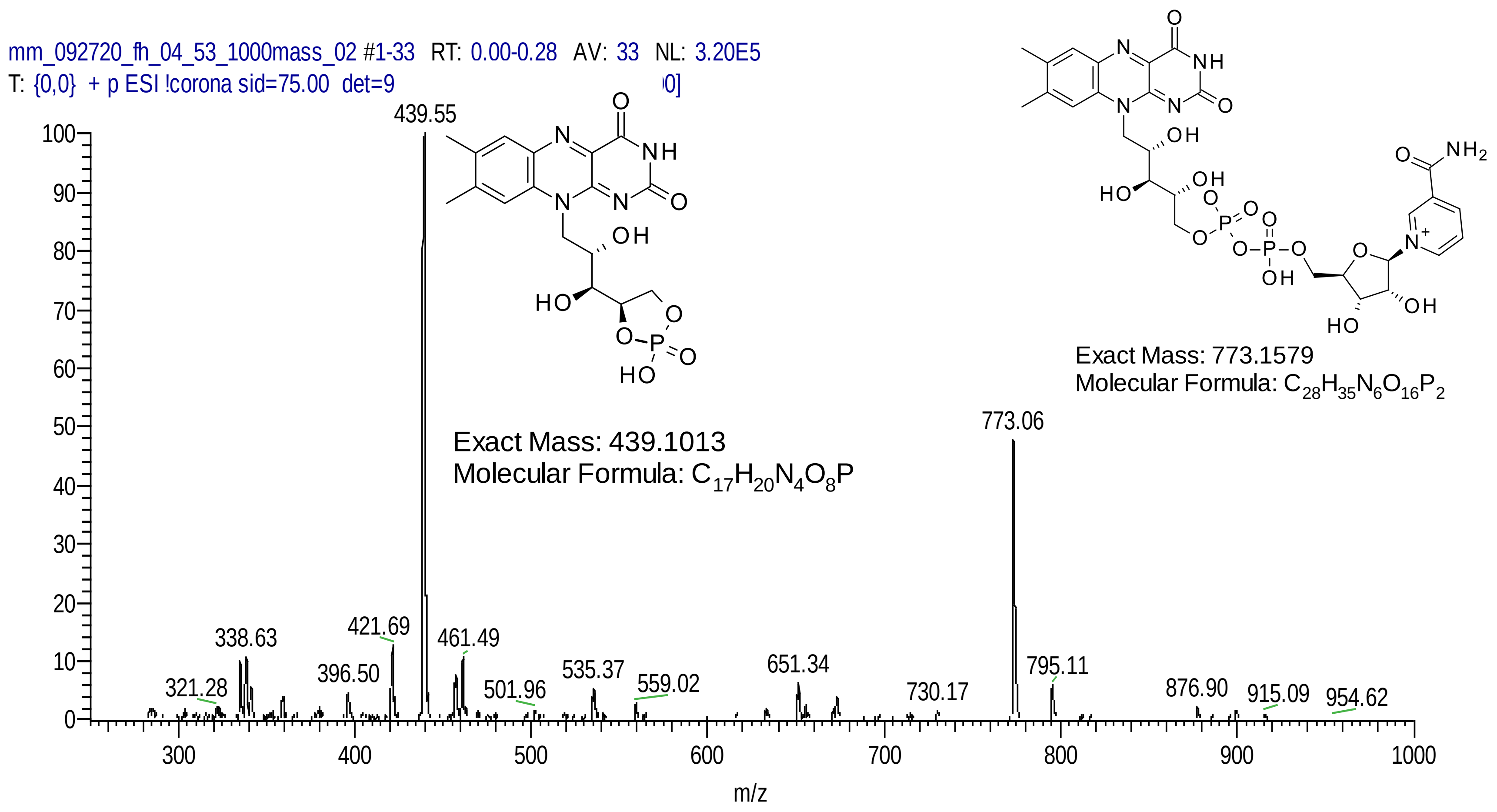

Compound 33&34. ESI-MS spectra

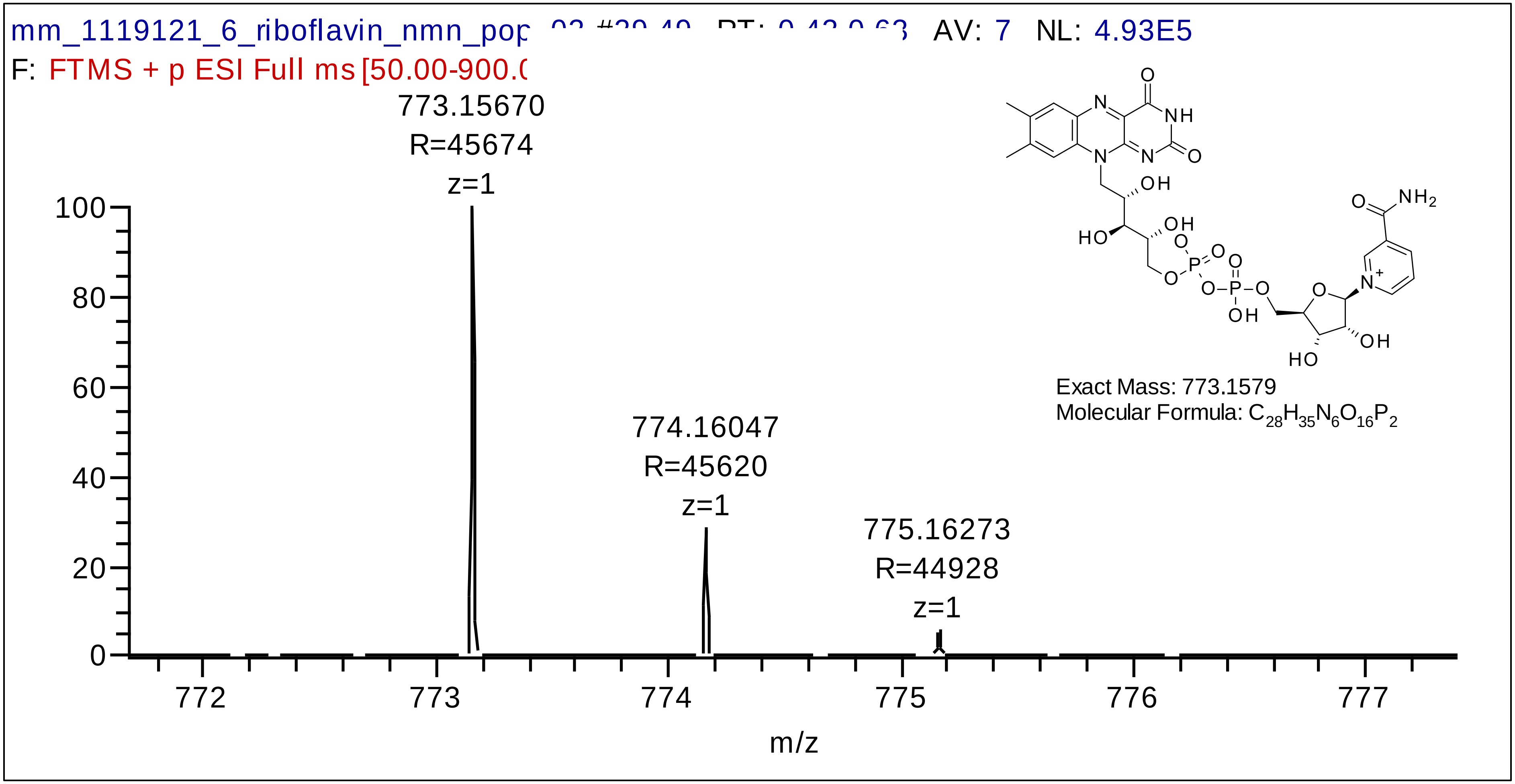

Compound 33. HRMS spectra

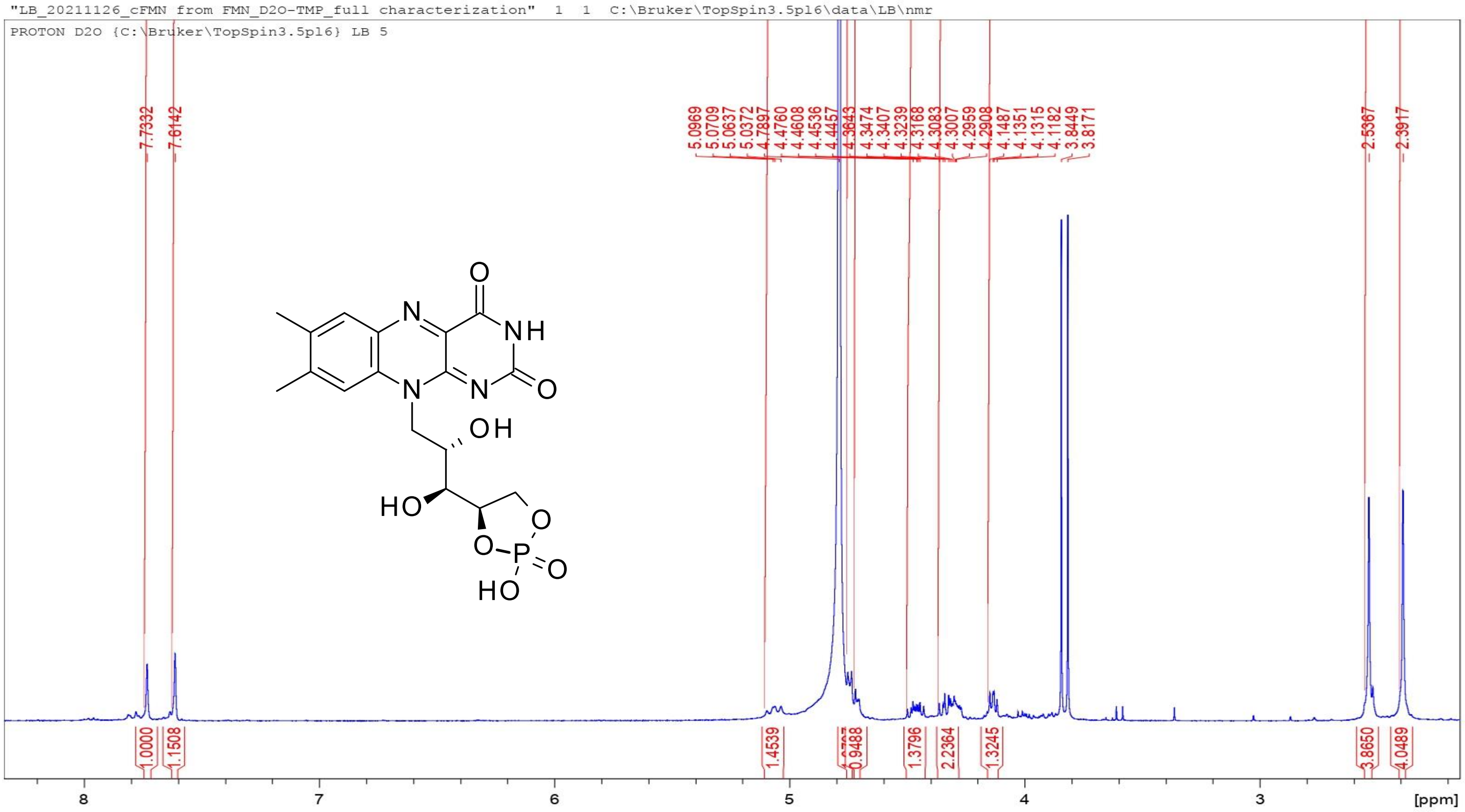

Compound 34. 400 MHz ^1H NMR spectrum in D_2O

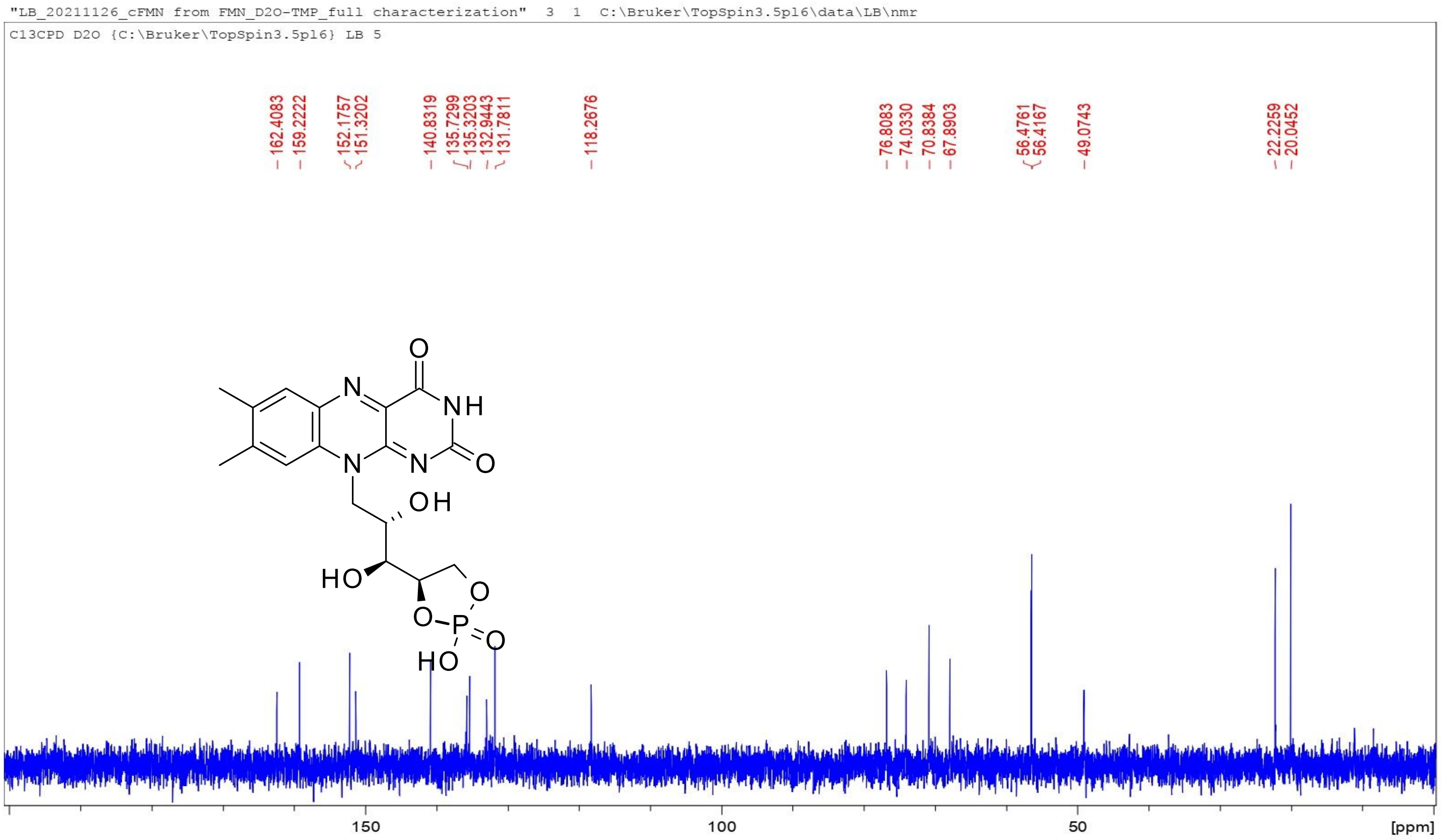

Compound 34. 100 MHz ^{13}C NMR spectrum in D_2O

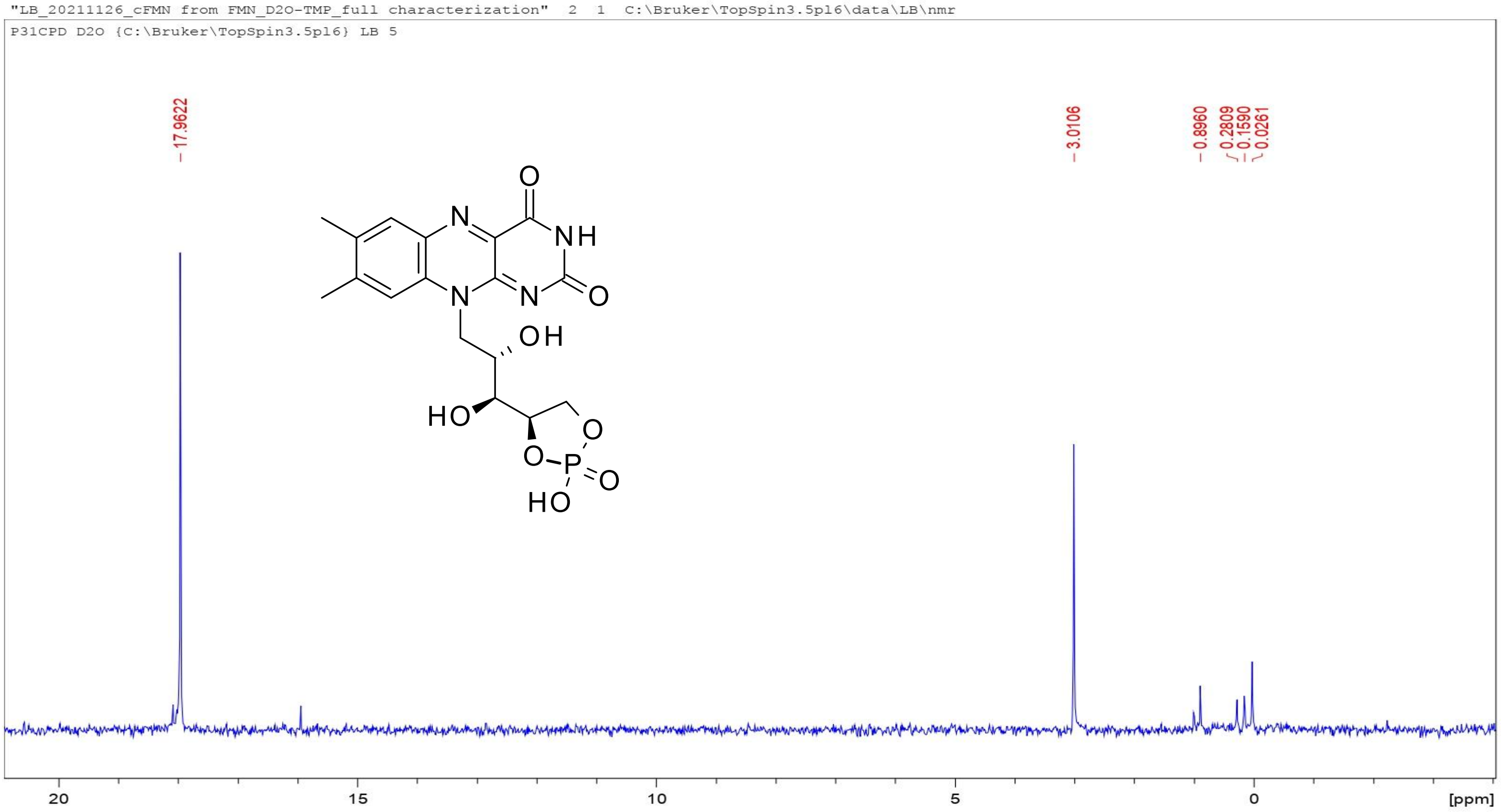

Compound 34. 162 MHz ^{31}P NMR spectrum in D_2O

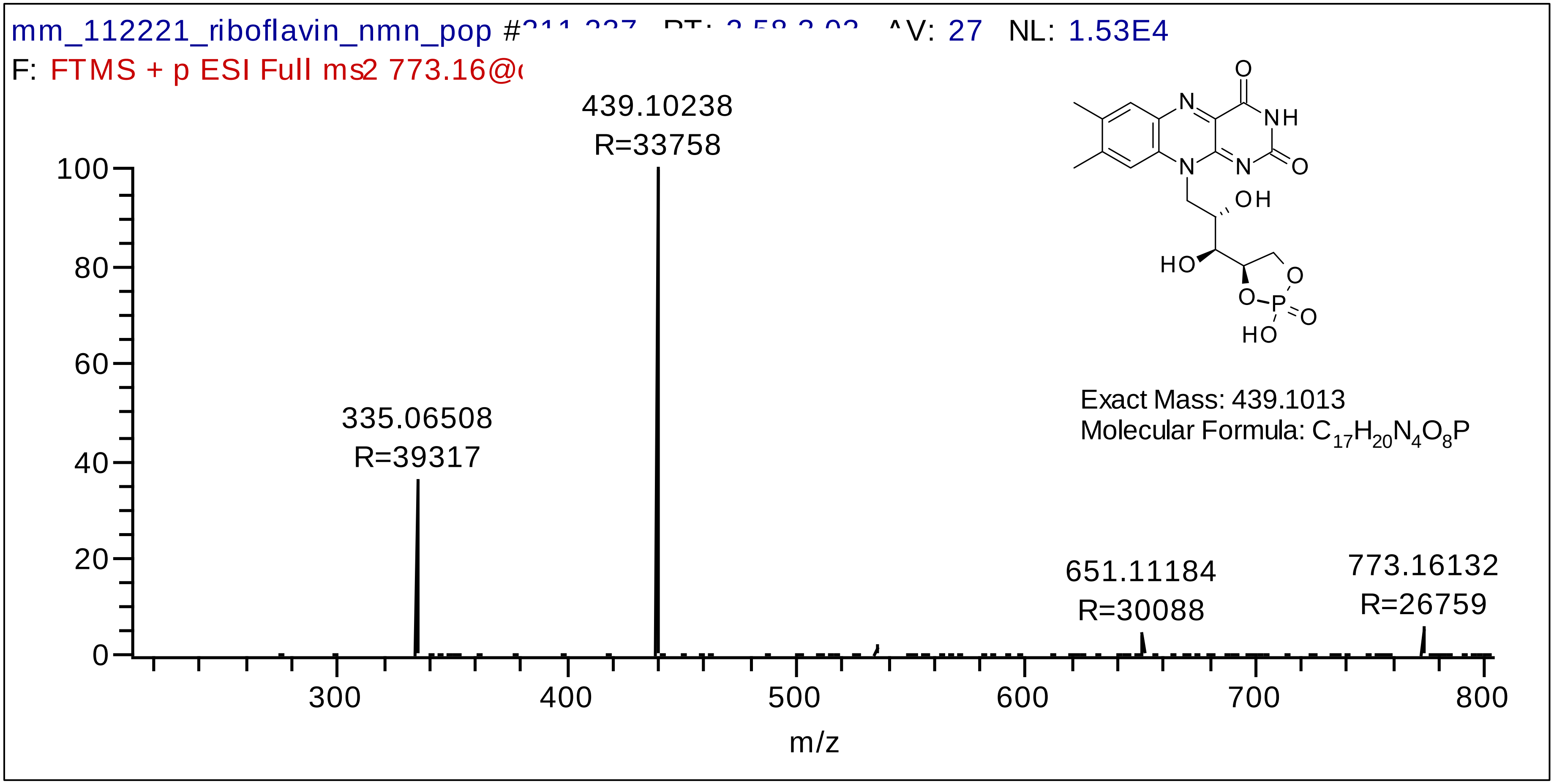

Compound 34. HRMS spectra

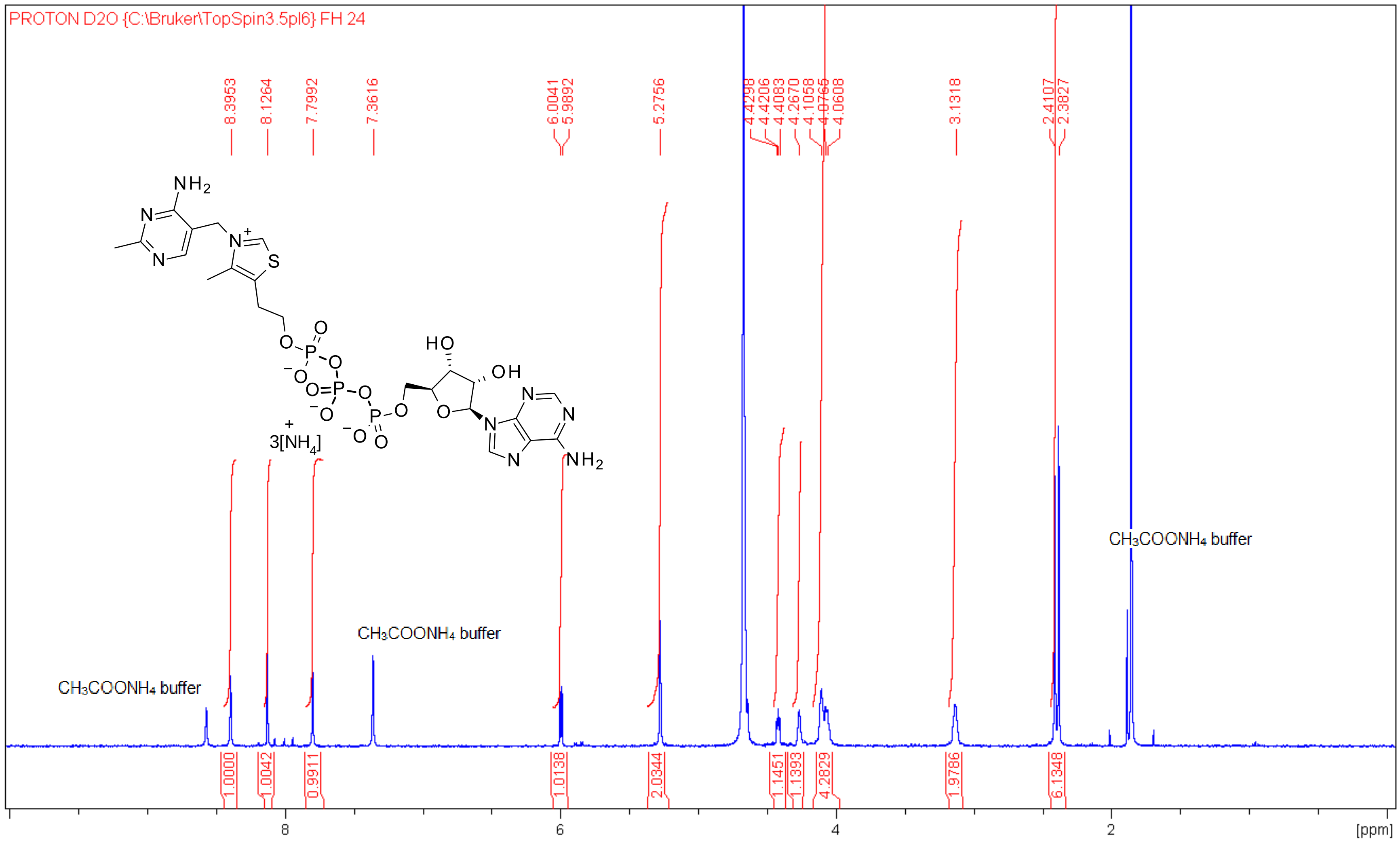

Compound 37. 400 MHz ^{1}H NMR spectrum in D_2O

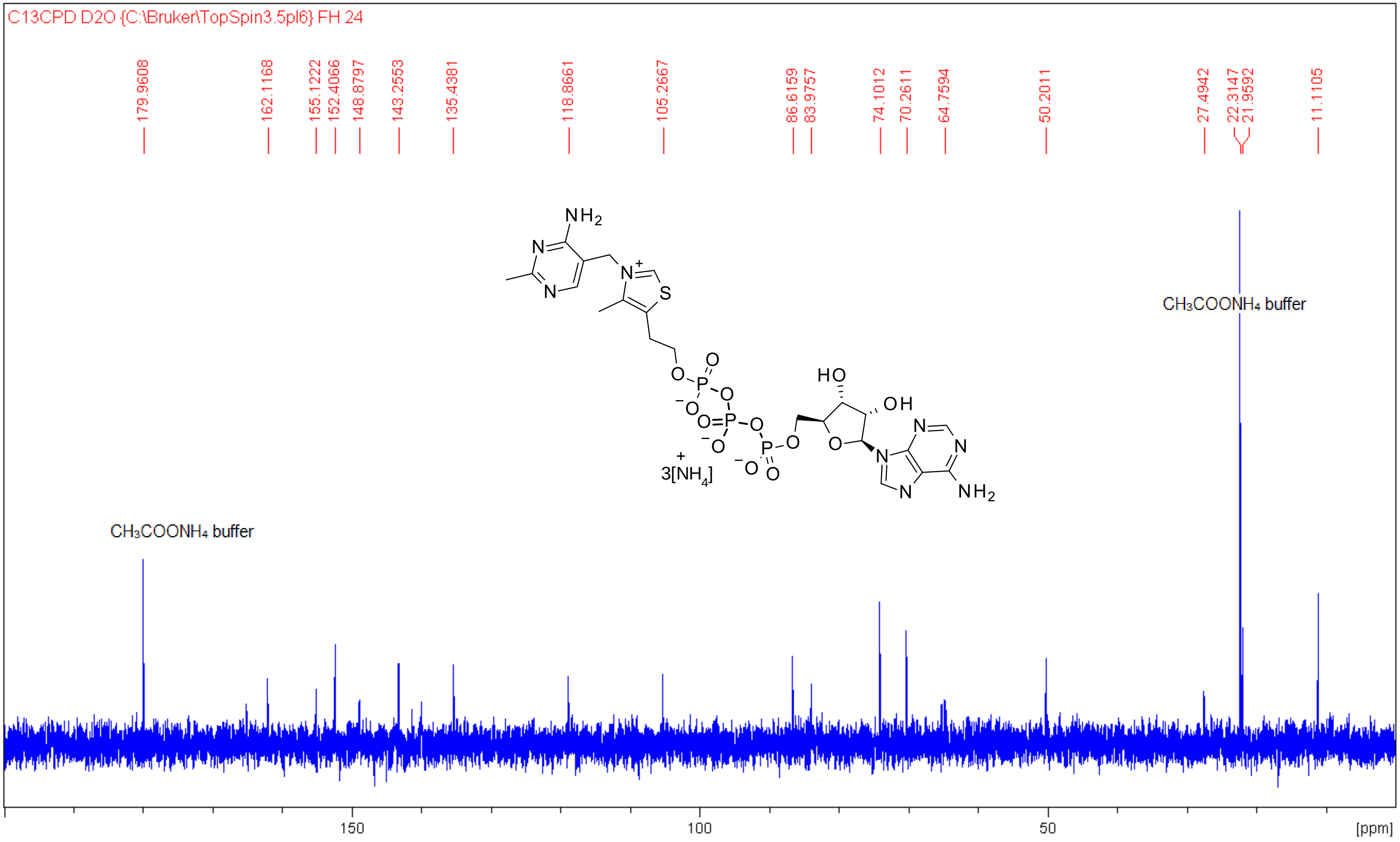

Compound 37. 100 MHz ^{13}C NMR spectrum in D_2O

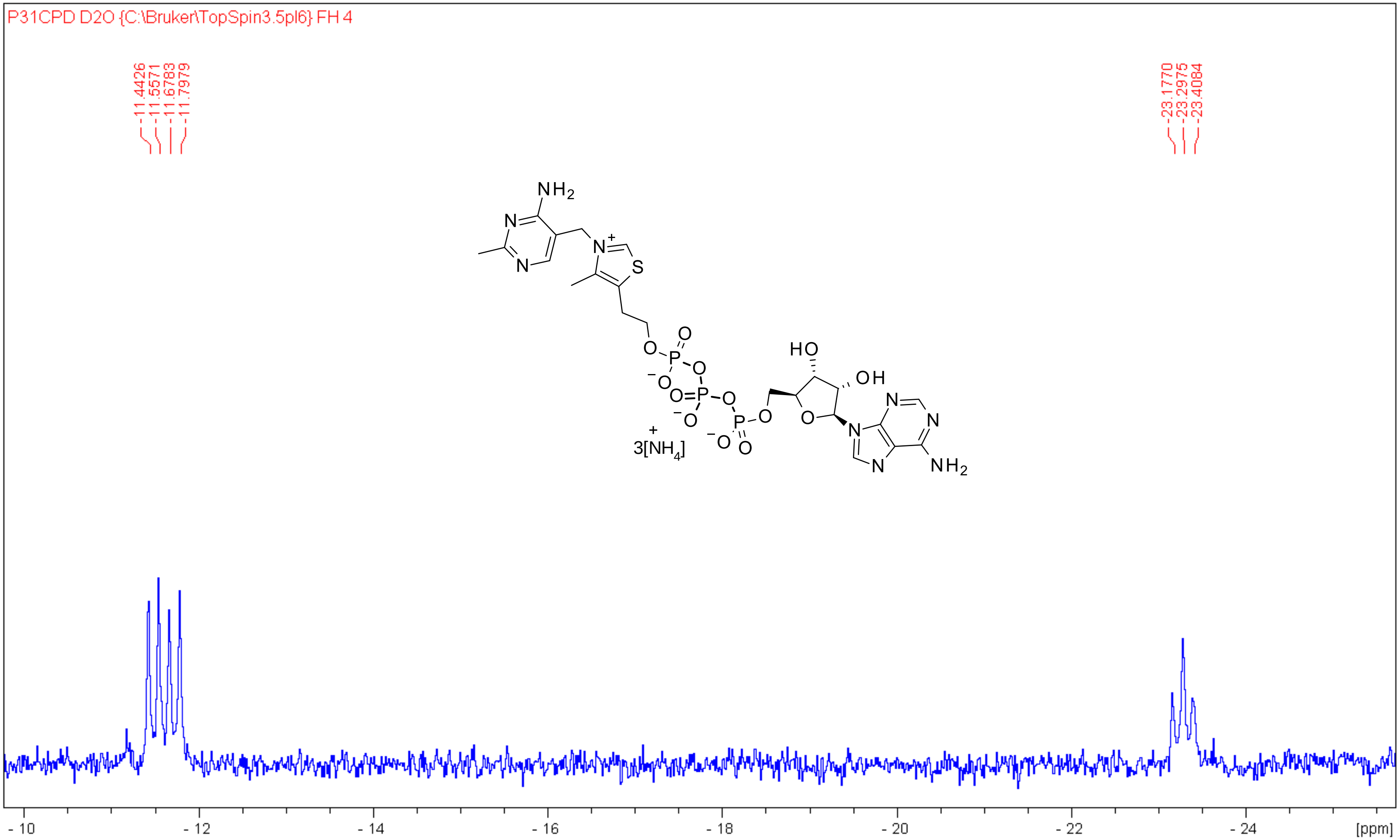

Compound 37. 162 MHz ^{31}P NMR spectrum in D_2O

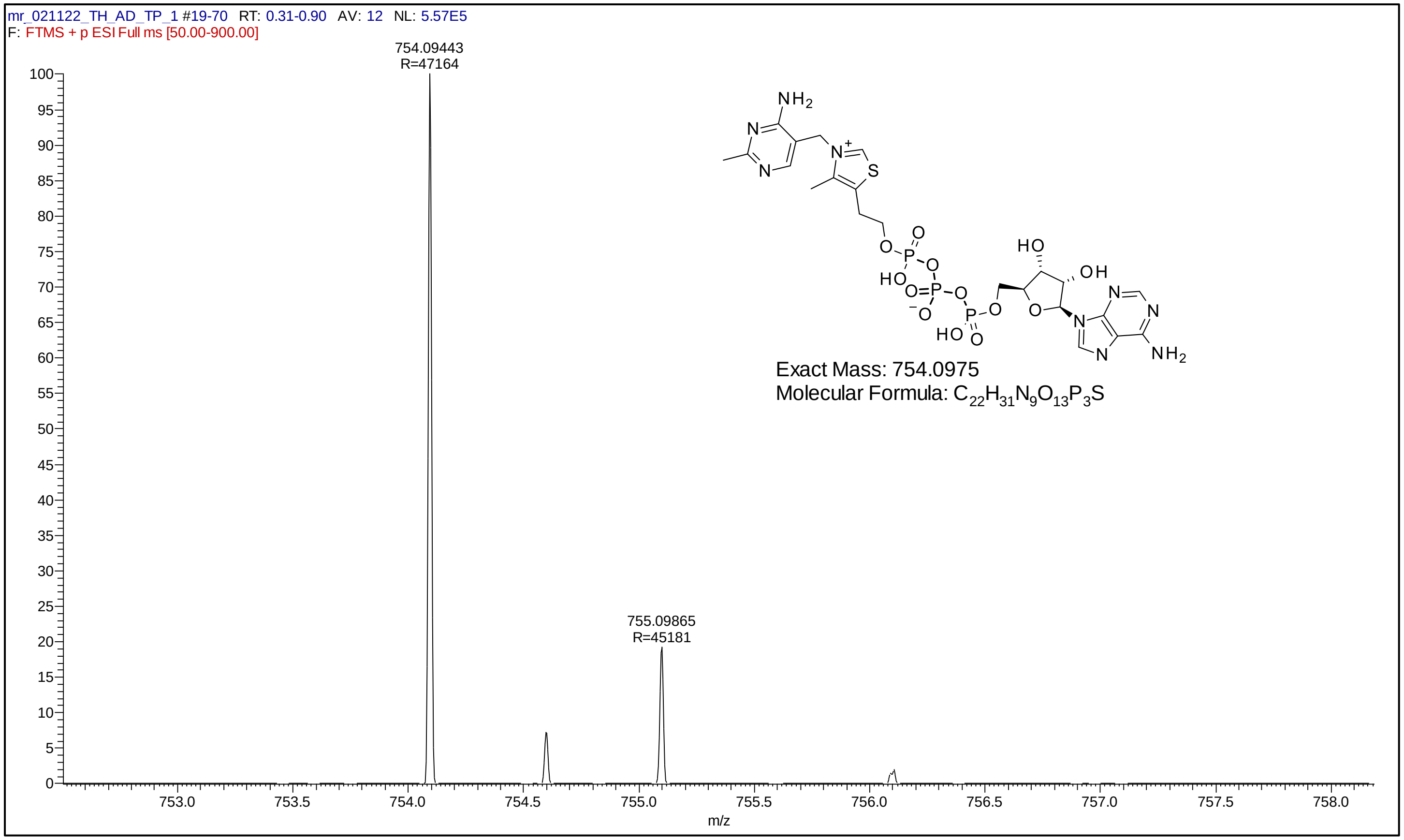

Compound 37. HRMS spectra

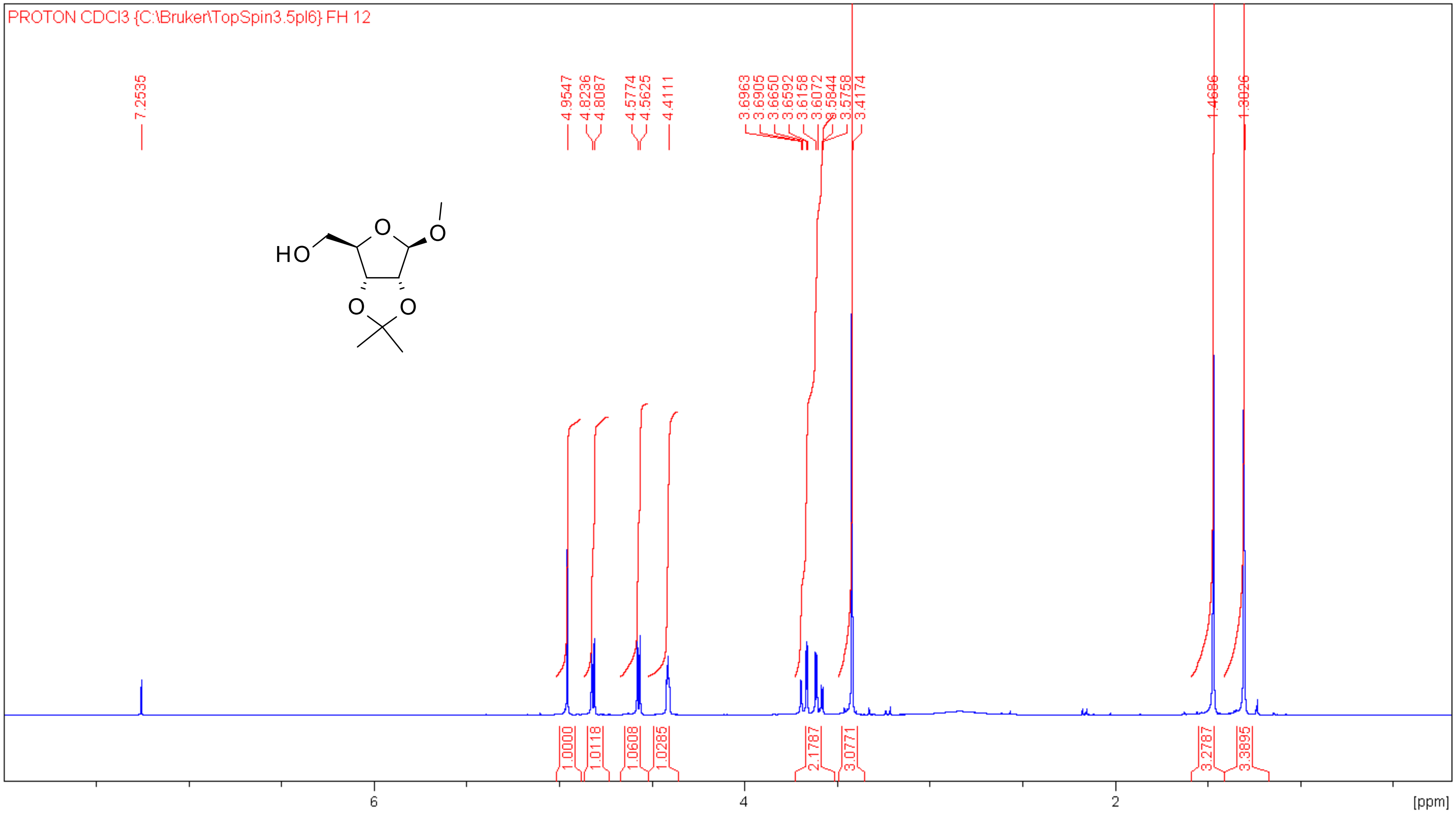

Compound 39. 400 MHz ^{1}H NMR spectrum in $CDCl_3$

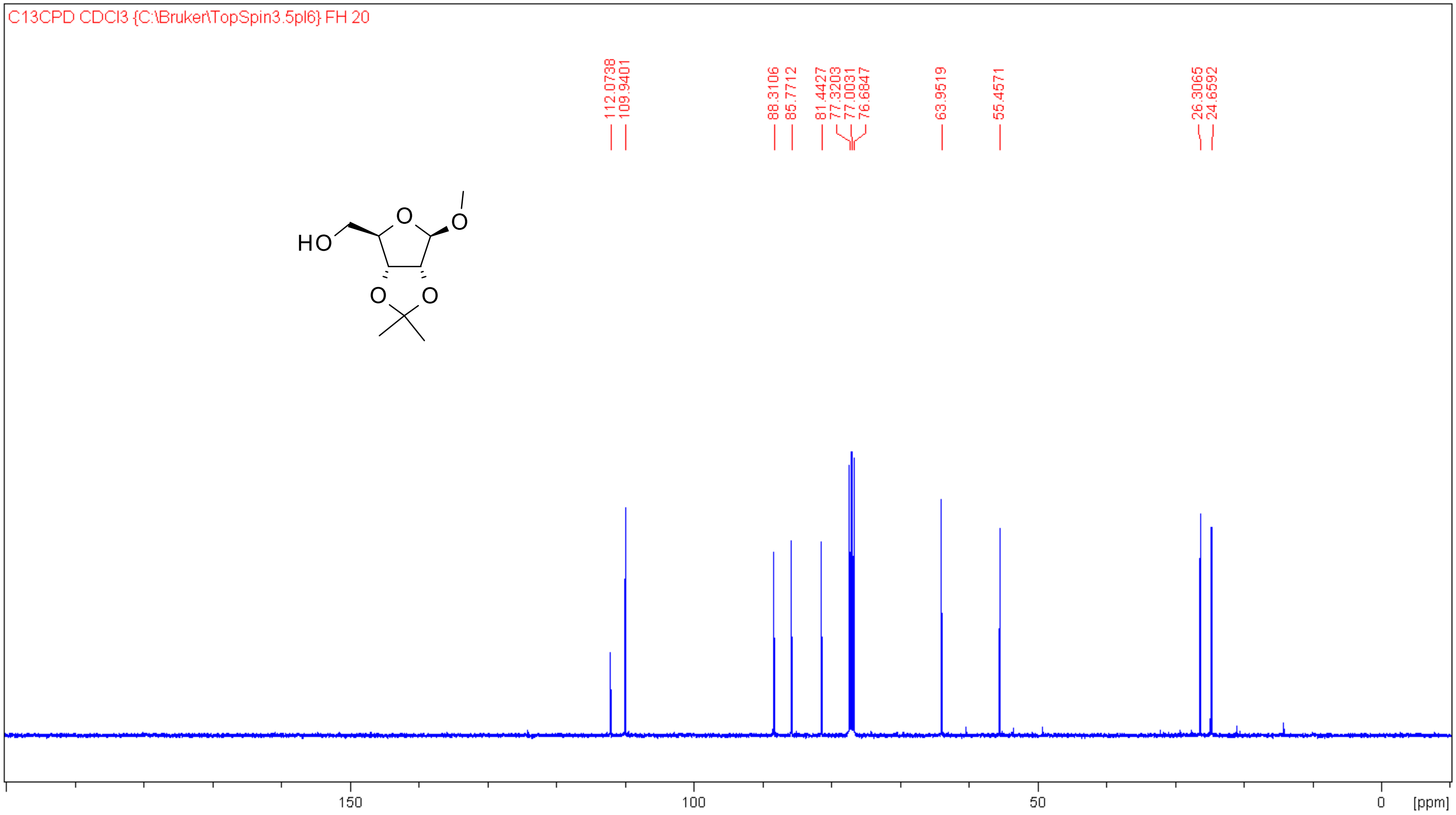

Compound 39. 100 MHz ^{31}C NMR spectrum in $CDCl_3$

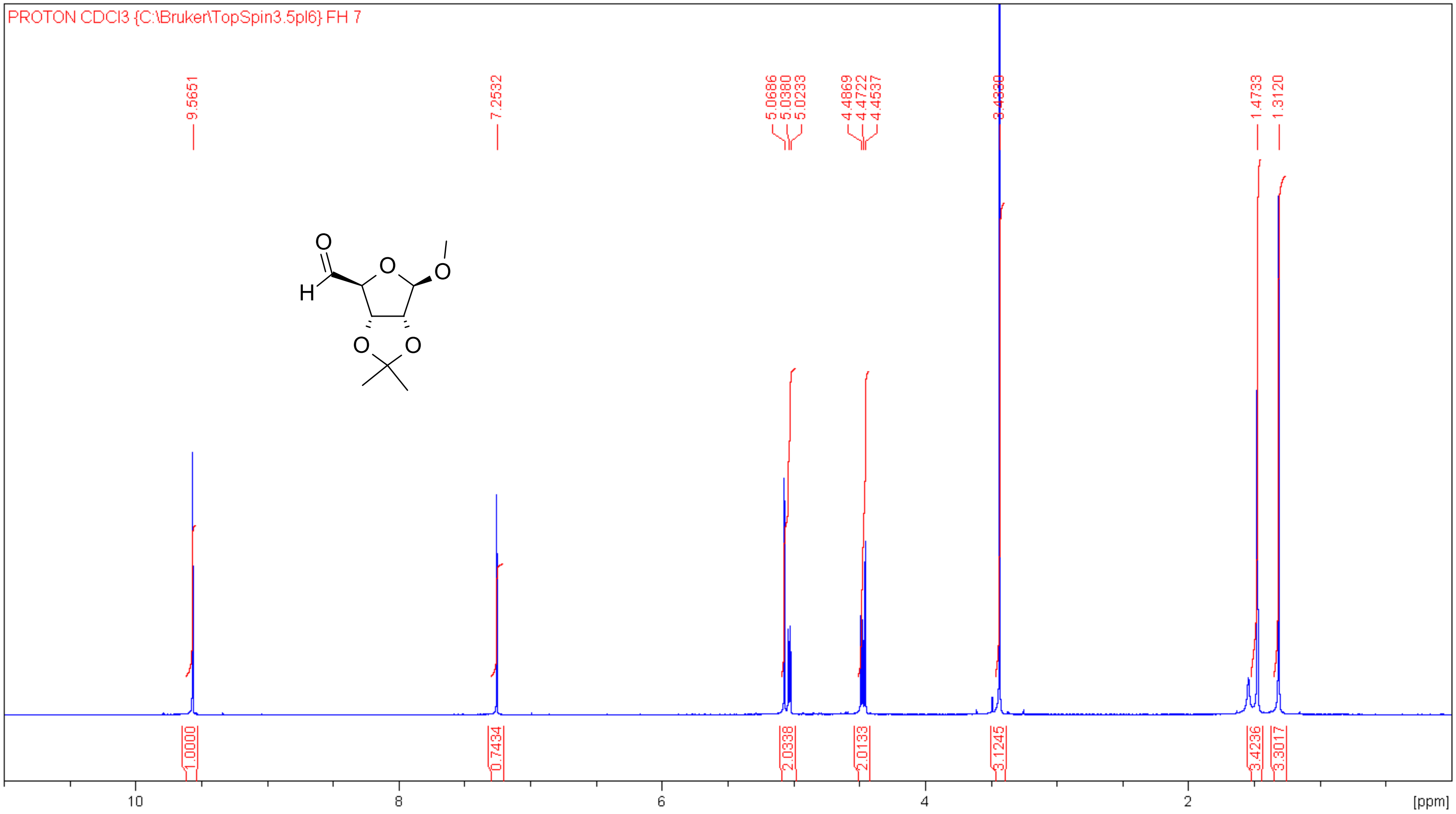

Compound 39. 400 MHz ^{1}H NMR spectrum in $CDCl_3$

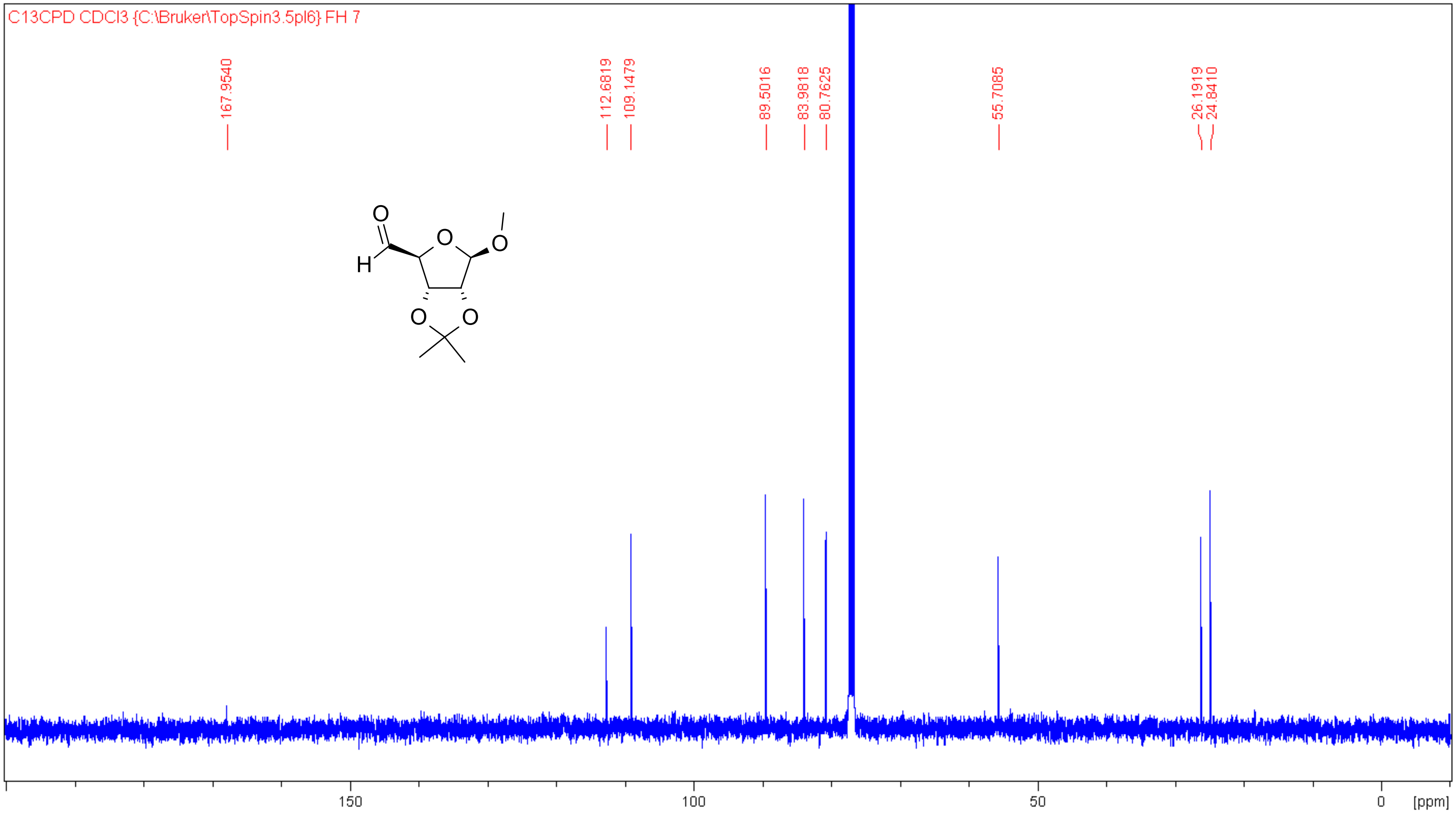

Compound 40. 100 MHz ^{31}C NMR spectrum in $CDCl_3$

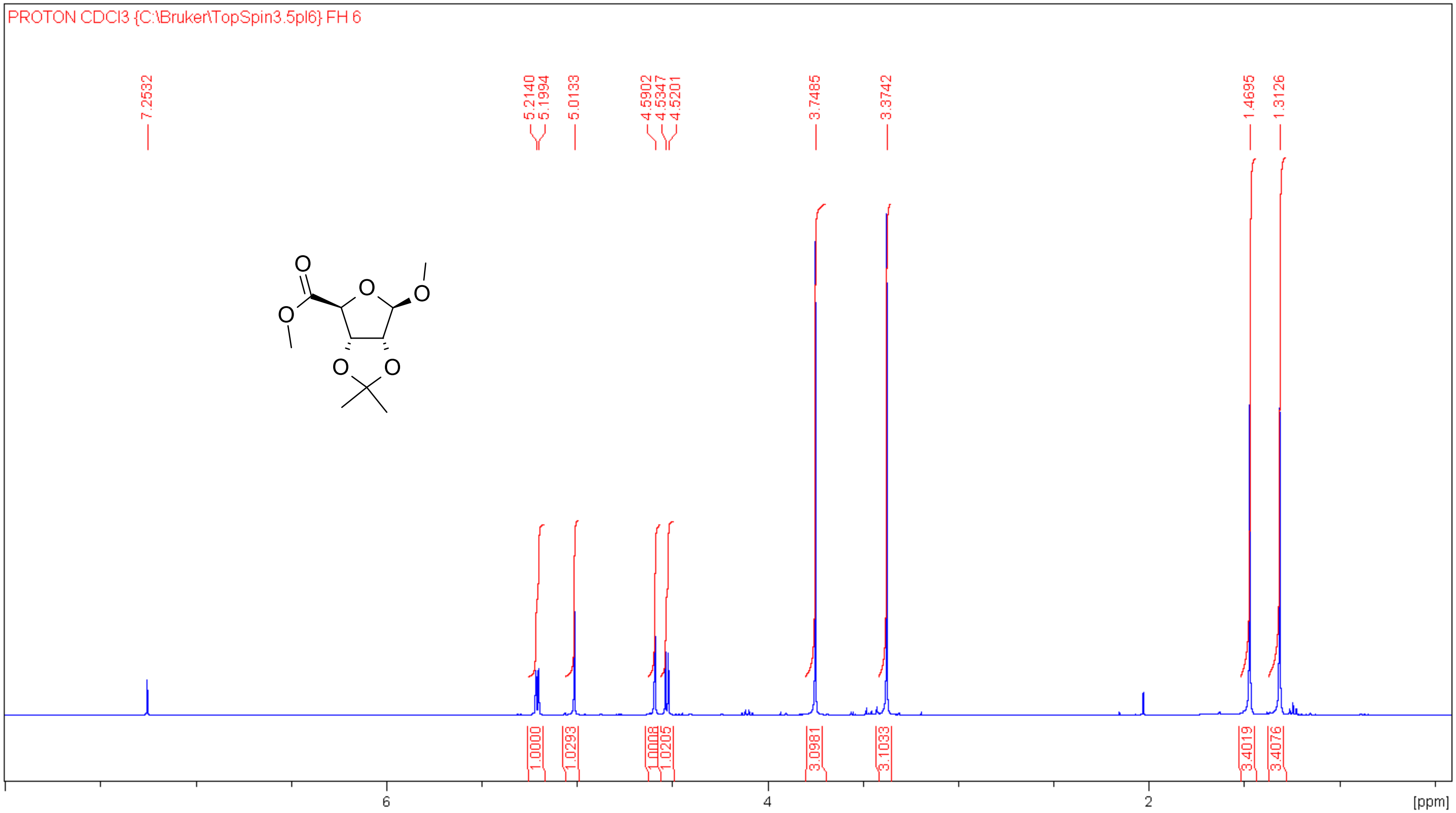

Compound 41. 400 MHz ^{1}H NMR spectrum in $CDCl_3$

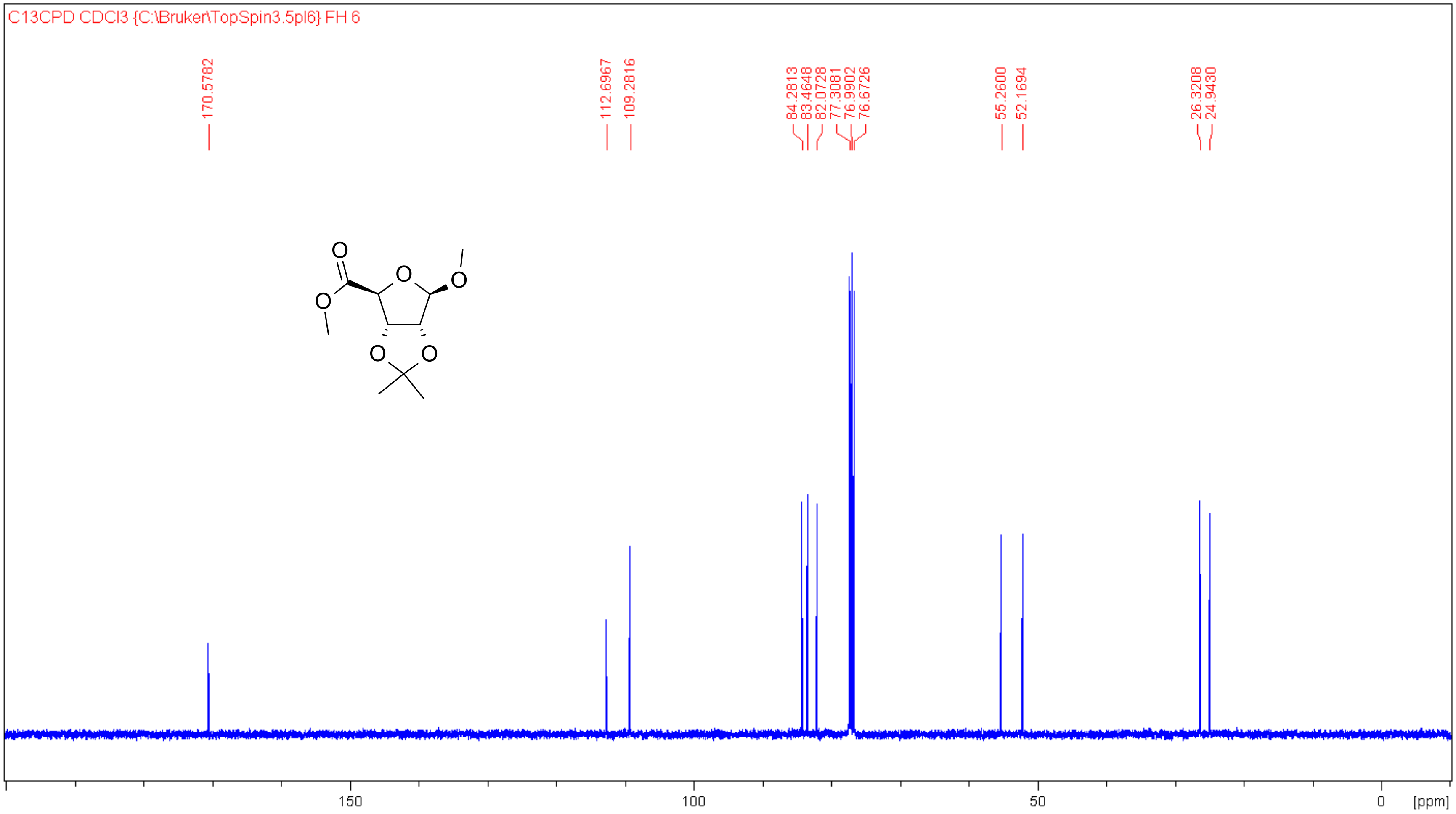

Compound 41. 100 MHz ^{31}C NMR spectrum in $CDCl_3$

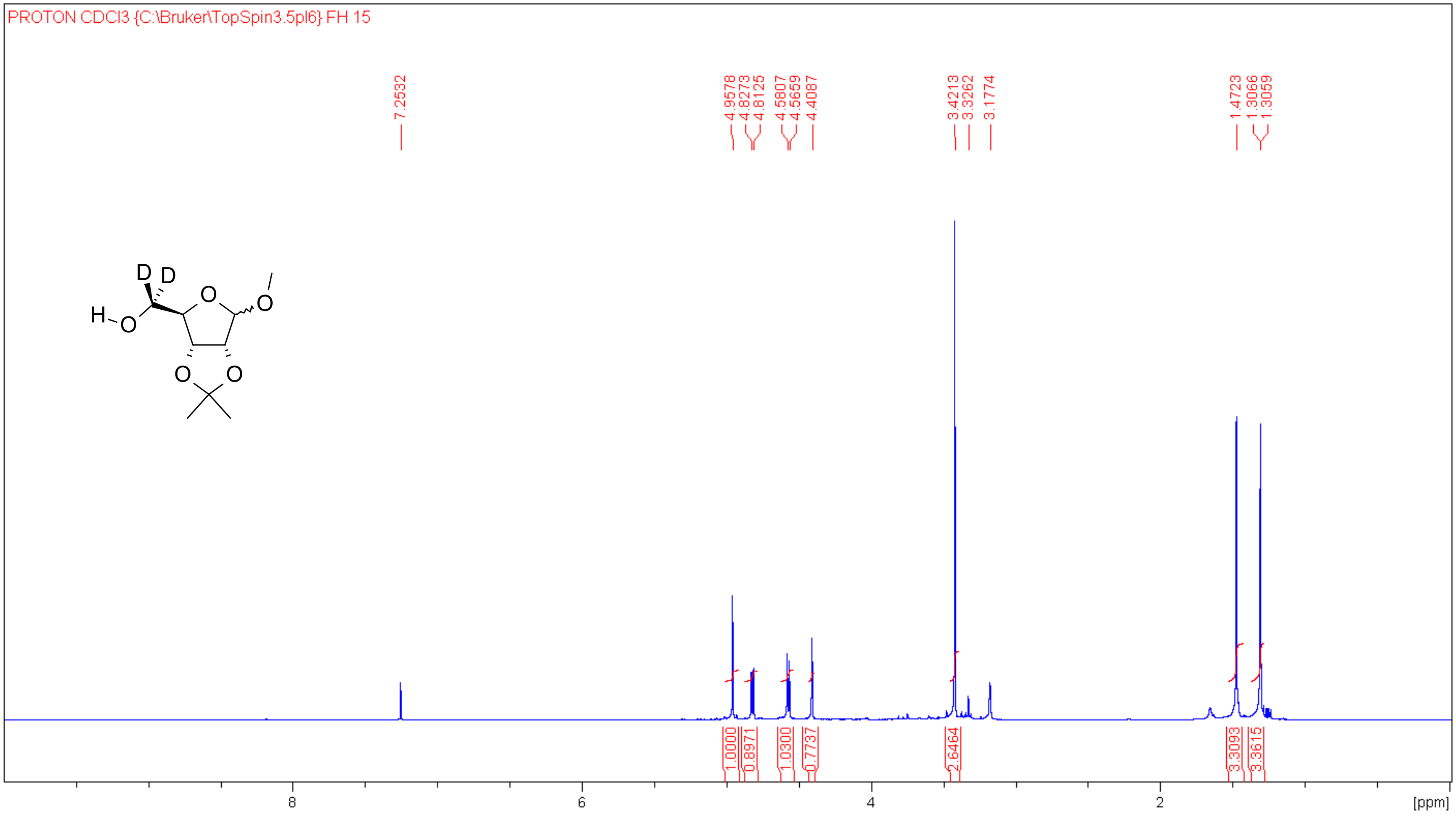

Compound 42. 400 MHz ^{1}H NMR spectrum in $CDCl_3$

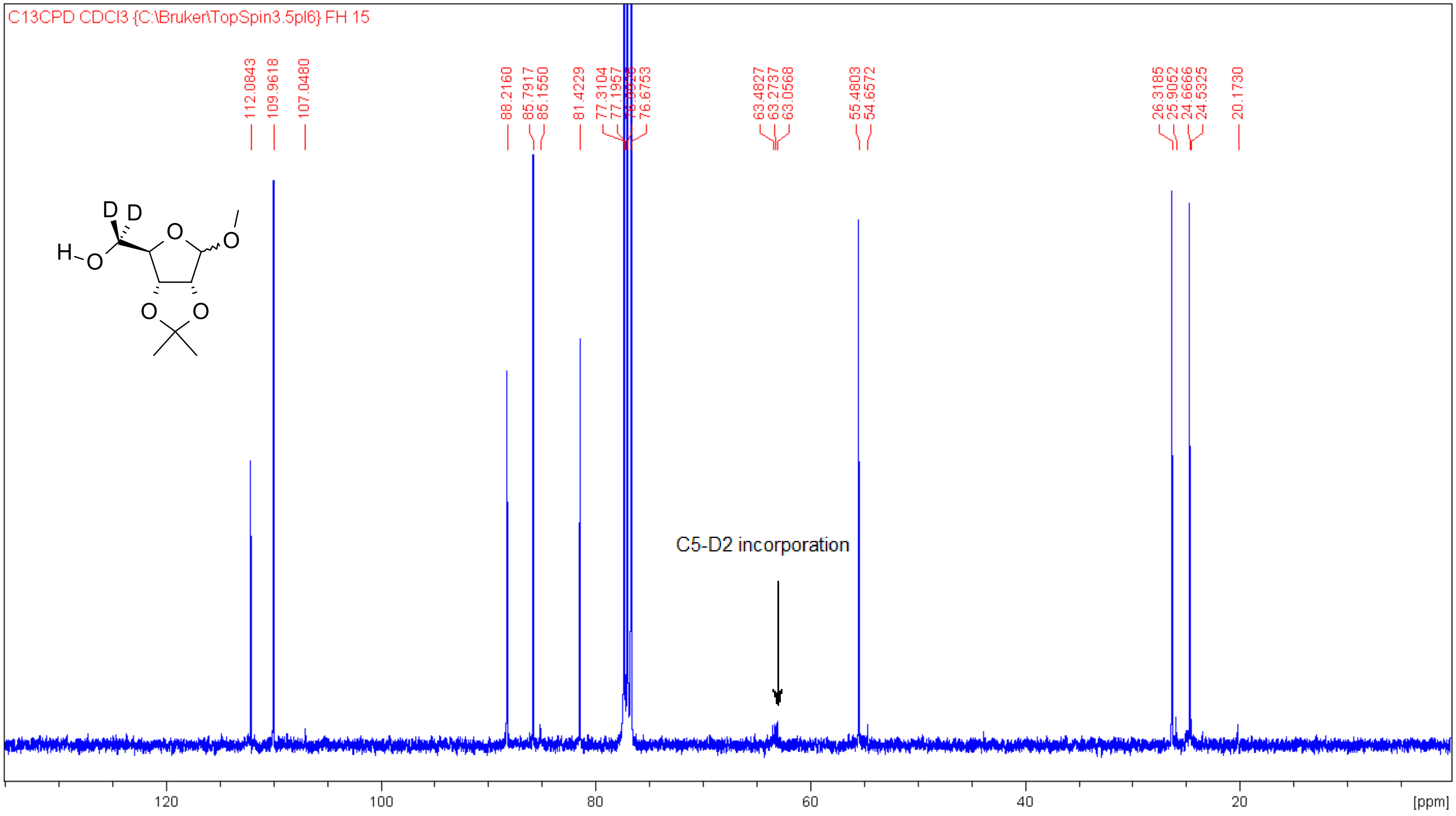

Compound 42. 100 MHz ^{31}C NMR spectrum in $CDCl_3$

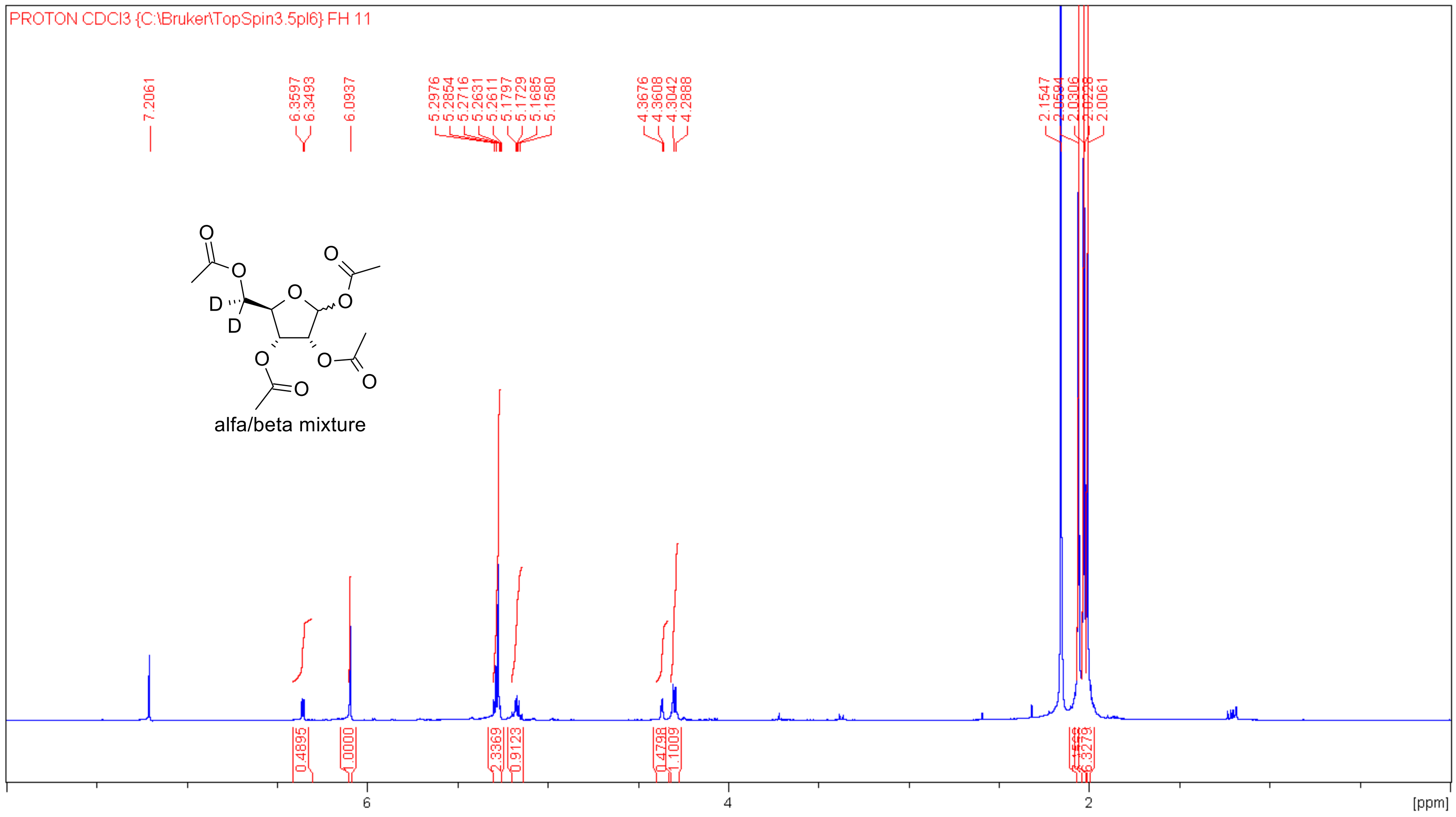

Compound 46. 400 MHz ^{1}H NMR spectrum in $CDCl_3$

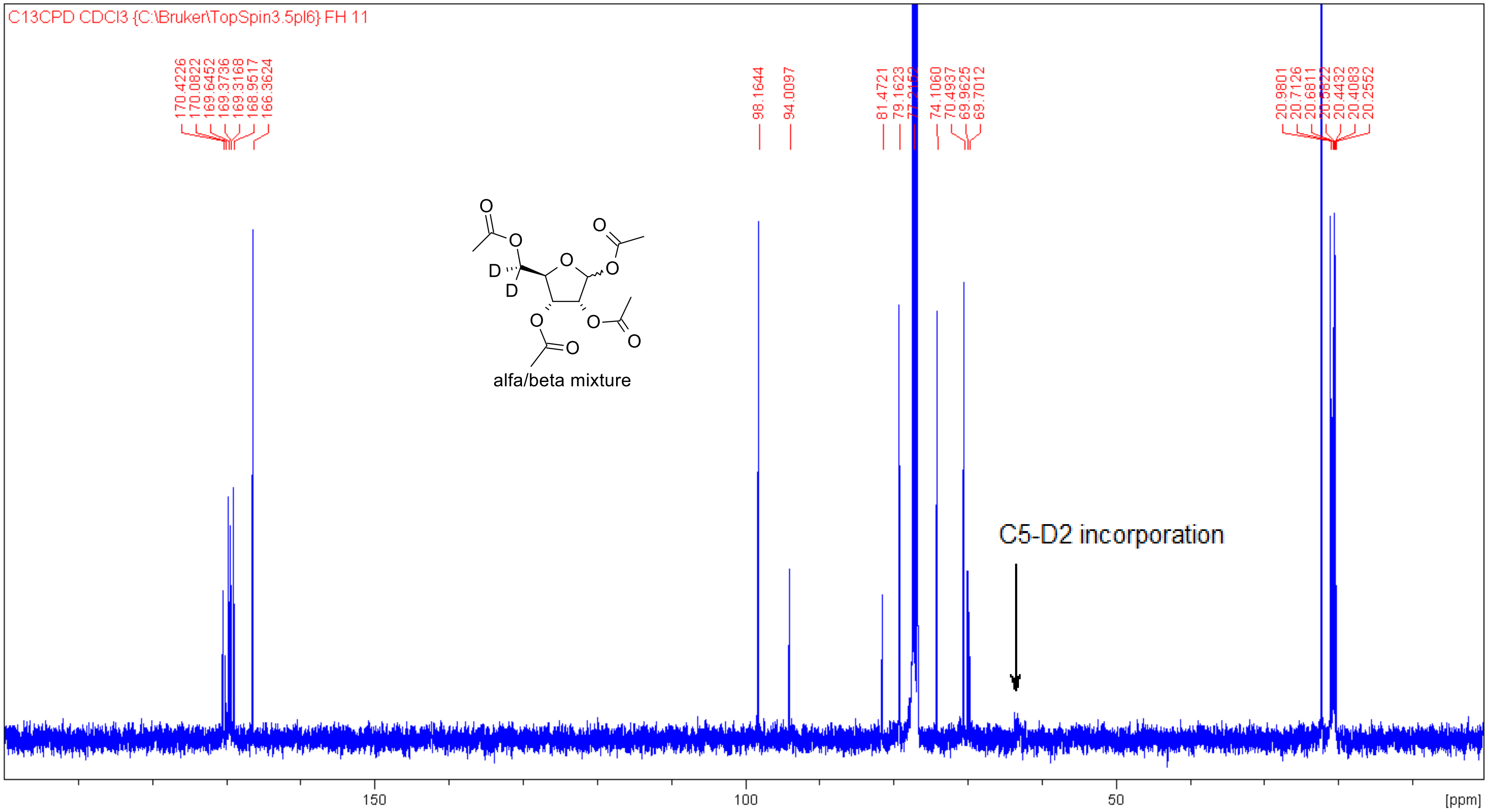

Compound 46. 100 MHz ^{31}C NMR spectrum in $CDCl_3$

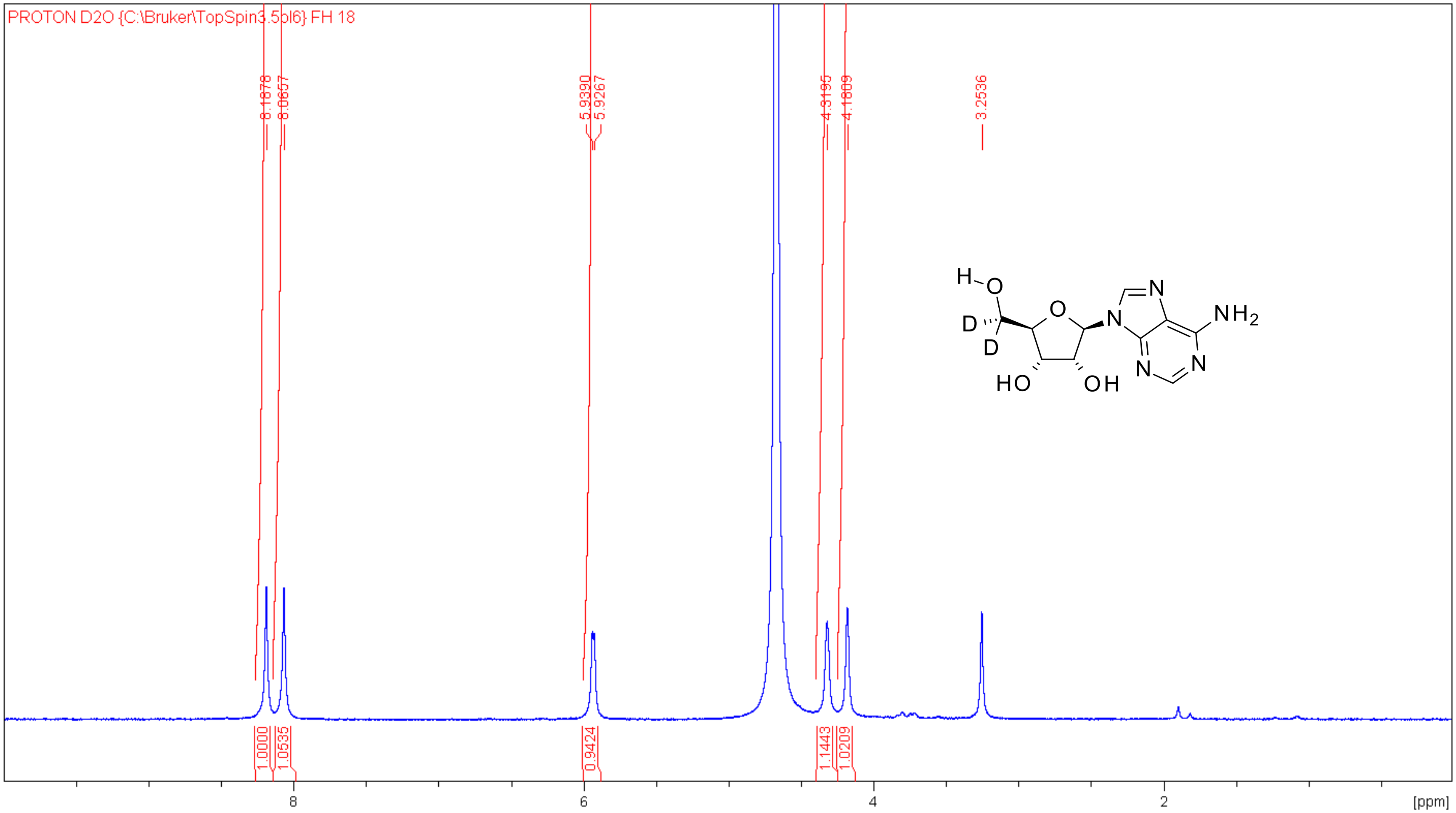

Compound 48. 400 MHz ^{1}H NMR spectrum in D_2O

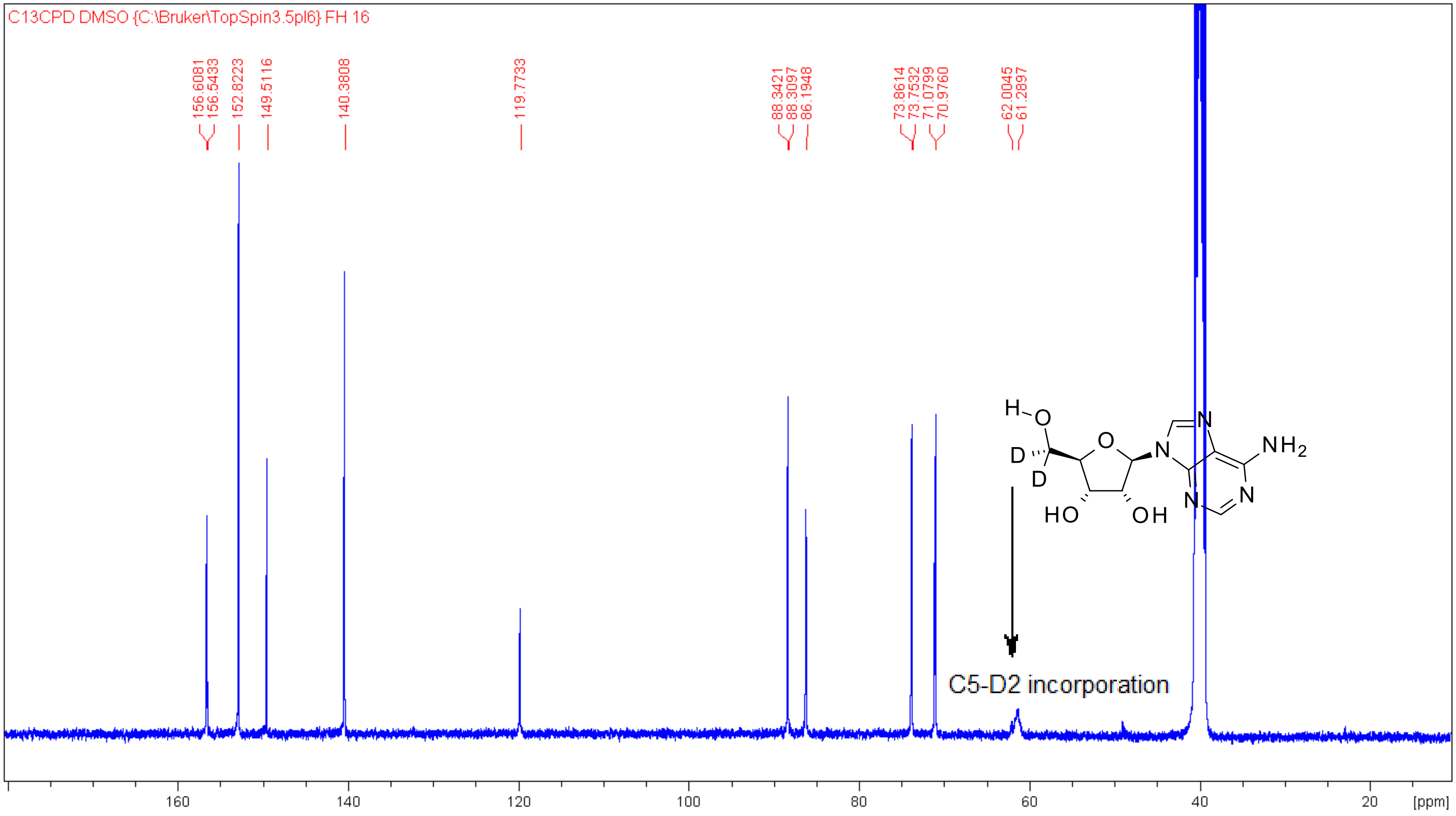

Compound 48. 100 MHz ^{31}C NMR spectrum in D_2O

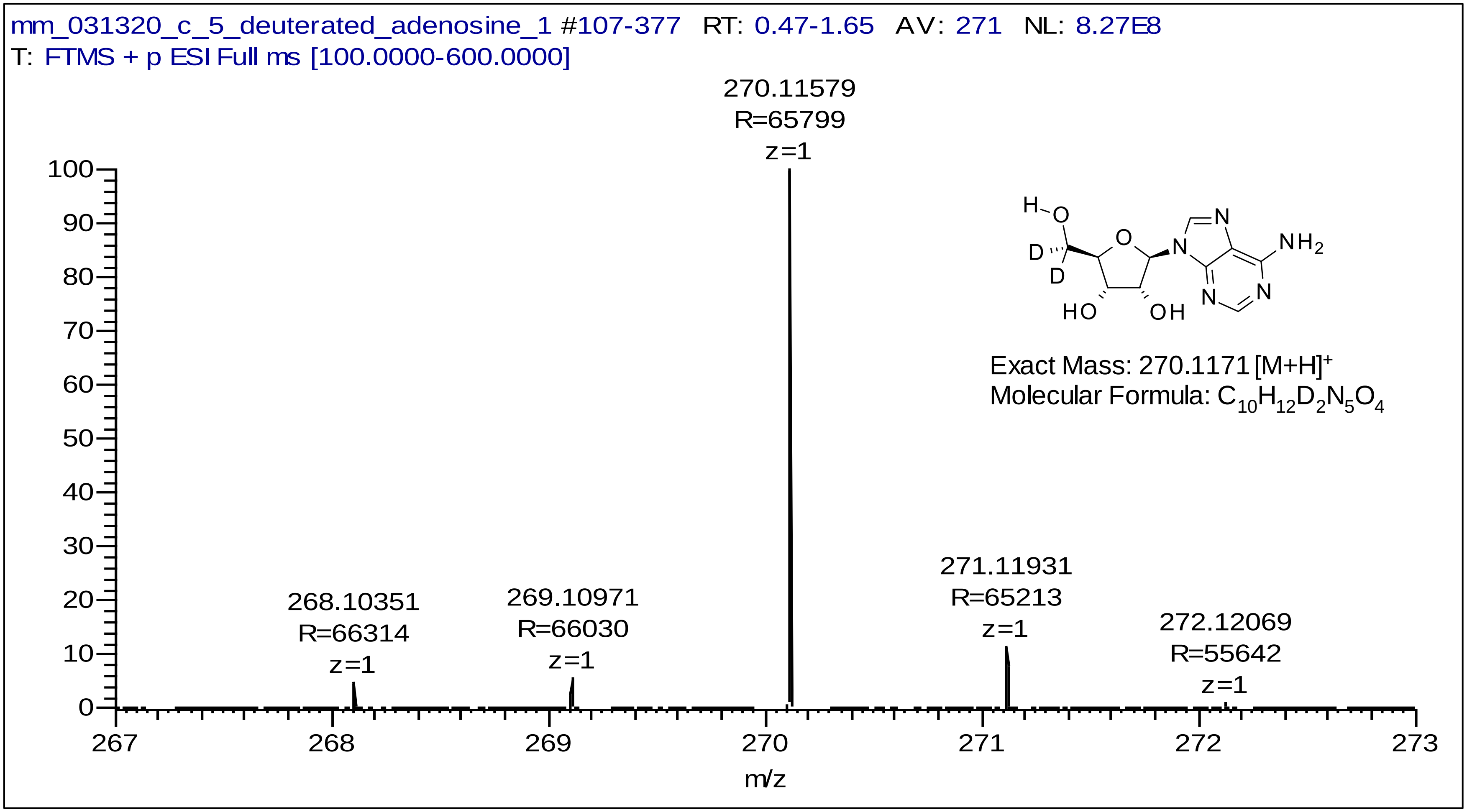

Compound 48. HRMS Spectra

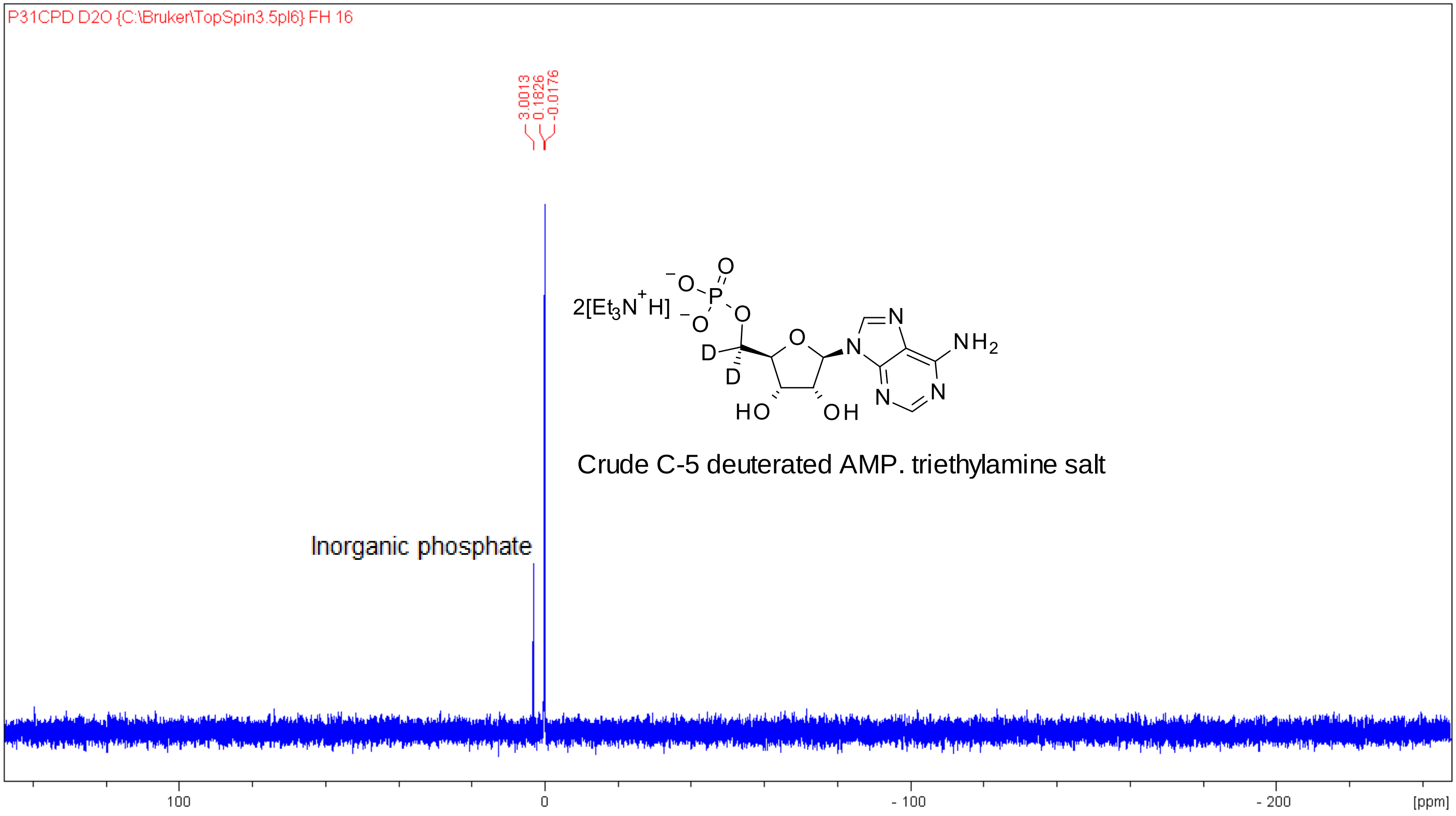

Compound 49. 162 MHz ^{31}P NMR spectrum in D_2O

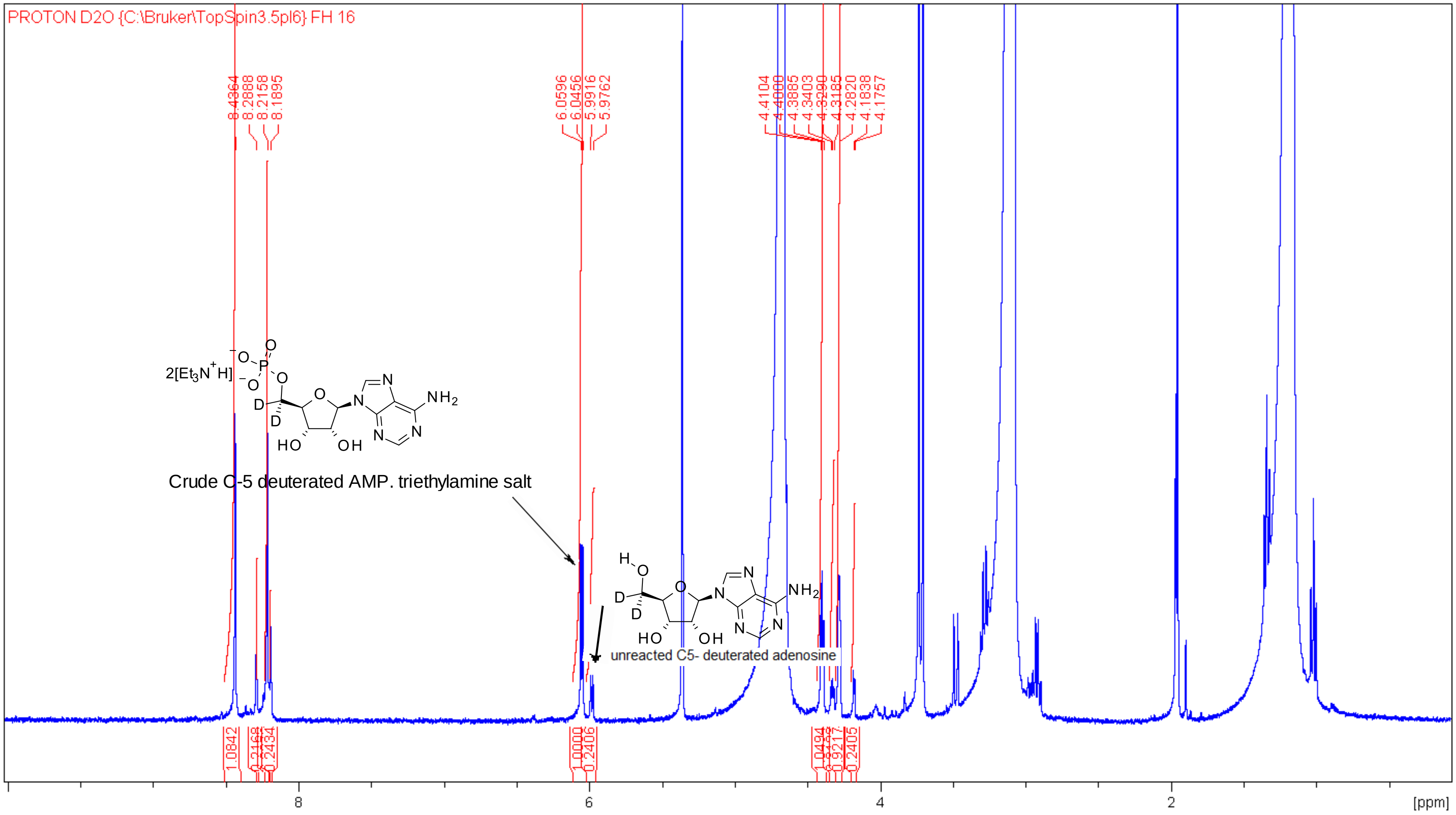

Compound 49. 400 MHz ^{1}H NMR spectrum in D_2O

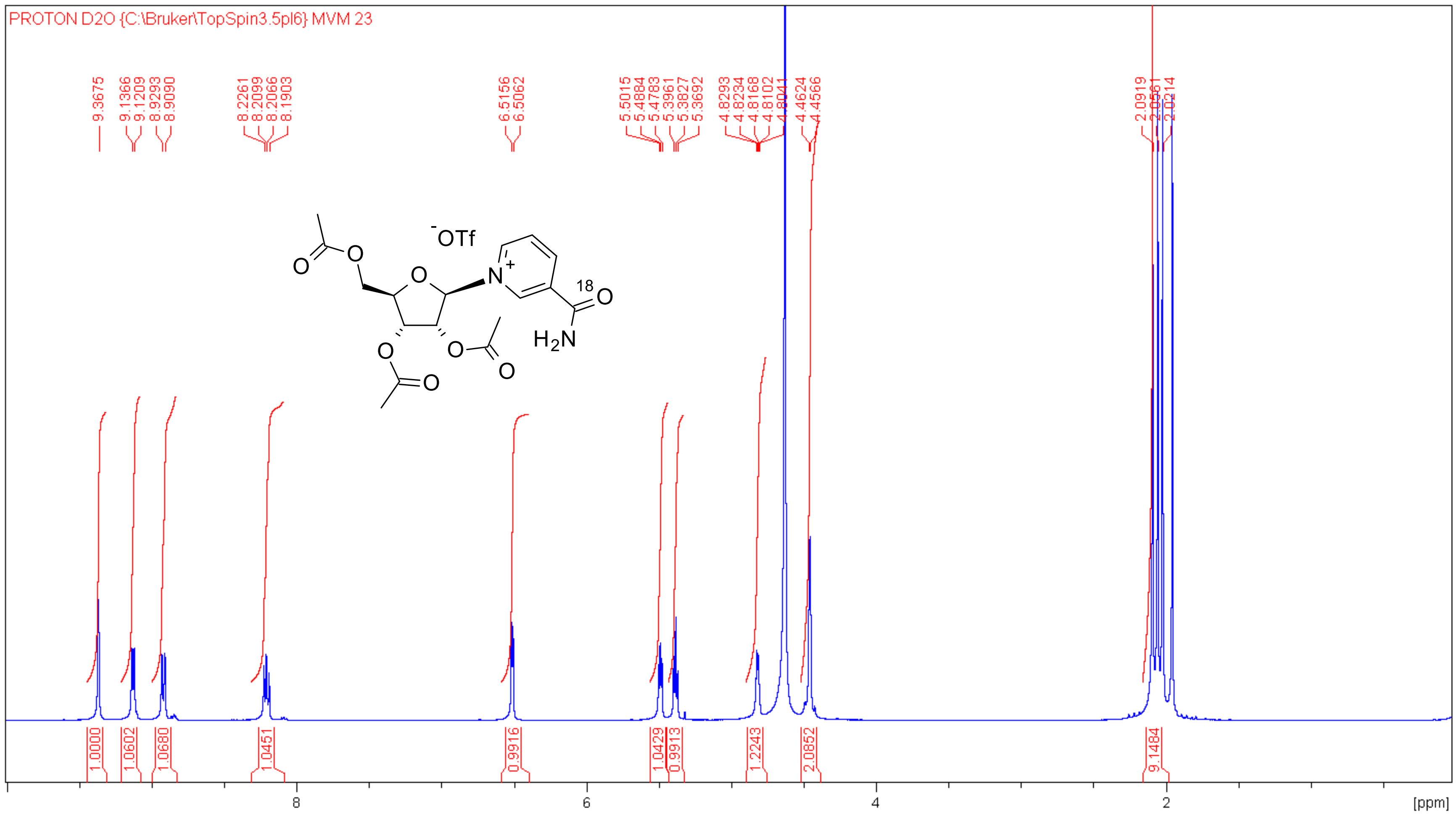

Compound 53. 400 MHz ^{1}H NMR spectrum in D_2O

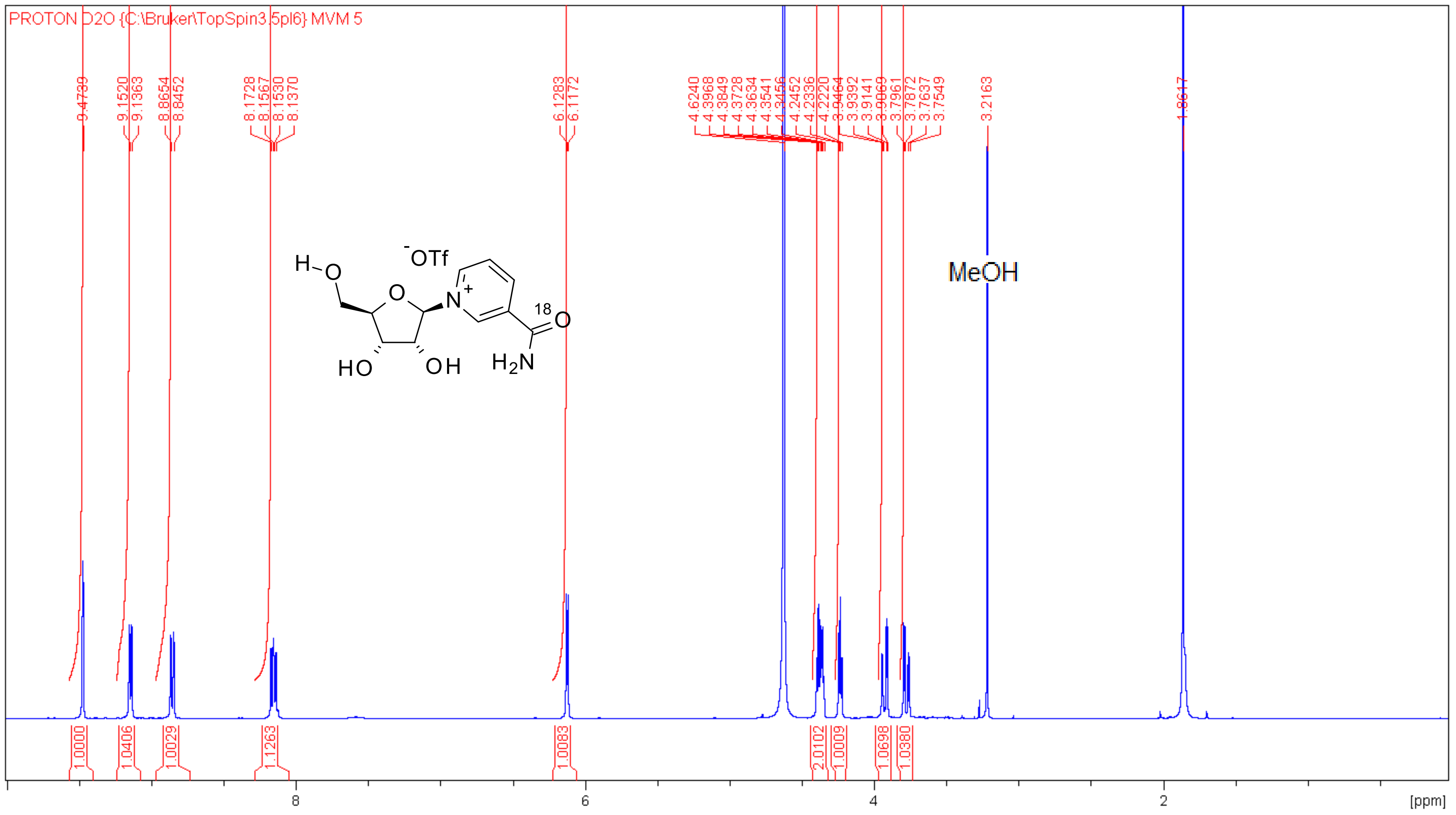

Compound 54. 400 MHz ^{1}H NMR spectrum in D_2O

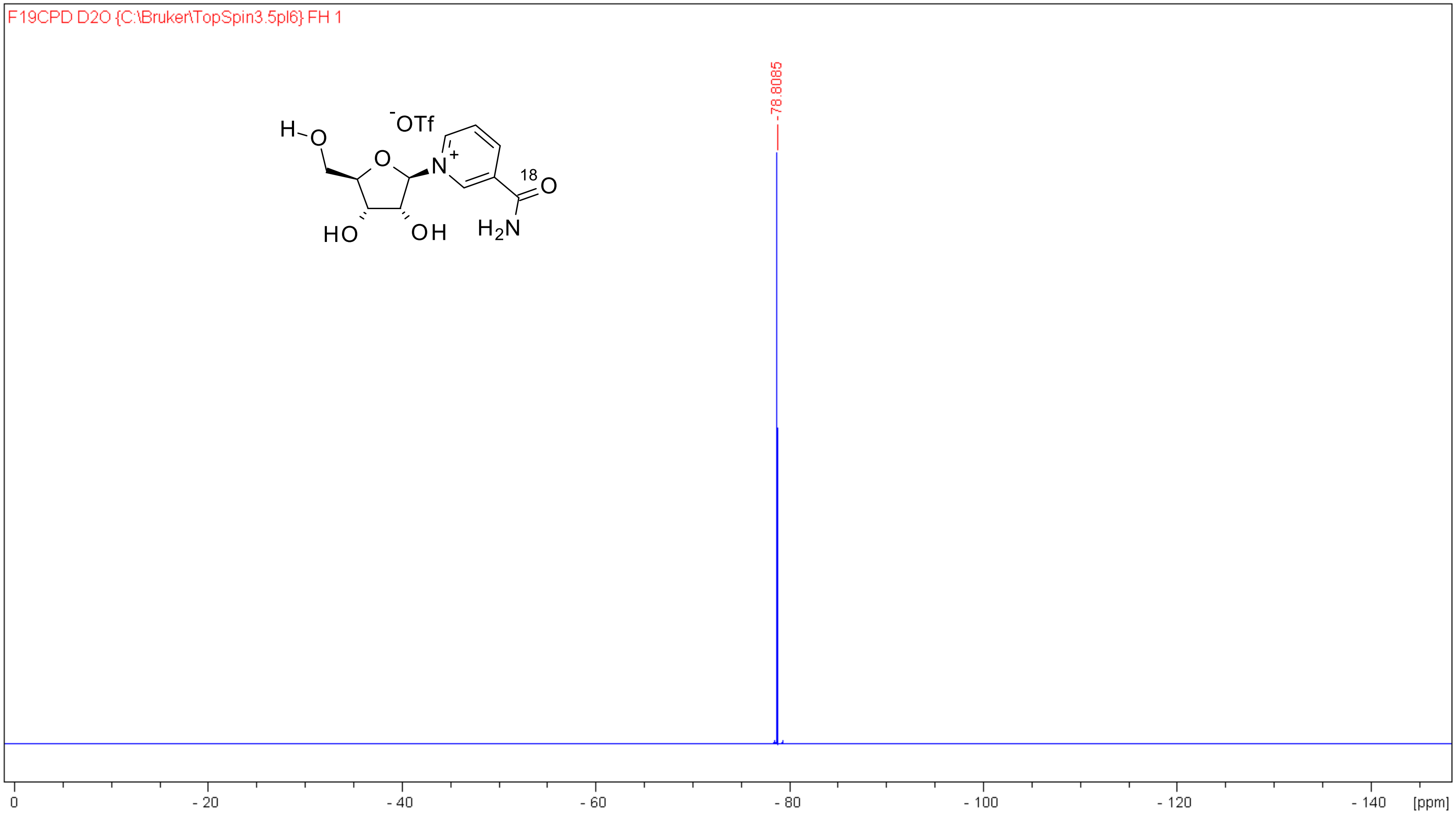

Compound 54. 377 MHz ^{19}F NMR spectrum in D_2O
